# Mother-to-child transmission of Chikungunya virus: A systematic review and meta-analysis

**DOI:** 10.1371/journal.pntd.0006510

**Published:** 2018-06-13

**Authors:** Despina Contopoulos-Ioannidis, Shoshana Newman-Lindsay, Camille Chow, A. Desiree LaBeaud

**Affiliations:** 1 Department of Pediatrics, Division of Infectious Diseases, Stanford University School of Medicine, Stanford, CA, United States of America; 2 Department of Pediatrics, Children's Hospital of Richmond, Virginia Commonwealth University, Richmond, VA, United States of America; 3 Department of Internal Medicine, St. Agnes Medical Center, Fresno, CA, United States of America; Fundacao Oswaldo Cruz, BRAZIL

## Abstract

**Background:**

Chikungunya virus (CHIKV) is an emerging arboviral infection with a global distribution and may cause fetal and neonatal infections after maternal CHIKV-infections during gestation.

**Methodology:**

We performed a systematic review to evaluate the risk for: a) mother-to-child transmission (MTCT), b) antepartum fetal deaths (APFD), c) symptomatic neonatal disease, and d) neonatal deaths from maternal CHIKV-infections during gestation. We also recorded the neonatal clinical manifestations after such maternal infections (qualitative data synthesis). We searched PubMed (last search 3/2017) for articles, of any study design, with any of the above outcomes. We calculated the overall risk of MTCT, APFDs and risk of symptomatic neonatal disease by simple pooling. For endpoints with ≥5 events in more than one study, we also synthesized the data by random-effect-model (REM) meta-analysis.

**Principal findings:**

Among 563 identified articles, 13 articles from 8 cohorts were included in the quantitative data synthesis and 33 articles in the qualitative data synthesis. Most cohorts reported data only on symptomatic rather than on all neonatal infections. By extrapolation also of these data, the overall pooled-MTCT-risk across cohorts was at least 15.5% (206/1331), (12.6% by REMs). The pooled APFD-risk was 1.7% (20/1203); while the risk of CHIKV-confirmed-APFDs was 0.3% (3/1203). Overall, the pooled-risk of symptomatic neonatal disease was 15.3% (203/1331), (11.9% by REMs). The pooled risk of symptomatic disease was 50.0% (23/46) among intrapartum vs 0% (0/712) among antepartum/peripartum maternal infections. Infected newborns, from maternal infections during gestation were either asymptomatic or presented within their first week of life, but not at birth, with fever, irritability, hyperalgesia, diffuse limb edema, rashes and occasionally sepsis-like illness and meningoencephalitis. The pooled-risk of neonatal death was 0.6% (5/832) among maternal infections and 2.8% (5/182) among neonatal infections; long-term neurodevelopmental delays occurred in 50% of symptomatic neonatal infections.

**Conclusions/Significance:**

Published cohorts with data on the risk to the fetus and/or newborn from maternal CHIKV-infections during gestation were sparse compared to the number of recently reported CHIKV-infection outbreaks worldwide; however perinatal infections do occur, at high rates during intrapartum period, and can be related to neonatal death and long-term disabilities.

## Author summary

Chikungunya virus (CHIKV) is an emerging arboviral infection with a global distribution and can cause infections of the fetus and newborn after maternal CHIKV-infections during gestation. In this systematic review, we evaluated the risk for mother-to-child transmission (MTCT), antepartum fetal deaths (APFD) and symptomatic neonatal disease from maternal CHIKV-infections during gestation. Whenever meaningful, we also synthesized the data by random-effect-model (REM) meta-analysis. We also recorded the list of clinical manifestations of neonatal infections after maternal infections during gestation. Overall, published cohorts with pertinent data to estimate the impact to the fetuses and newborns of maternal CHIKV-infections were sparse compared to the number of recently reported CHIKV-infection outbreaks worldwide. Most cohorts reported data only on symptomatic neonatal infections rather than on all (symptomatic and asymptomatic) neonatal infections. By extrapolation also of these data, the pooled MTCT-risk was at least 15.5% (206/1331), (12.6% by REMs). Symptomatic disease occurred almost exclusively with maternal infections around the time of delivery. Overall, the pooled risk of symptomatic disease was 15.3% (203/1331), (11.9% by REMs); however, the risk of symptomatic disease from intrapartum maternal infections was 50.0% (23/46) vs 0% (0/712) from antepartum/peripartum maternal infections. The pooled APFDs-risk was low (1.7%); however, APFDs occurred with maternal infections in all trimesters. Infected newborns were either asymptomatic or presented during their first week of life, but not at the time of birth, with manifestations such as fever, irritability, rashes, hyperalgesia syndrome, diffuse limb edema, bullous dermatitis and occasionally also meningoencephalitis. Long-term neurodevelopmental delays occurred in 50% of symptomatic neonatal infections.

## Introduction

Chikungunya virus (CHIKV) is a remerging arbovirus[1–5] in the family of *Togaviridae*, genus *Alphavirus* that is transmitted by the *Aedes spp*. mosquitos *A*. *aegyptii* and *A*. *albopictus*[6] causing a crippling musculoskeletal inflammatory disease in humans characterized by fever, polyarthralgia, myalgia, rash, and headache.[7] It was first identified in Tanzania in 1953[8], and the name comes from a Makonde word that means “that which bends up” due to the position taken by patients suffering from the severe joint pain.[9, 10] Since then, it has caused outbreaks in Africa[5, 11, 12], Indian Ocean islands, South East Asia [13–15], Central and South America[16–18], US territories[19] and Europe.[20–23] CHIKV has now been identified in 94 countries worldwide.[6, 24] CHIKV infections reemerged in India after a gap of 32 years with an estimated 1.38 million people been infected by the end of 2006; the outbreak subsequently declined and by 2009 there were only a few thousand cases reported yearly.[25, 26]La Reunion Island, a French territory in the Indian Ocean, had the best-studied epidemic, and over one third of the inhabitants of the island were affected in the 2005–2006 outbreak.[27] In other outbreaks, such as in India and Malaysia much higher post-outbreak seropositivity rates were reported (62%-68%[28, 29] and 56%[30] respectively).

The first case of CHIKV infection in the Western Hemisphere was reported in 2013[18], and it has now rapidly spread to 44 countries in the Americas,[19, 31] including also US territories and the Caribbeans.[32, 33] In the US, in 2014 there was the first report of local- autochthonous CHIKV transmission in Florida.[34] Among 2,799 CHIKV cases reported to ArboNET in 2014 from US states, 12 cases were locally-transmitted (from Florida); while all the remaining cases were from returning travelers from endemic areas. In contrast, 99% of the 4,710 CHIKV cases reported from US territories were locally-transmitted. CHIKV infection became a nationally notifiable condition in 2015.[4] The number of CHIKV-infections reported in the US, declined after 2015 and in 2017 there were only 36 reported cases from the US, with no locally transmitted cases, and 30 cases from US territories where local transmission continues.[35]

The almost global distribution of CHIKV as well as the possibility for autochthonous transmission in the US make CHIKV infections a threat to global health and also to domestic health in the US. According to the CDC predictive model estimates for the US based on climate data, the potential range where the *Aedes aegypti* and *Aedes albopictus mosquitos* could potentially live, survive and reproduce in the US is quite extensive.[36–38]These mosquitos are capable of transmitting other arboviral infections as well, except for CHIKV.[39, 40]

Despite the almost global distribution of CHIKV infection, data for the impact of acute CHIKV infections during pregnancy are sparse and uncertainties remain on several important clinical questions. The best studied maternal-fetal cohort for CHIKV infections during gestation is from La Reunion CHIKV outbreak in the Indian Ocean in 2005–2006[27]. Prior reviews on this specific question were not exhaustive on their searches and focused only on very few studies [41, 42]. The maternal-fetal data from all available cohorts and for all important fetal and neonatal clinical outcomes has not been previously systematically evaluated. We set up to perform a systematic review and when meaningful, synthesize also the data by meta-analysis, to address the following questions: a) What is the overall risk of Mother-To-Child-Transmission (MTCT) from maternal CHIKV infections during gestation. b) What is the risk for antepartum fetal deaths (APFDs) from maternal CHIKV-infections during gestation. c) How often maternal CHIKV-infections during gestation lead to symptomatic neonatal disease and d) whether the reported risk-differences (for MTCT risk and risk of symptomatic disease) from maternal infections during the intrapartum period vs antepartum or peripartum period are consistent across diverse cohorts. Moreover, we wanted to record the spectrum of clinical manifestations reported in the scientific literature for neonatal CHIKV infections from maternal acute CHIKV-infections during gestation.

## Methods

We performed a systematic review to address the above questions and when meaningful, we also synthesized the data by meta-analysis. We searched PubMed and CINAHL databases (last search 3/2017) using the following terms: “chikungunya” and a pregnancy-related term that included any of the following terms: pregnan*, neonat*, perinat*, infant, mother, congenital, vertical transmission, miscarriage, abortion; limiting search results to human studies. Eligible for inclusion at the initial screening at title/abstract level were articles that studied CHIKV-infection in pregnant women, reported outcomes for the fetuses and/or newborns and had an English abstract. Potentially eligible articles were further screened in full text. The reference lists of key pertinent articles were also screened. Review article without original data were excluded. Article screening was done by two independent investigators (SNL and CC) and full texts of potentially eligible articles were also screened by a third investigator (DCI) and consensus was reached. Data extraction from the eligible articles was done by two independent investigators (SNL and CC) and confirmed by a third investigator (DCI).

### Eligibility criteria-outcomes

For the quantitative data synthesis, we included cohort studies, case series or case control studies that provided data on maternal CHIKV-infections during gestation and the CHIKV-infection status of their fetuses and/or newborns to allow for the calculation of the risk for: a) mother-to-child transmission (MTCT), b) antepartum fetal deaths (APFD), c) symptomatic neonatal disease, and d) neonatal deaths from maternal CHIKV-infections during gestation. We also calculated the overall combined fetal/neonatal disease impact of maternal CHIKV infection during gestation for a composite outcome of symptomatic neonatal disease plus the APFDs.

For the qualitative data synthesis, we considered studies of any study design, including case reports, that reported clinical manifestations of neonates exposed to maternal-CHIKV-infections during gestation. Reports of postnatally acquired CHIKV-infections from mosquito exposure were excluded.

For endpoints with ≥5 events in more than one study, we also synthesized data by random-effect- model meta-analyses.[43]

### Extracted data

From each eligible study for the quantitative data synthesis we extracted the following information: authors, year, locations, year of study, period of recent regional CHIKV-infection outbreak, duration of study, any possible overlap with prior published reports from the same cohort, number of pregnant women infected during gestation, number of neonatal infections from maternal infections during gestation, number of neonatal infections from intrapartum (-2 ds prior-to-delivery to +2 ds post-delivery), peripartum (-7 ds to -3 ds prior-to-delivery) and antepartum (>7 ds prior-to-delivery) maternal infections, number of antepartum fetal deaths, number of CHIKV-confirmed APFDs, number of symptomatic neonatal infections, number of symptomatic neonatal infections from intrapartum, peripartum and antepartum maternal infections, number of neonatal deaths, methods for ascertainment of maternal and neonatal CHIKV-infections. For the qualitative data synthesis, we extracted information on the clinical manifestations of neonatal infections documented to have occurred from suspected or confirmed maternal infections during gestation.

### Statistical analyses

Data were synthesized across cohorts by simple pooling. For each outcome of interest (MTCT-risk, APFD-risk, CHIKV-confirmed-APFD-risk, Symptomatic neonatal disease-risk; Neonatal death-risk) we calculated -for each cohort and across all analyzed cohorts- the pooled risk (and 95% confidence intervals thereof) among total CHIKV maternal infections during gestation (N of fetuses/neonates with the outcome of interest/ total N of CHIKV maternal infections during gestation). For the neonatal mortality outcome, we also calculated the risk of neonatal deaths among total neonatal infections.

As for the majority of the analyzed cohorts, data were reported only for symptomatic neonatal infections rather than for total neonatal infections (symptomatic plus asymptomatic), in our overall data synthesis for the MTCT-risk, we included also studies reporting only symptomatic-neonatal-disease-risk and considered that the MTCT-risk for those studies was at least equal to the risk for symptomatic neonatal disease. We also used random effect models (REMs)[43] for the calculation of the above risks to account for the between study variance. For outcomes with events <5 we used only simple pooling as REMs in such cases give unreliable results. We used the i^2^ test for the calculation of the between study heterogeneity. All proportion meta-analyses were done in STATASE 15.0 (Stata, College Station, TX, USA). When there were multiple publications from the same cohorts we considered in our overall data synthesis only the report with the maximum number of events for the outcome(s) of interest, per total number of analyzed maternal infections reported from the whole cohort.

In our systematic review, we followed the PRISMA (Preferred Reporting Items for Systematic reviews and Meta-Analyses) guidelines of reporting (S1 Table)_._ [44]

## Results

### Characteristics of included studies

**Fig 1:**
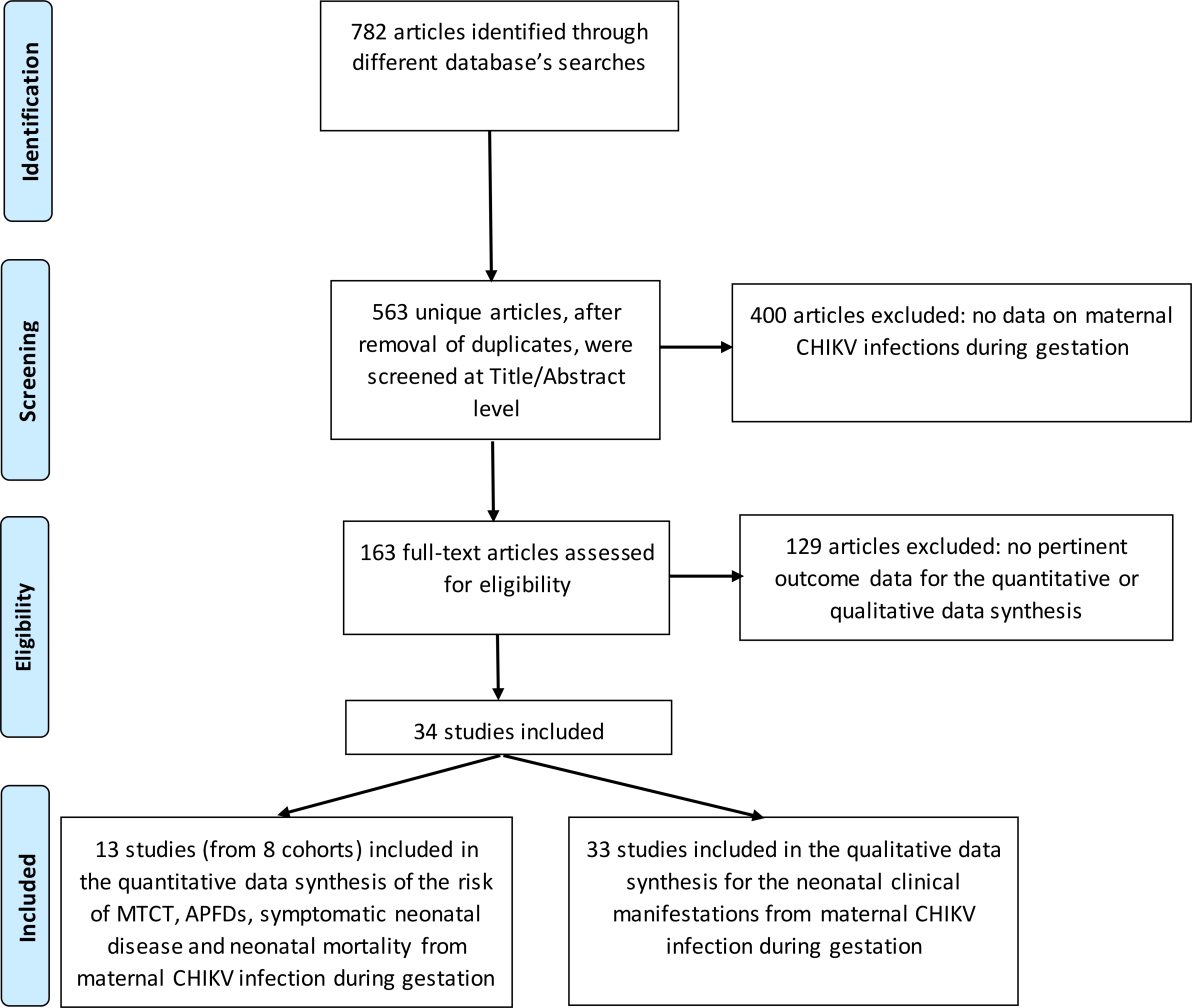
PRISMA flow diagram. (Abbreviations: APFD: Antepartum Fetal Deaths, CHIKV: Chikungunya Virus, MTCT: Mother-To-Child Transmission).

Of the 563 identified articles, 13 [27, 45–56] (from 8 cohorts) with pertinent data were included in the quantitative data synthesis (Fig 1).

These pertained to data from outbreaks in La Reunion Island (n = 7), Mayotte Island (n = 1), Sri Lanka (n = 1), Thailand (n = 2) and Latin America (n = 2) (S2 Table). Furthermore, 33 articles [27, 45–51, 53–55, 57–77] were included in the qualitative data synthesis of neonatal clinical manifestations from maternal infections during gestation. (Fig 1).

In the majority of articles in the quantitative data synthesis, maternal CHIKV-infections were ascertained by maternal serology (IgM and IgG) and /or blood RT-PCR and/or maternal symptoms typical of CHIKV-infections. Only in the recent outbreak from Santo Domingo the diagnosis of maternal CHIKV-infections was based only on clinical criteria (S2 Table).

### Risk of mother-to-child transmission

**Fig 2:**
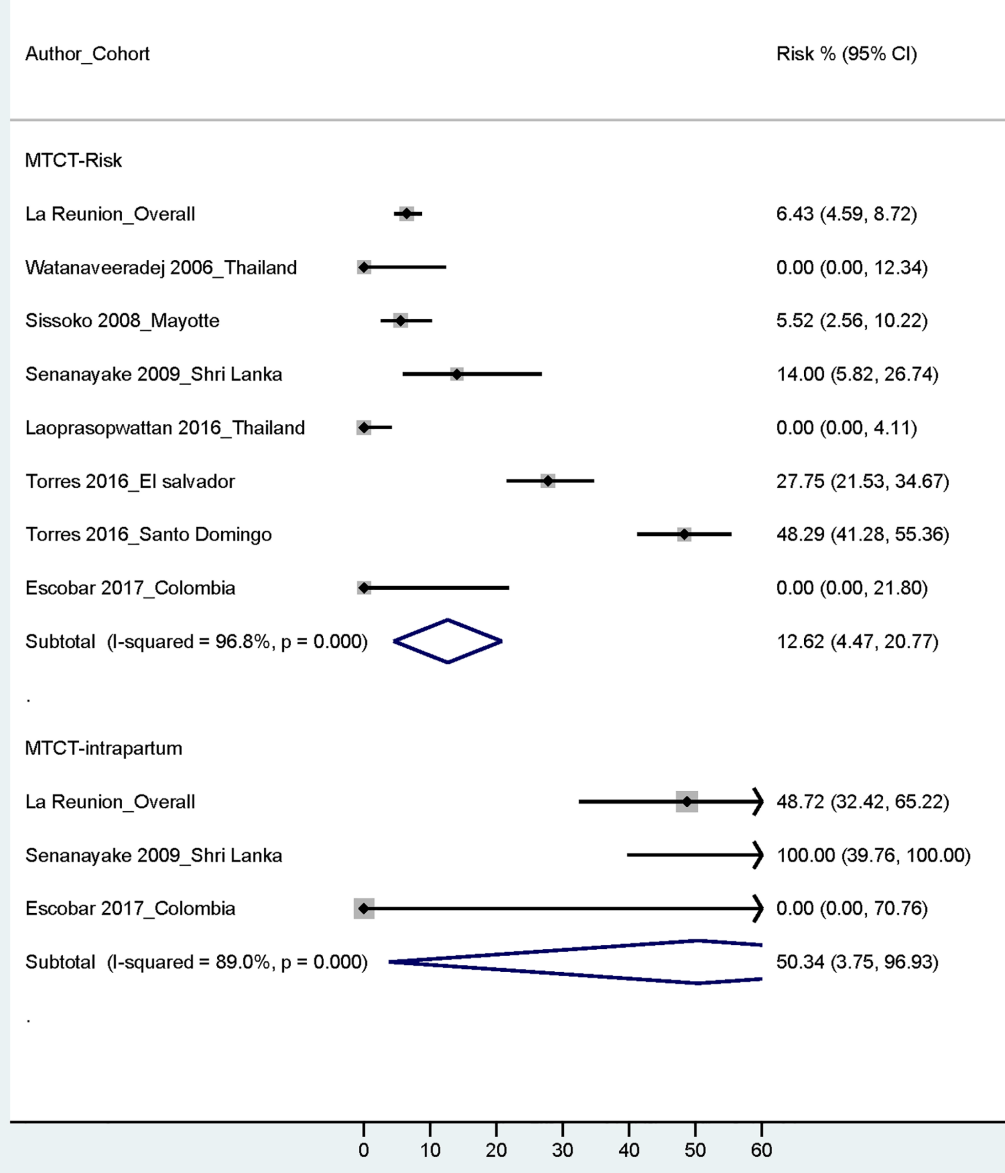
Mother-to-child-transmission (MTCT) risk: Overall and among intrapartum maternal infections. (For studies that did not report data on all (symptomatic and asymptomatic) neonatal infections, we extrapolated the data from the symptomatic neonatal disease cases [27, 51, 55]; and we considered that the MTCT-risk was at least the risk of symptomatic neonatal disease. Most cohorts that reported neonatal infections had reported only symptomatic cases [27, 51, 55] [S3 and S4 Tables]).

In most of the analyzed maternal/neonatal cohorts only symptomatic neonatal cases were reported among maternal CHIKV infections during gestation. By extrapolation also of these data [27, 51, 55], the overall pooled-MTCT-risk across cohorts was at least 15.5% (95% CIs: 13.57%-17.53%; 206/1331) (Table 1; S3 and S4 Tables) and the risk among maternal infections during the intrapartum period was at least 50.0% (95% CIs: 34.90%-65.10%; 23/46) vs 0% (0/712) among antepartum/peripartum maternal infections. The timing of maternal infections was analyzed only in three cohorts [27, 53, 56]; 5% of all analyzed maternal infections in these three cohorts occurred during the intrapartum period. Results by REM synthesis of data were similar (MTCT-overall risk: at least 12.6% [95% CIs 4.47%-20.77%]; MTCT-risk-intrapartum infections: at least 50.3% [3.75%-96.93%]). (Fig 2, Table 1)

**Table 1:** Fetal/Neonatal Risks from CHIKV maternal infections during gestation (Estimates by simple pooling and by REM).

Endpoint	Events (studies)	Maternal Infections		Risk (%; 95% CIs)
**MTCT-risk-overall**	206 (8)	1331	Simple Pooling	15.48% (13.57%-17.53%)
			By REM	12.62% (4.47%-20.77%)
MTCT-among antepartum/ peripartum maternal infections	0 (2)	712	Simple Pooling	0.00% (0%-0.52%)
			By REM^a^	NA
MTCT-among intrapartum maternal infections	23 (3)	46	Simple Pooling	50.00% (34.90%-65.10%)
			By REM	50.34% (3.75%-96.93%)
**APFD**	20 (5)	1203	Simple Pooling	1.66% (1.02%-2.56%)
			By REM^a^	NA
APFD-CHIKV-confirmed	3 (5)	1203	Simple Pooling	0.25% (0.05%-0.73%)
			By REM^a^	NA
**Symptomatic Neonatal Infections-overall**	203 (8)	1331	Simple Pooling	15.25% (13.36%-17.30%)
			By REM	11.92% (3.89%-19.95%)
Symptomatic Neonatal Infections-among Intrapartum Maternal infections	23 (3)	46	Simple Pooling	50.00% (34.90%-65.10%)
			By REM	50.34%(3.75%-96.93%)
Symptomatic Neonatal Infections-among antepartum/ peripartum maternal infections	0 (3)	758	Simple Pooling	0.00% (0%-0.49%)
			By REM^a^	NA
**Neonatal Deaths-among maternal Infections**	5 (3)	832	Simple Pooling	0.60% (0.20%-1.40%)
			By REM^a^	NA
**Neonatal Deaths-among neonatal Infections**	5 (3)	182	Simple Pooling	2.75% (0.90%-6.29%)
			By REM^a^	NA
**Combined Fetal/Neonatal Disease Impact,** from maternal CHIKV infection during gestation (MTCT and APFD)	226 (8)	1331	Simple pooling	16.98% (15.00%-19.11%)

^a^ For analyses with small number of events <5 in total or with <5 events for the majority of the included studies, we show only the data from simple pooling, as REM results are unreliable in those cases.

**Abbreviations**: APFD: Antepartum Fetal Deaths; CHIKV: chikungunya virus; CIs: confidence intervals; MTCT: mother to child transmission; NA: not applicable; REM: random effects model

### Fetal mortality

The pooled-risk of APFDs was 1.7% (95% CIs: 1.02%-2.56%; 20/1203) among maternal infections (Table 1; S3 and S4 Tables). APFDs occurred with maternal infections in all trimester, including during early gestation. Ascertainment of the CHIKV-infection status of APFDs was very rarely performed and was confirmed in only three cases from La Reunion outbreak, after maternal infections at 12.5 weeks, 15 weeks and 15.5 weeks of gestation respectively.[57] The pooled-risk of CHIKV-confirmed APFD cases was 0.3% (95% CIs: 0.05%-0.73%; 3/1203).

### Risk of symptomatic disease

**Fig 3:**
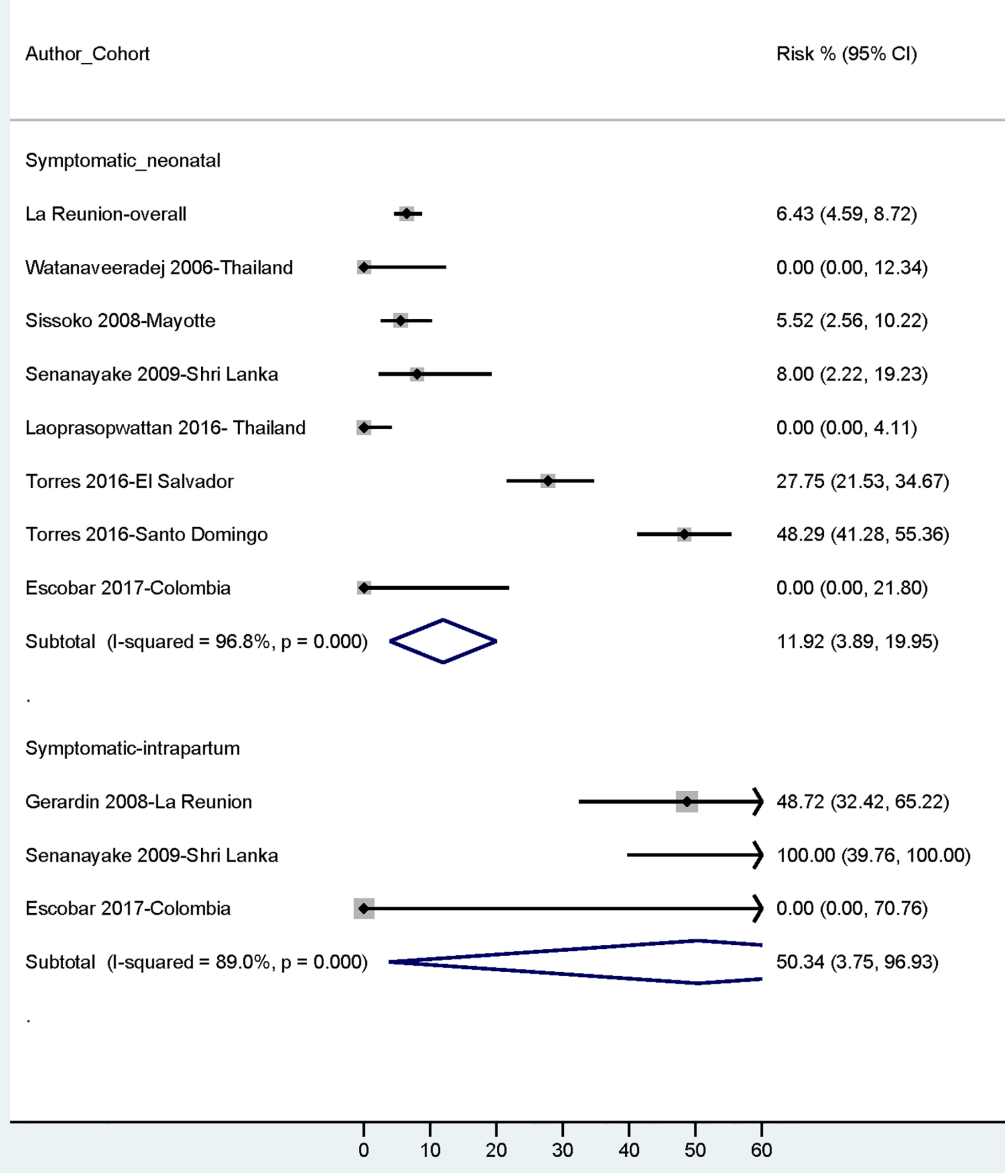
Risk of symptomatic neonatal disease: Overall and among intrapartum maternal infections.

Overall, the pooled-risk of symptomatic neonatal infections was 15.3% (95% CIs: 13.36%-17.30%; 203/1331) among maternal infections during gestation (Table 1; S3 and S4 Tables). However, this risk was 50.0% (95% CIs: 34.90%-65.10%; 23/46) among intrapartum maternal infections vs 0% (0/758) among antepartum/peripartum maternal infections. Only three maternal-fetal cohorts (La Reunion[27], ShriLanka [53] and Colombian cohort[56]) analyzed their data according to the timing of maternal infection and the reported risks for symptomatic neonatal disease from intrapartum paternal infections across these three cohorts were 48.7% (19/39) [27], 100% (4/4) [53] and 0% (0/3) [56] respectively. The reported cases of symptomatic neonatal disease were almost exclusively from intrapartum maternal infections. The majority of the cohorts did not provide information on the percentage of pregnant women with infections during the intrapartum period. Results by REM synthesis of data were similar (Symptomatic Neonatal disease-overall risk: 11.9% [95% CIs: 3.89%-19.95%]; Symptomatic Neonatal Diseases Risk-intrapartum infections: 50.3% [95% CIs: 3.75%-96.93%]) (Table 1; Fig 3).

Recording of long-term neurodevelopmental outcomes was very limited and was available only from La Reunion cohort, which showed neurodevelopmental delays at ~2 years of age in 50% of symptomatic neonatal infections (12 with CHIKV-encephalopathy and 22 with mild/moderate prostration) (S3 Table).

### Neonatal mortality

The pooled-risk for neonatal death was 0.6% (95% CIs: 0.20%-1.40%; 5/832) among all maternal infections and 2.8% (95% CIs: 0.90%-6.29%; 5/182) among neonatal infections. (Table 1)

### Combined fetal/neonatal disease impact from maternal CHIKV infections

The pooled combined disease impact to the fetus and newborn (MTCT and APFD) was 17.0% (95% CIs: 15.00%-19.11%; 226/1331) among maternal infections during gestation, considering both the neonatal infections and the APFDs. (Table 1). Limited data were available on the number of premature births from maternal CHIKV infections during gestation to allow for a meaningful data synthesis; however, the reported rates for premature births were low (3–8%).[47, 54, 56] (S5 Table)

### Qualitative data synthesis

#### Clinical spectrum of neonatal morbidity from maternal CHIKV infections during gestation

CHIKV-infected newborns, from maternal infections during gestation were either asymptomatic or presented with fever, irritability, hyperalgesia syndrome, diffuse limb edema, rashes and occasionally with severe disease including sepsis like picture and/or shock with multiple organ failure, disseminated intravascular coagulation, and meningoencephalitis with brain MRI abnormalities (S6 and S7 Tables).

Several types of rashes have been noted in such infected newborns including petechial rash, polymorphous rubella like rash, roseola like rash, bullous dermatitis or hyperpigmentation over mid-face, abdomen or extremities and acrocyanosis. Thrombocytopenia, leukopenia or leukocytosis, hypoalbuminemia and transaminitis with direct hyperbilirubinemia and prolongation of PTT have also been reported in symptomatic infants. The spectrum of clinical manifestations reported in neonates with CHIKV-infections, from maternal infections during gestation is listed in S6 and S7 Tables.

Newborns with symptomatic CHIKV-infection from maternal infection during gestation were asymptomatic at the time of birth and developed clinical symptomatology usually within 3–7 days of life.

## Discussion

In this systematic review of published data of the MTCT-risk and risk of symptomatic neonatal infection among maternal CHIKV infections during gestation, the number of identified cohorts, with pertinent data for such analyses, was very small compared to the number of recently reported CHIKV-infection outbreaks and the global distribution of CHIKV.[78] Most cohorts that reported neonatal infections had reported only symptomatic cases. By extrapolation of data also from symptomatic disease cases, the overall pooled-risk of MTCT across the 8 analyzed maternal-fetal cohorts was at least 15.5%. The risk of APFDs and CHIKV-confirmed APFDs was small (<2% and <0.5% respectively). APFDs and CHIKV-confirmed-APFDs occurred from maternal infections in all trimesters, including also during early gestation. Reporting of data on APFDs was limited across the analyzed cohorts and ascertainment of CHIKV infection status of APFD was reported for only 3 cases from La Reunion outbreak. The overall MTCT risk in our study might have been underestimated as the majority of the analyzed cohorts reported only symptomatic neonatal infections rather than on all neonatal infections. In resource poor settings, where most of the CHIKV outbreaks occurred, asymptomatic neonatal infections might have remained undiagnosed, leading to a possible selection bias among the cases studied. Selective follow-up of the sickest babies may also have skewed the results of several papers. Moreover, many had significant losses to follow-up or relied on neonatal disease incidence to estimate actual neonatal infection rates.

The overall pooled risk of symptomatic neonatal disease was 15.5% among maternal infections during gestation. However, the risk was 50.0% among intrapartum maternal infections vs 0% among antepartum/peripartum maternal infections. Data on the percentage of pregnant women infected during the intrapartum period were limited across the analyzed cohorts. Symptomatic neonatal disease occurred almost exclusively from intrapartum maternal infections. The pooled-risk for neonatal death was 0.6% among all maternal infections and 2.8% among neonatal infections. Long-term global neurodevelopmental delays have also been reported to occur in 50% of symptomatic neonatal infections during gestation, however this was based on a limited number of 33 such neonatal infections.[79]

In our qualitative data synthesis, we generated a compilation list of clinical manifestations reported in CHIKV-infected infants from maternal infections during gestation. Such infants presented with a wide spectrum of clinical manifestations ranging from asymptomatic to severely symptomatic. Symptomatic infected newborns, from maternal infections during gestation usually developed symptoms during their first week of life, but not at the time of birth. Commonly reported symptoms included fever, polyarthralgias, diffuse limb edema, irritability, poor feeding, painful syndrome and rashes; occasionally, also sepsis-like syndrome with multiple organ involvement, meningoencephalitis with brain MRI abnormalities and can also cause long term neurodevelopmental delays and devastating neurologic outcomes such as cerebral palsy.

There are anecdotal data for the use of interventions like tocolysis for the prolongation of transplacental transfer of protective maternal antibodies, for maternal infections acquired in the intrapartum period.[56] The average interval of ~6.3 +/- 1.4 days from the onset of maternal symptoms to delivery might have been enough time for the passive transfer of maternal antibodies to prevent MTCT and symptomatic disease in the newborn.[56] Tocolysis (as long as there are no obstetric contraindications), has been used also in other arboviral maternal infections, such as in dengue virus maternal infections to reduce the risk of vertical transmission [56] and in maternal varicella-zoster-virus infections during the peripartum period.[80] The safety and clinical effectiveness of tocolysis as a preventive measure in such intrapartum maternal infections requires additional systematic evaluation. Moreover, the number of pregnant women infected during the intrapartum period should be reported in maternal-fetal cohorts. In La Reunion,[27] the Sri Lanka[53] and the Colombia cohort[56] only 5% of maternal infections were acquired during the intrapartum period. The role of delivery via caesarean section (C/S) was analyzed only in La Reunion cohort [27] and appeared to have no influence on the MTCT risk. In La Reunion cohort[27] this observation may support the notion of transplacental transmission of CHIKV-infection from the mother to the fetus, rather than from exposure in the birth canal. The C/S rate among the 61 pregnant women in this cohort with peripartum/intrapartum infections was elevated compared to the baseline rate (43% vs 17%); the majority of those C/S was done due to fetal distress. [27] However, this was not seen in the Thailand cohort were the majority of the infants were born via vaginal delivery. [54]

For the interpretation of neonatal serologic test results, pediatricians and neonataologists should be aware that the absence of positive neonatal CHIKV IgG and IgM antibodies at birth in infants born to mothers with acute CHIKV-infections in the peripartum/intrapartum period does not exclude CHIKV neonatal infection. Infected newborns from such late maternal infections may have a delayed development of CHIKV IgG and IgM antibodies, within the first 3–4 weeks of life.[81] Serial serologic monitoring of these infants might be indicated as infected infants, particularly so symptomatic infants, might be at risk for poor long term neurodevelopmental outcomes.

Understanding the true impact of acute maternal CHIKV infections in the fetus and newborn requires systematic consideration also of fetal and neonatal mortality as well as ascertainment of long term neurodevelopmental outcomes in addition to the neonatal morbidity. Retrospectively extracted information about clinical signs and symptoms suggestive of acute maternal CHIKV infection during gestation likely underestimates the true incidence of maternal infections, due to recollection bias and non-capturing of mild or asymptomatic maternal infections. Moreover, standardized outcome collection and reporting across maternal-fetal cohorts is mandatory, to allow for prompt identification of the accurate risks to fetuses and newborns from maternal infections during gestation. Focus should be given during study design phase and outcome reporting for congenital/perinatal infections on all of the following: a) estimated time of maternal infection during gestation, with accurate reporting of the number of intrapartum maternal infections; b) consideration of APFDs in the overall combined fetal/neonatal disease impact from congenital CHIKV-infections; c) ascertainment of CHIKV-infections-status of APFDs; d) ascertainment of CHIKV infection status in all newborns exposed to suspected or confirmed maternal CHIKV-infections during gestation; and prompt documentation of losses to follow-up; e) serial screening of newborns exposed to late gestation maternal infections for the first month of life, even if they are seronegative at birth, given the likely delayed neonatal IgM and IgG production after late gestation maternal infections and f) ascertainment of long term neurodevelopmental outcomes for at least the symptomatic neonatal infections.

We observed significant variation in the reported rates of MTCT and symptomatic neonatal disease across cohorts. Referral selection bias and confounding by differences in the gestational age during maternal infections across cohorts might have explained the reported differences in the risks of symptomatic neonatal disease across cohorts, as symptomatic neonatal disease occurred almost exclusively from intrapartum maternal infections.[27, 45, 46] We were not able to make robust conclusions on the possible role of the implicated CHIKV strain in the observed variation in the MTCT-risks and risks of symptomatic disease across cohorts, given the limited number of cases. There is preliminary evidence that the different CHIKV-strains (Asian vs Central-East-South Africa [CESA] vs West Africa strain)[82, 83] might have different pathogenicity.[84] In outbreaks caused by the CESA CHIKV-strain[83], such as the La Reunion[50] and Mayotte[51] outbreaks the overall risk of symptomatic neonatal disease among all maternal infections was 6.26% (37/591) and 5.5% (9/163) respectively. In outbreaks where the Asian CHIKV-strains were implicated, the reported rates of symptomatic disease varied even more, with 0% MTCT rates from the Thailand cohorts[52, 54] and a small Colombian cohort[56]; versus 8% (4/50) for severely symptomatic neonatal disease from the Shri Lanka cohort,[53] 27.7% (53/191) from the El Salvador cohort[55] and 48% from the Santo Domingo cohort.[55] Nevertheless, there are recent data indicating that in South America CHIKV outbreaks, the African CESA CHIKV strains might also be implicated.[85, 86] Moreover, differences across cohorts were also noted in the reported neonatal case fatality rates, with 0% for La Reunion cohort [27] versus 5.1% for the Santo Domingo cohort.[55]

The number of neonatal CHIKV infections could be significantly underestimated using neonatal CHIKV IgG and IgM antibodies at birth. Ramful et al[49] showed that CHIKV infected infants (even symptomatic ones) from late-gestation maternal infections during the peripartum/intrapartum period can be seronegative at birth and might have delayed production of CHIKV antibodies; up to 3 weeks after birth for the development of IgMs and up to 4 weeks after birth for the development of positive IgGs. This is known to occur also in other congenital infections (e.g. congenital Toxoplasmosis[87]) when maternal infections occur very late in gestation and this might have underestimated the overall rate of mother-to-child transmission of CHIKV-infections in some of the analyzed reports.

Continued monitoring of the clinical implications of CHIKV infections during pregnancy is needed as CHIKV outbreaks can reemerge in regions where the virus had already previously circulating or emerge in new regions, where it had not been previously detected. Recently in 2017 a CHIKV outbreak was noted again in Italy, in the Anzio west-coast recreational region close to Rome [88–90], caused by an CESA strain. This strain was genetically slightly different from the strain implicated in the large 2007 outbreak in the Emilia-Romagna region in North-Eastern Italy.[23]

Furthermore, the potential benefit from tocolysis for intrapartum maternal infections is an intervention that needs systematic investigation, and if confirmed in larger scale studies to be effective, routine implementation in pregnant women with intrapartum maternal infection might have important public health implications. This might provide further support for the need for prenatal screening of pregnant women with suspected CHIKV infections during the peripartum/ intrapartum period. Future validation of the diagnostic performance of point-of-care tests for the serologic diagnosis of CHIKV maternal and/or neonatal infection and/or other arboviral infections is urgently needed. Moreover, preventive measures targeting avoidance of mosquito bites in pregnant women close to the expected time of delivery, might be cost-saving and effective strategies, given the high neonatal morbidity associated with intrapartum maternal infections. Furthermore, neonatologist, need to become aware that CHIKV-infected newborns from maternal infections late in gestation would need close clinical and laboratory monitoring of their hematologic parameters during their first week of life, even if they appear asymptomatic at birth, as symptomatic neonatal infections usually develop within 3–7 days after birth. Moreover, transplacentally transferred CHIKV-IgG antibodies on average disappear by 8 months of age in uninfected neonates.[49] However, the time to neonatal seroconversion is inversely related to the time of maternal infection during gestation; with >75% of non-infected neonates being still IgG positive by 12 months of age if maternal infection was in the first trimester vs 30% and <1% if maternal infection was in the second and third trimester respectively.[49] Moreover, it may take as long as 24 months for complete neonatal seroconversion to IgG negative status among uninfected neonates.[49]

Some study limitations should be acknowledged: *First*, in all analyzed cohorts (even those with serologic and/or molecular confirmation of CHIKV maternal infections), it was the presence of maternal symptoms the first indicator that led to subsequent testing of those women for CHIKV-infections. This may have led to overestimation of the MTCT risk and the risk of symptomatic neonatal disease from maternal CHIKV infections, if symptomatic maternal infections have an incrementally higher risk of MTCT, independently of the time of maternal infection. The effect of asymptomatic CHIKV maternal infections during pregnancy remains largely unknown. The majority of the CHIKV infections were originally thought to be symptomatic [91], nevertheless, recent reports indicate that the number of asymptomatic CHIKV infections might have been higher than it was originally thought. A report from the 2008 Thailand outbreak[92] showed that 50% of all cases were asymptomatic and a more recent report from that region showed that 87.5% of pregnant women infected during gestation were asymptomatic.[54] Nevertheless, conflicting results from the same region were also reported, with only 9% reported asymptomatic cases.[93] Additional surveillance studies from recent CHIKV outbreaks in Nicaragua also showed that 65% of cases were asymptomatic, with a symptomatic to asymptomatic ratio of 1:1.91.[94] Recollection bias might also explain some of the observed differences in the reported rates of symptomatic CHIKV-infections. *Second*, in the majority of the analyzed maternal/neonatal cohorts, only symptomatic neonatal infections were reported which might have underestimated the true MTCT-risk. *Third*, we could not identify published cohorts with an English abstract with pertinent data for our quantitative data synthesis from the majority of countries with recent CHIKV outbreaks; such as outbreaks in African[5] [12] and Asian countries[14, 95–97], including large outbreaks in India[98–104] after the 2005 reemergence of CHIKV in India, outbreaks in the Carribeans [32, 105, 106], Pacific Island[78] and Saudi Arabia.[107] Language-bias is also a possible reason for this phenomenon. Moreover, the published cohort studies with pertinent data from the outbreak in Central and South America since 2013 were very limited[55, 56] compared to the scale of CHIKV transmission across more than 45 countries throughout the Americas and with >1.7 million suspected CHIKV-infection cases reported to the Pan American Health Organization (PAHO).[78] For the majority of these outbreaks, only isolated case reports and small case series were identified, which we included in the qualitative data synthesis on the list of reported clinical manifestations from neonatal CHIKV- infections from maternal infections during gestation. Language-bias is also a possible reason for this phenomenon. It is possible that publications from several of these developing countries where such outbreaks occur are published only in grey literature [108] or in local non-English journals and indexed only in local journal databases, but not in PubMed. Moreover, the lack of financial resources and availability of accurate diagnostic tests[109] in several of the settings where such CHIKV outbreaks occur, contribute to the phenomenon of over-estimation of the true incidence and severity of the disease.

In conclusion, CHIKV is an emerging arbovirus with a global distribution that can cause significant morbidity and also death in infected fetuses and newborns after maternal infections during gestation. Neonatal morbidity likely occurs predominantly from intrapartum maternal infections. Improvement is needed in the reporting of clinical important outcomes for congenitally/perinatally acquired fetal and neonatal infections. Data should be collected and reported in a standardized way across maternal-fetal cohorts for all clinically important endpoints to allow for informative meta-analyses and individual patient-level meta-analyses in this field of congenital infections. With increasing climate instability and human migration, additional CKIKV outbreaks may be expected and non-immune pregnant women in developing as well as developed countries are at risk. Additional systematic studies of the impact of the CHIKV maternal infections during gestation to the fetuses and newborns are needed.

## Supporting information

S1 Table PRISMA checklist. (DOCX)

S2 Table Characteristics of included cohorts in quantitative data synthesis. (DOCX)

S3 Table Risks from Maternal CHIKV-infections during gestation (MTCT-risk, APFD-risk, APFD-CHIKV-confirmed-risk, Symptomatic disease-risk; Neonatal death-risk). (DOCX)

S4 Table Data in analyses. (DOCX)

S5 Table Clinical manifestations in neonatal CHIKV-infections from maternal infections during gestation (listed in alphabetical order). (DOCX)

S6 Table Clinical manifestations of congenital/neonatal CHIKV Infections, from maternal infections during gestation (per study). (DOCX)

S7 Table Obstetric complications and maternal clinical manifestations from maternal CHIKV infections during gestation (in the analyzed cohort). (DOCX)

## References

[1] Dotters-Katz SK, Grace MR, Strauss RA, Chescheir N, Kuller JA. Chikungunya Fever Obstetric Considerations on an Emerging Virus. Obstet Gynecol Surv. 2015;70(7):453–7. 10.1097/OGX.0000000000000184 PubMed PMID: WOS:000357737800016. 26185916

[2] Doughty CT, Yawetz S, Lyons J. Emerging Causes of Arbovirus Encephalitis in North America: Powassan, Chikungunya, and Zika Viruses. Curr Neurol Neurosci. 2017;17(2). doi: ARTN 12 10.1007/s11910-017-0724-3. PubMed PMID: WOS:000397031600008. 10.1007/s11910-017-0724-3 28229397

[3] Patterson J, Sammon M, Garg M. Dengue, Zika and Chikungunya: Emerging Arboviruses in the New World. West J Emerg Med. 2016;17(6):671–9. 10.5811/westjem.2016.9.30904 PubMed PMID: WOS:000394363800001. 27833670 PMC5102589

[4] CDC. Centers for Disease Control and Prevention: Chikungunya virus. https://www.cdc.gov/chikungunya/geo/united-states-2016.html.

[5] Olajiga OM, Adesoye OE, Emilolorun AP, Adeyemi AJ, Adeyefa EO, Aderibigbe IA, et al Chikungunya Virus Seroprevalence and Associated Factors among Hospital Attendees in Two States of Southwest Nigeria: A Preliminary Assessment. Immunol Invest. 2017;46(6):552–65. 10.1080/08820139.2017.1319383 PubMed PMID: WOS:000406546400003. 28742401

[6] Enserink M. Infectious diseases—Chikungunya: No longer a Third World disease. Science. 2007;318(5858):1860–1. 10.1126/science.318.5858.1860 PubMed PMID: WOS:000251786600017. 18096785

[7] Silva LA, Dermody TS. Chikungunya virus: epidemiology, replication, disease mechanisms, and prospective intervention strategies. J Clin Invest. 2017;127(3):737–49. 10.1172/JCI84417 PubMed PMID: WOS:000396658300002. 28248203 PMC5330729

[8] Lumsden WH. An epidemic of virus disease in Southern Province, Tanganyika Territory, in 1952–53. II. General description and epidemiology. Trans R Soc Trop Med Hyg. 1955;49(1):33–57. . 14373835 10.1016/0035-9203(55)90081-x

[9] Peper SM, Monson BJ, Van Schooneveld T, Smith CJ. That Which Bends Up: A Case Report and Literature Review of Chikungunya Virus. J Gen Intern Med. 2016;31(5):576–81. 10.1007/s11606-015-3459-3 PubMed PMID: WOS:000374461400028. 26194641 PMC4835393

[10] Borgherini G, Poubeau P, Staikowsky F, Lory M, Le Moullec N, Becquart JP, et al Outbreak of Chikungunya on Reunion Island: Early clinical and laboratory features in 157 adult patients. Clin Infect Dis. 2007;44(11):1401–7. 10.1086/517537 PubMed PMID: WOS:000246198800001. 17479933

[11] Hortion J, Vu DM, Grossi-Soyster EN, Okuta V, Jembe Z, Maina P, et al Chikungunya Virus Infection Is Causing Acute Febrile Illness among Children in Kenya. Am J Trop Med Hyg. 2017;95(5):223–. PubMed PMID: WOS:000412851502230.

[12] LaBeaud AD, Banda T, Brichard J, Muchiri EM, Mungai PL, Mutuku FM, et al High Rates of O'Nyong Nyong and Chikungunya Virus Transmission in Coastal Kenya. Plos Neglect Trop D. 2015;9(2). doi: UNSP e0003436 10.1371/journal.pntd.0003436 PubMed PMID: WOS:000350992500014. PMC4319898 25658762

[13] Afreen N, Deeba F, Khan WH, Haider SH, Kazim SN, Ishrat R, et al Molecular characterization of dengue and chikungunya virus strains circulating in New Delhi, India. Microbiol Immunol. 2014;58(12):688–96. 10.1111/1348-0421.12209 PubMed PMID: WOS:000346767000004. 25346397

[14] Khatun S, Chakraborty A, Rahman M, Banu NN, Rahman MM, Hasan SMM, et al An Outbreak of Chikungunya in Rural Bangladesh, 2011. Plos Neglect Trop D. 2015;9(7). doi: ARTN e0003907 10.1371/journal.pntd.0003907 PubMed PMID: WOS:000359079700022. PMC4498910 26161995

[15] Tan KK, Sy AKD, Tandoc AO, Khoo JJ, Sulaiman S, Chang LY, et al Independent Emergence of the Cosmopolitan Asian Chikungunya Virus, Philippines 2012. Sci Rep-Uk. 2015;5. doi: ARTN 12279 10.1038/srep12279 PubMed PMID: WOS:000358358000001. 26201250 PMC5378875

[16] Crosby L, Perreau C, Madeux B, Cossic J, Armand C, Herrmann-Storke C, et al Severe manifestations of chikungunya virus in critically ill patients during the 2013–2014 Caribbean outbreak. Int J Infect Dis. 2016;48:78–80. 10.1016/j.ijid.2016.05.010 PubMed PMID: WOS:000378046700019. 27208636

[17] Friedrich MJ. Chikungunya Virus Spreading in the Caribbean and South America. Jama-J Am Med Assoc. 2014;312(3):222–. PubMed PMID: WOS:000338940300008.

[18] Hamer DH, Chen LH. Chikungunya: Establishing a New Home in the Western Hemisphere. Ann Intern Med. 2014;161(11):827–U118. 10.7326/M14-1958 PubMed PMID: WOS:000347247200012. 25244354

[19] CDC. Centers for Disease Control and Prevention: Chikungunya virus: Geographic distribution. https://wwwcdcgov/chikungunya/geo/indexhtml. accessed 12/6/2017.

[20] Delisle E, Rousseau C, Broche B, Leparc-Goffart I, L'Ambert G, Cochet A, et al Chikungunya outbreak in Montpellier, France, September to October 2014. Eurosurveillance. 2015;20(17):8–13. PubMed PMID: WOS:000354464100002. 10.2807/1560-7917.es2015.20.17.21108 25955774

[21] Gould EA, Gallian P, de Lamballerie X, Charrel RN. First cases of autochthonous dengue fever and chikungunya fever in France: from bad dream to reality! Clin Microbiol Infec. 2010;16(12):1702–4. 10.1111/j.1469-0691.2010.03386.x PubMed PMID: WOS:000284170400002. 21040155

[22] Tomasello D, Schlagenhauf P. Chikungunya and dengue autochthonous cases in Europe, 2007–2012. Travel Med Infect Di. 2013;11(5):274–84. 10.1016/j.tmaid.2013.07.006 PubMed PMID: WOS:000326433300003. 23962447

[23] Rezza G, Nicoletti L, Angelini R, Romi R, Finarelli AC, Panning M, et al Infection with chikungunya virus in Italy: an outbreak in a temperate region. Lancet. 2007;370(9602):1840–6. 10.1016/S0140-6736(07)61779-6 PubMed PMID: WOS:000251588700030. 18061059

[24] Nsoesie EO, Kraemer MU, Golding N, Pigott DM, Brady OJ, Moyes CL, et al Global distribution and environmental suitability for chikungunya virus, 1952 to 2015. Eurosurveillance. 2016;21(20):7–18. doi: Artn 30234 10.2807/1560-7917.Es.2016.21.20.30234 PubMed PMID: WOS:000376390800002. 27239817 PMC4902126

[25] Directorate of National Vector Borne Disease Control Programme. Status report on Dengue and Chikungunya as of 31.12.09. Dengue and Chikungunya virus update 2010. http://nvbdcpgovin/Doc/Den_Chik_Dec09pdf (2010) Accessed December 17, 2017.

[26] Kumar CVMN Gopal DVRS. Reemergence of Chikungunya virus in Indian Subcontinent. Indian J Virol. 2010;21(1):8–17. 10.1007/s13337-010-0012-1 PubMed PMID: WOS:000284998300004. 23637474 PMC3550762

[27] Gerardin P, Barau G, Michault A, Bintner M, Randrianaivo H, Choker G, et al Multidisciplinary prospective study of mother-to-child chikungunya virus infections on the Island of La Reunion. Plos Med. 2008;5(3):413–23. doi: ARTN 060 10.1371/journal.pmed.0050060. PubMed PMID: WOS:000254928900016. 10.1371/journal.pmed.0050060 PMC2267812 18351797

[28] Manimunda SP, Sugunan AP, Rai SK, Vijayachari P, Shriram AN, Sharma S, et al Short Report: Outbreak of Chikungunya Fever, Dakshina Kannada District, South India, 2008. Am J Trop Med Hyg. 2010;83(4):751–4. 10.4269/ajtmh.2010.09-0433 PubMed PMID: WOS:000282862500006. 20889860 PMC2946737

[29] Kumar NP, Suresh A, Vanamail P, Sabesan S, Krishnamoorthy KG, Mathew J, et al Chikungunya virus outbreak in Kerala, India, 2007: a seroprevalence study. Mem I Oswaldo Cruz. 2011;106(8):912–6. PubMed PMID: WOS:000299068800003. 10.1590/s0074-02762011000800003 22241110

[30] Ayu SM, Lai LR, Chan YF, Hatim A, Hairi NN, Ayob A, et al Seroprevalence Survey of Chikungunya Virus in Bagan Panchor, Malaysia. Am J Trop Med Hyg. 2010;83(6):1245–8. 10.4269/ajtmh.2010.10-0279 PubMed PMID: WOS:000285534900014. 21118929 PMC2990039

[31] Yactayo S, Staples JE, Millot V, Cibrelus L, Ramon-Pardo P. Epidemiology of Chikungunya in the Americas. J Infect Dis. 2016;214:S441–S5. 10.1093/infdis/jiw390 PubMed PMID: WOS:000413815700004. 27920170 PMC5137246

[32] LaBeaud AD, Noel T, Jungkind D, Yearwood K, Fields P, Widjaja S, et al Chikungunya Fever in the Caribbean: Clinical Findings from Grenada. Am J Trop Med Hyg. 2015;93(4):322–. PubMed PMID: WOS:000412844103052.

[33] Cauchemez S, Ledrans M, Poletto C, Quenel P, de Valk H, Colizza V, et al Local and regional spread of chikungunya fever in the Americas. Eurosurveillance. 2014;19(28):15–23. PubMed PMID: WOS:000339320800004. 25060573 10.2807/1560-7917.es2014.19.28.20854 PMC4340072

[34] CDC. Centers for Disease Control and Prevention: Chikungunya virus: 2014 final data for the United States. https://wwwcdcgov/chikungunya/geo/united-states-2014html. accessed 12/6/2017.

[35] CDC. Centers for Disease Control and Prevention: Chikungunya virus: 2017 final data for the United States. https://wwwcdcgov/chikungunya/geo/united-states-2017html. accessed 12/6/2017.

[36] Armstrong PM, Andreadis TG, Shepard JJ, Thomas MC. Northern range expansion of the Asian tiger mosquito (Aedes albopictus): Analysis of mosquito data from Connecticut, USA. PLoS Negl Trop Dis. 2017;11(5):e0005623 10.1371/journal.pntd.0005623 ; PubMed Central PMCID: PMCPMC5451134. 28545111 PMC5451134

[37] Hopperstad KA, Reiskind MH. Recent Changes in the Local Distribution of Aedes aegypti (Diptera: Culicidae) in South Florida, USA. J Med Entomol. 2016;53(4):836–42. 10.1093/jme/tjw050 . 27113103

[38] Porse CC, Kramer V, Yoshimizu MH, Metzger M, Hu R, Padgett K, et al Public Health Response to Aedes aegypti and Ae. albopictus Mosquitoes Invading California, USA. Emerg Infect Dis. 2015;21(10):1827–9. 10.3201/3210.150494 ; PubMed Central PMCID: PMCPMC4593441. 26401891 PMC4593441

[39] Cevallos V, Ponce P, Waggoner JJ, Pinsky BA, Coloma J, Quiroga C, et al Zika and Chikungunya virus detection in naturally infected Aedes aegypti in Ecuador. Acta Trop. 2018;177:74–80. 10.1016/j.actatropica.2017.09.029 PubMed PMID: WOS:000415779400011. 28982578

[40] Ruckert C, Weger-Lucarelli J, Fauver JR, Garcia-Luna SM, Ebel GD. The Effect of Co-Infection with Dengue, Chikungunya and Zika Virus on Vector Competence of Aedes Mosquitoes. Am J Trop Med Hyg. 2017;95(5):191–. PubMed PMID: WOS:000412851502131.

[41] Panchaud A, Stojanov M, Ammerdorffer A, Vouga M, Baud D. Emerging Role of Zika Virus in Adverse Fetal and Neonatal Outcomes. Clin Microbiol Rev. 2016;29(3):659–94. 10.1128/CMR.00014-16 ; PubMed Central PMCID: PMCPMC4978612. 27281741 PMC4978612

[42] van Aalst M, Nelen CM, Goorhuis A, Stijnis C, Grobusch MP. Long-term sequelae of chikungunya virus disease: A systematic review. Travel Med Infect Dis. 2017;15:8–22. 10.1016/j.tmaid.2017.01.004 . 28163198

[43] DerSimonian R, Laird N. Meta-analysis in clinical trials. Control Clin Trials. 1986;7(3):177–88. . 3802833 10.1016/0197-2456(86)90046-2

[44] Moher D, Liberati A, Tetzlaff J, Altman DG, Group P. Preferred reporting items for systematic reviews and meta-analyses: the PRISMA statement. Plos Med. 2009;6(7):e1000097 10.1371/journal.pmed.1000097 ; PubMed Central PMCID: PMCPMC2707599. 19621072 PMC2707599

[45] Lenglet Y, Barau G, Robillard PY, Randrianaivo H, Michault A, Bouveret A, et al [Chikungunya infection in pregnancy: Evidence for intrauterine infection in pregnant women and vertical transmission in the parturient. Survey of the Reunion Island outbreak]. J Gynecol Obstet Biol Reprod (Paris). 2006;35(6):578–83. . 17003745 10.1016/s0368-2315(06)76447-x

[46] Robillard PY, Boumahni B, Gerardin P, Michault A, Fourmaintraux A, Schuffenecker I, et al Vertical maternal fetal transmission of the chikungunya virus—Ten cases among 84 pregnant women. Presse Med. 2006;35(5):785–8. 10.1016/S0755-4982(06)74690-5 PubMed PMID: WOS:000237918800011. 16710146

[47] Ramful D, Carbonnier M, Pasquet M, Bouhmani B, Ghazouani J, Noormahomed T, et al Mother-to-child transmission of Chikungunya virus infection. Pediatr Infect Dis J. 2007;26(9):811–5. 10.1097/INF.0b013e3180616d4f PubMed PMID: WOS:000249455800008. 17721376

[48] Fritel X, Rollot O, Gerardin P, Gauzere BA, Bideault J, Lagarde L, et al Chikungunya virus infection during pregnancy, Reunion, France, 2006. Emerg Infect Dis. 2010;16(3):418–25. 10.3201/eid1603.091403 ; PubMed Central PMCID: PMCPMC3322036. 20202416 PMC3322036

[49] Ramful D, Samperiz S, Fritel X, Michault A, Jaffar-Bandjee MC, Rollot O, et al Antibody kinetics in infants exposed to Chikungunya virus infection during pregnancy reveals absence of congenital infection. J Infect Dis. 2014;209(11):1726–30. 10.1093/infdis/jit814 . 24338351

[50] Gerardin P, Samperiz S, Ramful D, Boumahni B, Bintner M, Alessandri JL, et al Neurocognitive outcome of children exposed to perinatal mother-to-child Chikungunya virus infection: the CHIMERE cohort study on Reunion Island. PLoS Negl Trop Dis. 2014;8(7):e2996 10.1371/journal.pntd.0002996 ; PubMed Central PMCID: PMCPMC4102444. 25033077 PMC4102444

[51] Sissoko D, Malvy D, Giry C, Delmas G, Paquet C, Gabrie P, et al Outbreak of Chikungunya fever in Mayotte, Comoros archipelago, 2005–2006. T Roy Soc Trop Med H. 2008;102(8):780–6. 10.1016/j.trstmh.2008.02.018 PubMed PMID: WOS:000258201600008. 18400240

[52] Watanaveeradej V, Endy TP, Simasathien S, Kerdpanich A, Polprasert N, Aree C, et al Transplacental chikungunya virus antibody kinetics, Thailand. Emerg Infect Dis. 2006;12(11):1770–2. PubMed PMID: WOS:000241573900025. 10.3201/eid1211.050560 17283634 PMC3372326

[53] Senanayake MP SS, Vidanage KK, Gunassena S, Lamabadusurlya SP. Vertical transmission in Chikungunya infection. Cylon Med J. 2009;54(2):47–50. 10.4038/cmj.v54i2.865 19670548

[54] Laoprasopwattana K, Suntharasaj T, Petmanee P, Suddeaugrai O, Geater A. Chikungunya and dengue virus infections during pregnancy: seroprevalence, seroincidence and maternal-fetal transmission, southern Thailand, 2009–2010. Epidemiol Infect. 2016;144(2):381–8. 10.1017/S0950268815001065 PubMed PMID: WOS:000368638100020. 26113247

[55] Torres JR, Falleiros-Arlant LH, Duenas L, Pleitez-Navarrete J, Salgado DM, Brea-Del Castillo J. Congenital and perinatal complications of chikungunya fever: a Latin American experience. Int J Infect Dis. 2016;51:85–8. 10.1016/j.ijid.2016.09.009 PubMed PMID: WOS:000388326700020. 27619845

[56] Escobar M, Nieto AJ, Loaiza-Osorio S, Barona JS, Rosso F. Pregnant Women Hospitalized with Chikungunya Virus Infection, Colombia, 2015. Emerg Infect Dis. 2017;23(11):1777–83. 10.3201/eid2311.170480 PubMed PMID: WOS:000413109500002. 29047427 PMC5652420

[57] Touret Y, Randrianaivo H, Michault A, Schuffenecker I, Kauffmann E, Lenglet Y, et al Early maternal-fetal transmission of the Chikungunya virus. Presse Med. 2006;35(11):1656–8. 10.1016/S0755-4982(06)74874-6 PubMed PMID: WOS:000242164400010. 17086120

[58] Robin S, Rainful D, Le Seach F, Jaffar-Bandjee MC, Rigou G, Alessandri JL. Neurologic manifestations of pediatric chikungunya infection. J Child Neurol. 2008;23(9):1028–35. 10.1177/0883073808314151 PubMed PMID: WOS:000258841800007. 18287573

[59] Gerardin P, Couderc T, Randrianaivo H, Fritel X, Lecuit M. CHIKUNGUNYA VIRUS-ASSOCIATED ENCEPHALITIS: A COHORT STUDY ON LA REUNION ISLAND, 2005–2009 Response. Neurology. 2016;86(21):2025–6. PubMed PMID: WOS:000376959900023. 10.1212/WNL.0000000000002732 27217467

[60] Boumahni B, Kaplan C, Clabe A, Randrianaivo H, Lanza F. Maternal-fetal chikungunya infection associated with Bernard-Soulier syndrome. Arch Pediatrie. 2011;18(3):272–5. 10.1016/j.arcped.2010.12.002 PubMed PMID: WOS:000288186400006. 21269816

[61] Alvarado-Socarras JL, Ocampo-Gonzalez M, Vargas-Soler JA, Rodriguez-Morales AJ, Franco-Paredes C. Congenital and Neonatal Chikungunya in Colombia. J Pediatr Infect Dis. 2016;5(3):E17–E20. 10.1093/jpids/piw021 PubMed PMID: WOS:000386138100001. 27125272

[62] Bandeira AC, Campos GS, Sardi SI, Rocha VFD, Rocha GCM. Neonatal encephalitis due to Chikungunya vertical transmission: First report in Brazil. IDCases. 2016;5:57–9. 10.1016/j.idcr.2016.07.008 PubMed PMID: WOS:000399150800019. 27500084 PMC4971151

[63] Evans-Gilbert T. Case Report: Chikungunya and Neonatal Immunity: Fatal Vertically Transmitted Chikungunya Infection. Am J Trop Med Hyg. 2017;96(4):913–5. 10.4269/ajtmh.16-0491 PubMed PMID: WOS:000401763000027. 28167590 PMC5392641

[64] Karthiga V, Kommu PPK, Krishnan L. Perinatal chikungunya in twins. J Pediatr Neurosci. 2016;11(3):223–4. 10.4103/1817-1745.193369 PubMed PMID: WOS:000390115700012. 27857791 PMC5108125

[65] Khandelwal K, Aara N, Ghiya BC, Bumb RA, Satoskar AR. Centro-Facial Pigmentation in Asymptomatic Congenital Chikungunya Viral Infection. J Paediatr Child H. 2012;48(6):542–3. 10.1111/j.1440-1754.2012.02484.x PubMed PMID: WOS:000305186200021. 22583142

[66] Kumar N, Gupta V, Thomas N. Brownie-nose: Hyperpigmentation in Neonatal Chikungunya. Indian Pediatr. 2014;51(5):419–. PubMed PMID: WOS:000336049800023. 24953593

[67] Lyra PPR, Campos GS, Bandeira ID, Sardi SI, Costa LFD, Santos FR, et al Congenital Chikungunya Virus Infection after an Outbreak in Salvador, Bahia, Brazil. Ajp Rep. 2016;6(3):E299–E300. 10.1055/s-0036-1587323 PubMed PMID: WOS:000382531200008. 27555980 PMC4993616

[68] Passi GR, Khan YZ, Chitnis DS. Chikungunya infection in neonates. Indian Pediatr. 2008;45(3):240–2. PubMed PMID: WOS:000254357300016. 18367775

[69] Boumahni B, Bintner M. [Five-year outcome of mother-to-child transmission of chikungunya virus]. Med Trop (Mars). 2012;72 Spec No:94–6. . 22693938

[70] Pinzon-Redondo H, Paternina-Caicedo A, Barrios-Redondo K, Zarate-Vergara A, Tirado-Perez I, Fortich R, et al RISK FACTORS FOR SEVERITY OF CHIKUNGUNYA IN CHILDREN A Prospective Assessment. Pediatr Infect Dis J. 2016;35(6):702–4. 10.1097/INF.0000000000001135 PubMed PMID: WOS:000379343700024. 26986769

[71] Shenoy S, Pradeep GCM. Neurodevelopmental Outcome of Neonates with Vertically Transmitted Chikungunya Fever with Encephalopathy. Indian Pediatr. 2012;49(3):238–40. PubMed PMID: WOS:000304110800015. 22484743

[72] Shrivastava A, Beg MW, Gujrati C, Gopalan N, Rao PVL. Management of a Vertically Transmitted Neonatal Chikungunya Thrombocytopenia. Indian J Pediatr. 2011;78(8):1008–9. 10.1007/s12098-011-0371-7 PubMed PMID: WOS:000293143700015. 21328079

[73] Valamparampil JJ, Chirakkarot S, Letha S, Jayakumar C, Gopinathan KM. Clinical profile of Chikungunya in infants. Indian J Pediatr. 2009;76(2):151–5. 10.1007/s12098-009-0045-x PubMed PMID: WOS:000264631100003. 19330303

[74] Vasani R, Kanhere S, Chaudhari K, Phadke V, Mukherjee P, Gupta S, et al Congenital Chikungunya-A Cause of Neonatal Hyperpigmentation. Pediatr Dermatol. 2016;33(2):209–12. 10.1111/pde.12650 PubMed PMID: WOS:000373067800055. 26205895

[75] Villamil-Gomez W, Alba-Silvera L, Menco-Ramos A, Gonzalez-Vergara A, Molinares-Palacios T, Barrios-Corrales M, et al Congenital Chikungunya Virus Infection in Sincelejo, Colombia: A Case Series. J Trop Pediatrics. 2015;61(5):386–92. 10.1093/tropej/fmv051 PubMed PMID: WOS:000365384300010. 26246086

[76] Rodriguez-Nieves M, Garcia-Garcia I, Garcia-Fragoso L. Perinatally Acquired Chikungunya Infection: The Puerto Rico Experience. Pediatr Infect Dis J. 2016;35(10):1163 10.1097/INF.0000000000001261 27622689

[77] Gopakumar H, Ramachandran S. Congenital chikungunya. J Clin Neonatol. 2012;1(3):155–6. 10.4103/2249-4847.101704 PubMed Central PMCID: PMCPMC3762016. 24027715 PMC3762016

[78] CDC. Centers for Disease Control and Prevention: Chikungunya virus: Geographic distribution. https://wwwcdcgov/chikungunya/geo/indexhtml. accessed December 8, 2017.

[79] Gerardin P, Samperiz S, Ramful D, Boumahni B, Bintner M, Alessandri JL, et al Neurocognitive Outcome of Children Exposed to Perinatal Mother-to-Child Chikungunya Virus Infection: The CHIMERE Cohort Study on Reunion Island. Plos Neglect Trop D. 2014;8(7). doi: ARTN e2996 10.1371/journal.pntd.0002996 PubMed PMID: WOS:000340551500043. PMC4102444 25033077

[80] Paulman PM, McLellan R. Varicella during pregnancy: the timing of effective treatment. J Am Board Fam Pract. 1990;3(2):121–3. . 2333759

[81] Ramful D, Samperiz S, Fritel X, Michault A, Jaffar-Bandjee MC, Rollot O, et al Antibody Kinetics in Infants Exposed to Chikungunya Virus Infection During Pregnancy Reveals Absence of Congenital Infection. J Infect Dis. 2014;209(11):1726–30. 10.1093/infdis/jit814 PubMed PMID: WOS:000336485900008. 24338351

[82] Lanciotti RS, Lambert AJ. Phylogenetic Analysis of Chikungunya Virus Strains Circulating in the Western Hemisphere. Am J Trop Med Hyg. 2016;94(4):800–3. 10.4269/ajtmh.15-0375 PubMed PMID: WOS:000373242400017. 26856917 PMC4824221

[83] Bessaud M, Peyrefitte CN, Pastorino BAM, Tock F, Merle O, Colpart JJ, et al Chikungunya virus strains, Reunion Island outbreak. Emerg Infect Dis. 2006;12(10):1604–6. PubMed PMID: WOS:000240910200026. 10.3201/eid1210.060596 17176585 PMC3290959

[84] Teo TH, Her Z, Tan JJ, Lum FM, Lee WW, Chan YH, et al Caribbean and La Reunion Chikungunya Virus Isolates Differ in Their Capacity To Induce Proinflammatory Th1 and NK Cell Responses and Acute Joint Pathology. J Virol. 2015;89(15):7955–69. 10.1128/JVI.00909-15 ; PubMed Central PMCID: PMCPMC4505608. 25995257 PMC4505608

[85] Rodrigues Faria N, Lourenco J, Marques de Cerqueira E, Maia de Lima M, Pybus O, Carlos Junior Alcantara L. Epidemiology of Chikungunya Virus in Bahia, Brazil, 2014–2015. PLoS Curr. 2016;8 10.1371/currents.outbreaks.c97507e3e48efb946401755d468c28b2 ; PubMed Central PMCID: PMCPMC4747681. 27330849 PMC4747681

[86] Souza TM, Azeredo EL, Badolato-Correa J, Damasco PV, Santos C, Petitinga-Paiva F, et al First Report of the East-Central South African Genotype of Chikungunya Virus in Rio de Janeiro, Brazil. PLoS Curr. 2017;9 10.1371/currents.outbreaks.4200119978d62ccaa454599cd2735727 ; PubMed Central PMCID: PMCPMC5325710. 28286701 PMC5325710

[87] Gilbert RE, Thalib L, Tan HK, Paul M, Wallon M, Petersen E, et al Screening for congenital toxoplasmosis: accuracy of immunoglobulin M and immunoglobulin A tests after birth. J Med Screen. 2007;14(1):8–13. 10.1258/096914107780154440 . 17362565

[88] Venturi G, Di Luca M, Fortuna C, Remoli ME, Riccardo F, Severini F, et al Detection of a chikungunya outbreak in Central Italy, August to September 2017. Eurosurveillance. 2017;22(39):11–4. doi: Artn 17–00646 10.2807/1560-7917.Es.2017.22.39.17–00646 PubMed PMID: WOS:000412080700003. PMC5709953 29019306

[89] Marano G, Pupella S, Pati I, Masiello F, Franchini M, Vaglio S, et al Ten years since the last Chikungunya virus outbreak in Italy: history repeats itself. Blood Transfus-Italy. 2017;15(6):489–90. 10.2450/2017.0215–17 PubMed PMID: WOS:000413802600001. PMC5649955 29053100

[90] Manica M, Guzzetta G, Poletti P, Filipponi F, Solimini A, Caputo B, et al Transmission dynamics of the ongoing chikungunya outbreak in Central Italy: from coastal areas to the metropolitan city of Rome, summer 2017. Eurosurveillance. 2017;22(44):2–9. 10.2807/1560-7917.Es.2017.22.44.17–00685 PubMed PMID: WOS:000414415800001. PMC5710132 29113629

[91] Thiberville SD, Moyen N, Dupuis-Maguiraga L, Nougairede A, Gould EA, Roques P, et al Chikungunya fever: Epidemiology, clinical syndrome, pathogenesis and therapy. Antivir Res. 2013;99(3):345–70. 10.1016/j.antiviral.2013.06.009 PubMed PMID: WOS:000327108300019. 23811281 PMC7114207

[92] Nakkhara P, Chongsuvivatwong V, Thammapalo S. Risk factors for symptomatic and asymptomatic chikungunya infection. T Roy Soc Trop Med H. 2013;107(12):789–96. 10.1093/trstmh/trt083 PubMed PMID: WOS:000327461400007. 24052594

[93] Appassakij H, Khuntikij P, Kemapunmanus M, Wutthanarungsan R, Silpapojakul K. Viremic profiles in asymptomatic and symptomatic chikungunya fever: a blood transfusion threat? Transfusion. 2013;53(10):2567–74. 10.1111/j.1537-2995.2012.03960.x PubMed PMID: WOS:000325978000023. 23176378

[94] Bustos F, Kuan G, Sanchez N, et al. An index cluster study of Chikungunya in Nicaragua with spatial and risk factor analyses (Abstract). 67th Annual Meeting, ASTMH (American Society of Tropical Medicine and Hygiene), October 18-November 1, 2018; New Orleans, Louisiana, USA

[95] Kosasih H, de Mast Q, Widjaja S, Sudjana P, Antonjaya U, Ma'roef C, et al Evidence for Endemic Chikungunya Virus Infections in Bandung, Indonesia. Plos Neglect Trop D. 2013;7(10). doi: ARTN e2483 10.1371/journal.pntd.0002483 PubMed PMID: WOS:000330376500023. 24205417 PMC3812099

[96] AbuBakar S, Sam IC, Wong PF, MatRahim N, Hooi PS, Roslan N. Reemergence of endemic Chikungunya, Malaysia. Emerg Infect Dis. 2007;13(1):147–9. PubMed PMID: WOS:000243692500025. 10.3201/eid1301.060617 17370532 PMC2725805

[97] Wu D, Wu J, Zhang QL, Zhong HJ, Ke CW, Deng XL, et al Chikungunya Outbreak in Guangdong Province, China, 2010. Emerg Infect Dis. 2012;18(3):493–5. 10.3201/eid1803.110034 PubMed PMID: WOS:000301024000020. 22377135 PMC3309566

[98] Mukherjee S, Dutta SK, Sengupta S, Tripathi A. Evidence of dengue and chikungunya virus co-infection and circulation of multiple dengue serotypes in a recent Indian outbreak. Eur J Clin Microbiol. 2017;36(11):2273–9. 10.1007/s10096-017-3061-1 PubMed PMID: WOS:000413620200035. 28756561

[99] Kawle AP, Nayak AR, Bhullar SS, Borkar SR, Patankar SD, Daginawala HF, et al Seroprevalence and clinical manifestations of chikungunya virus infection in rural areas of Chandrapur, Maharashtra, India. J Vector Dis. 2017;54(1):35–43. PubMed PMID: WOS:000399210000005. 28352044

[100] Jain J, Nayak K, Tanwar N, Gaind R, Gupta B, Shastri JS, et al Clinical, Serological, and Virological Analysis of 572 Chikungunya Patients From 2010 to 2013 in India. Clin Infect Dis. 2017;65(1):133–40. 10.1093/cid/cix283 PubMed PMID: WOS:000403459300023. 28379375

[101] Raghavendhar BS, Ray P, Ratagiri VH, Sharma B, Kabra SK, Lodha R. Evaluation of chikungunya virus infection in children from India during 2009–2010: A cross sectional observational study. J Med Virol. 2016;88(6):923–30. 10.1002/jmv.24433 PubMed PMID: WOS:000373627000001. 26581026

[102] Mudurangaplar B, Peerapur BV. Molecular Characterisation of Clinical Isolates of Chikungunya Virus: A Study from Tertiary Care Hospitals in Southern India. J Clin Diagn Res. 2016;10(3):Dc14–Dc7. 10.7860/JCDR/2016/18370.7509 PubMed PMID: WOS:000397847900026. 27134872 PMC4843258

[103] Kumar CVMN, Sivaprasad Y, Gopal DVRS. Genetic diversity of 2006–2009 Chikungunya virus outbreaks in Andhra Pradesh, India, reveals complete absence of E1:A226V mutation. Acta Virol. 2016;60(1):114–7. 10.4149/av_2016_01_114 PubMed PMID: WOS:000384894300016. 26982477

[104] Chattopadhyay S, Mukherjee R, Nandi A, Bhattacharya N. Chikungunya virus infection in West Bengal, India. Indian J Med Microbi. 2016;34(2):213–5. 10.4103/0255-0857.176839 PubMed PMID: WOS:000378536300017. 27080776

[105] Lane KR, Bennett SN. The 2013 Chikungunya Viral Outbreak in Grenada: A Phylogenetic Analysis of Introduction and Spread. Integr Comp Biol. 2017;57:E93–E. PubMed PMID: WOS:000398668700371.

[106] Macpherson C, Noel T, Fields P, Jungkind D, Yearwood K, Simmons M, et al Clinical and Serological Insights from the Asian Lineage Chikungunya Outbreak in Grenada, 2014: An Observational Study. Am J Trop Med Hyg. 2016;95(4):890–3. 10.4269/ajtmh.16-0122 PubMed PMID: WOS:000400206500034. 27527629 PMC5062795

[107] Hussain R, Alomar I, Memish ZA. Chikungunya virus: emergence of an arthritic arbovirus in Jeddah, Saudi Arabia. E Mediterr Health J. 2013;19(5):506–8. PubMed PMID: WOS:000322800700018. 24617133

[108] Hartling L, Featherstone R, Nuspl M, Shave K, Dryden DM, Vandermeer B. Grey literature in systematic reviews: a cross-sectional study of the contribution of non-English reports, unpublished studies and dissertations to the results of meta-analyses in child-relevant reviews. BMC Med Res Methodol. 2017;17(1):64 10.1186/s12874-017-0347-z PubMed Central PMCID: PMCPMC5395863. 28420349 PMC5395863

[109] McNerney R. Diagnostics for Developing Countries. Diagnostics (Basel). 2015;5(2):200–9. 10.3390/diagnostics5020200 ; PubMed Central PMCID: PMCPMC4665590. 26854149 PMC4665590

