# Supplementary material for: Mother-to-child transmission of Chikungunya virus: A systematic review and meta-analysis
Source: PLoS Negl Trop Dis. 2018 Jun 13;12(6):e0006510. doi: 10.1371/journal.pntd.0006510 (PMC6075784; doi:10.1371/journal.pntd.0006510)
Supplement: S2 Table — (DOCX) [file pntd.0006510.s002.docx]

|  | **S2 Table:** Characteristics of Included Cohorts in Quantitative Data Synthesis[1-13] | | | | | |
| --- | --- | --- | --- | --- | --- | --- |
| **ID** | **Cohort/Location/ Outbreak or Surveillance period and type of cohort** | **Author (year; period)** | **N of infected pregnant women (with ascertainment of CHIKV Fetal/Neonatal Infection status)** | **N of near term infection** | **Definitions of Maternal infection** | **Definitions of neonatal infections** |
| 1 | La Reunion, 2005-2006 outbreak | Lenglet 2006; 6/2005-2/2006 (overlap with some of Gerardin’ s 2008 data for this period) | N=151 maternal infections (N=116 non-clinically viremic at the time of delivery; 33 clinically viremic at the time of delivery) | N=33 clinically viremic mothers at the time of delivery | Symptomatic maternal infections or Mothers with APFD with Serologic IgM and/or RT-PCR | Serologic IgM and/or RT-PCR |
| 1 | La Reunion, 2005-2006 outbreak; Maternal and neonatal cohort | Robillard 2005; 6/2005-1/2006 (Overlap with some of Gerardin’s 2008 data for the same period) | N=84 symptomatic maternal infections | N=10 maternal infections with clinical viremia during the intrapartum period (the remaining 74 maternal infections distant from delivery) | Serologic (IgM) | Serologic diagnosis and/or RT_PCR only for the symptomatic newborns |
| 1 | La Reunion 2005-2006 outbreak; APFD cases | Touret 2006; | NR in this report | NR for all women in this publication  Data were reported only for the mothers of the APFD cases | Serology and RT-PCR (these maternal infections were confirmed by positive IgM, RT-PCRs were negative; AF RT-PCR was positive) | Amniotic fluid PCR |
| 1 | La Reunion, 2005-2006 outbreak; Neonatal cohort | Ramful 2007, 3/2005-4/2006 (overlap with some of Gerardin’s 2008 data for the same period) | NR in this report  Data reported only on 38 mothers of these 38 symptomatic newborn infants | N=36 c-p CHIKV infections/38 infected mothers were symptomatic in the perinatal period from -4ds PTD to +1 day post-delivery and 18 of those were clinically symptomatic at the time of delivery (2/38 infected mothers were asymptomatic) | Serology (IgM) or RT-PCR (26 mothers were tested with either IgM or RT-PCR) | Serology (IgM) or RT-PCR (24 neonates tested by RT-PCR and 30 by serology) |
| 1 | La Reunion; 2005-2006 outbreak; Maternal and neonatal cohort | Geradin 2008 (22-month period 6/2005-12/2006) | N~~=~~ 739   - 678 antepartum maternal infections >7 ds PTD; - 22 peripartum maternal infections -7 ds to -3 ds PTD - 39 intrapartum maternal infections -2ds to +2 ds) | N=61   - 39 intrapartum maternal infections -2 ds PTD to +2 ds post-delivery and - 22 peri-partum maternal infections -7 ds to -3 ds PTD~~)~~ | Clinical symptoms and positive IgM OR RT-PCR | Positive neonatal IgM at day of life 0, 7 and 15 OR positive RT-PCR at less than 7 days of life and symptoms had occurred during the first week of life in the absence of history of mosquito bite |
| 1 | La Reunion, 2005-2006 outbreak; Maternal and neonatal cohort | Fritel 2010, covering the (8 month-period from 4/2006-11/2006 with possible overlap with the data from Gerardin 2008 for this period) | N=658 maternal infections | 4 had symptoms within < 7 days from delivery | Clinical symptoms during pregnancy AND positive serology (IgM) or positive RT-PCR; (46 seropositive asymptomatic women were excluded, because the timing of infections was unknown); 21% were hospitalized. Maternal symptoms began at a median time of 108 days before delivery (range 1-263); only 4 women were infected <7 days before delivery) | NR |
| 1 | La Reunion 2005-2006 outbreak; Neonatal cohort | Ramful 2014; 2005-2006 outbreak (overlap with data from Geradin’s 2008) | N=658 maternal infections | NR in this report | Serology and RT-PCR | Serology (IgG and IgM) and/or RT-PCR |
| 1 | La Reunion, 2005-2006 outbreak; Neonatal cohort | Gerardin 2014; 2005-2006 | NR in this report | NR in this report | NR in this report | NR in this report |
| 1 | La Reunion, 2005-2006 outbreak; Neonatal cohort | Gerardin 2016; 2005-2006 | NR in this report | NR in this report | NR in this report | NR in this report |
| 2 | Mayotte (Comoros archipeglagos-Indian Ocean); 2005-2006 outbreak in Western Indian Ocean; Maternal and neonatal sub-cohort | Sissoko 2008 | N=163 pregnant women with serologic evidence of recent infection  No data for N of symptomatic vs asymptomatic maternal infections | NR | Serologic evidence of recent infection (IgM positive and IgG negative or IgM positive and IgG positive) | NR |
| 3 | Sri Lanka; 2006-2007 outbreak | Senanayake 2009 | N=50 seropositive pregnant women (48 followed prospectively and 2 included after their infants developed symptoms) | 4 women were clinically viremic at the time of delivery and 1 was infected in the post-partum period (otherwise 21 other mothers were infected during first trimester; 21 during second trimester and 7 during third trimester) | Serologic diagnosis based on HIA titers >160 (definite infection; n=40) or >80 (possible infection; n=10) | Neonatal IgMs (tested only in 32 infants) |
| 4 | Thailand; 1998-1999 surveillance period; Maternal and neonatal cohort | Watanaveeradej 2006; 1998-1999 | N=28 maternal infections  (Although cord blood from 79 seropositive pregnant women were tested and it was reported that 31% of the cord bloods had higher titers that maternal titers; neonatal outcome data were provided only for 28/79 infected mothers whose infants had a follow-up at 18 months of age; No data for N of symptomatic vs asymptomatic maternal infections) | NR | CHIKV-specific IgG | Cord blood IgG and Persistence of serum IgG up to 18 months of age |
| 5 | Thailand, 2009-2010 outbreak; Maternal and Neonatal cohort | Laoprasopwattana 2016;7-months period 2009-2010 | N=88 with serologic evidence of recent infection (4 IgM+/IgG-, 84 IgM+/IgG+); 5/88 symptomatic maternal CHIKV infections among mothers with recent infection | NR (Timing of infection was only reported for the 5 symptomatic recently infected mothers and in none of them [0/5] maternal infection had occurred during the peripartum period (at 2 months GA(n=2); at 5 months GA (n=1) and at 7 months GA (n=1) | a) Maternal infections based on Serologic testing and b) Maternal infections based serologic testing AND clinical symptoms during pregnancy. (Although it was also reported that mothers with Acute Febrile Illness also had blood RT-PCR; these results were not reported) | Cord blood samples from infants whose mothers had serologic evidence of recent CHIKV infection (although it was also reported that infants with acute febrile illness also had Blood RT-PCR; these results were not reported) |
| 6-8 | Latin America (El Salvador, Santo Domingo, Colombia) , 2014-2015 outbreak | Torres 2016; 2014-2015; Maternal cohort (El Salvador and Santo Domingo) and Neonatal cohort (El Salvador, Dominican Republic and Colombia | N=396 symptomatic infected mothers  **(El Salvador:** 191 symptomatic infected mothers; all 35-40 weeks GA  **Colombia**: NR on the N of infected mothers of the N=37 symptomatic neonates)  **Santo Domingo**: 205 symptomatic infected mothers | No data for N of asymptomatic maternal CHIK infections  N=191 symptomatic infected mothers infected at 35-40 weeks were reported only from El Salvador  (Santo Domingo: NR;  Colombia: NR);  However, the N of intrapartum/peripartum infections was not reported separately | **El Salvador**: Symptoms with IgM (+) and/or RT-PCR  **Colombia:** NR  **Santo Domingo**: symptoms during outbreak without confirmatory testing | **El Salvador:** RT-PCR  **Colombia**: symptoms in infants born to mothers with proven or suspected CHIKV infection  **Santo Domingo:** Clinical symptoms in 1st week of life without history of mosquito bite |
| 9 | Colombia, 2015 | Escobar 2016; 2015 | N=60 hospitalized infected pregnant women (n=5 in 1^st^; n=17 in 2^nd^ and n=38 in 3d trimester); only n=15 women in their 3d trimester completed pregnancy during the hospitalization and 9 required ICU stay | 15 intrapartum/peripartum maternal infections | RT-PCR | RT-PCR |

**Abbreviations:** APFD: antepartum fetal deaths; CHIKV: chikungunya virus; GA: gestational age; ICU: intensive care unit; mo: months; MTCT: mother to child transmission; NA: not available; NR: not reported; PTD: prior to delivery; RT-PCR: reverse transcription polymerase chain reaction; VT: vertical transmission; wks: week

REFERENCES FOR SUPPLEMENTARY MATERIAL

1. Lenglet Y, Barau G, Robillard PY, Randrianaivo H, Michault A, Bouveret A, et al. [Chikungunya infection in pregnancy: Evidence for intrauterine infection in pregnant women and vertical transmission in the parturient. Survey of the Reunion Island outbreak]. J Gynecol Obstet Biol Reprod (Paris). 2006;35(6):578-83. PubMed PMID: 17003745.

2. Robillard PY, Boumahni B, Gerardin P, Michault A, Fourmaintraux A, Schuffenecker I, et al. Vertical maternal fetal transmission of the chikungunya virus - Ten cases among 84 pregnant women. Presse Med. 2006;35(5):785-8. doi: Doi 10.1016/S0755-4982(06)74690-5. PubMed PMID: WOS:000237918800011.

3. Ramful D, Carbonnier M, Pasquet M, Bouhmani B, Ghazouani J, Noormahomed T, et al. Mother-to-child transmission of Chikungunya virus infection. Pediatr Infect Dis J. 2007;26(9):811-5. doi: 10.1097/INF.0b013e3180616d4f. PubMed PMID: WOS:000249455800008.

4. Gerardin P, Barau G, Michault A, Bintner M, Randrianaivo H, Choker G, et al. Multidisciplinary prospective study of mother-to-child chikungunya virus infections on the Island of La Reunion. Plos Med. 2008;5(3):413-23. doi: ARTN 060

10.1371/journal.pmed.0050060. PubMed PMID: WOS:000254928900016.

5. Fritel X, Rollot O, Gerardin P, Gauzere BA, Bideault J, Lagarde L, et al. Chikungunya virus infection during pregnancy, Reunion, France, 2006. Emerg Infect Dis. 2010;16(3):418-25. doi: 10.3201/eid1603.091403. PubMed PMID: 20202416; PubMed Central PMCID: PMCPMC3322036.

6. Ramful D, Samperiz S, Fritel X, Michault A, Jaffar-Bandjee MC, Rollot O, et al. Antibody kinetics in infants exposed to Chikungunya virus infection during pregnancy reveals absence of congenital infection. J Infect Dis. 2014;209(11):1726-30. doi: 10.1093/infdis/jit814. PubMed PMID: 24338351.

7. Gerardin P, Samperiz S, Ramful D, Boumahni B, Bintner M, Alessandri JL, et al. Neurocognitive outcome of children exposed to perinatal mother-to-child Chikungunya virus infection: the CHIMERE cohort study on Reunion Island. PLoS Negl Trop Dis. 2014;8(7):e2996. doi: 10.1371/journal.pntd.0002996. PubMed PMID: 25033077; PubMed Central PMCID: PMCPMC4102444.

8. Sissoko D, Malvy D, Giry C, Delmas G, Paquet C, Gabrie P, et al. Outbreak of Chikungunya fever in Mayotte, Comoros archipelago, 2005-2006. T Roy Soc Trop Med H. 2008;102(8):780-6. doi: 10.1016/j.trstmh.2008.02.018. PubMed PMID: WOS:000258201600008.

9. Watanaveeradej V, Endy TP, Simasathien S, Kerdpanich A, Polprasert N, Aree C, et al. Transplacental chikungunya virus antibody kinetics, Thailand. Emerg Infect Dis. 2006;12(11):1770-2. PubMed PMID: WOS:000241573900025.

10. Senanayake MP SS, Vidanage KK, Gunassena S, Lamabadusurlya SP. Vertical transmission in Chikungunya infection. Cylon Med J. 2009;54(2):47-50.

11. Laoprasopwattana K, Suntharasaj T, Petmanee P, Suddeaugrai O, Geater A. Chikungunya and dengue virus infections during pregnancy: seroprevalence, seroincidence and maternal-fetal transmission, southern Thailand, 2009-2010. Epidemiol Infect. 2016;144(2):381-8. doi: 10.1017/S0950268815001065. PubMed PMID: WOS:000368638100020.

12. Torres JR, Falleiros-Arlant LH, Duenas L, Pleitez-Navarrete J, Salgado DM, Brea-Del Castillo J. Congenital and perinatal complications of chikungunya fever: a Latin American experience. Int J Infect Dis. 2016;51:85-8. doi: 10.1016/j.ijid.2016.09.009. PubMed PMID: WOS:000388326700020.

13. Escobar M, Nieto AJ, Loaiza-Osorio S, Barona JS, Rosso F. Pregnant Women Hospitalized with Chikungunya Virus Infection, Colombia, 2015. Emerg Infect Dis. 2017;23(11):1777-83. doi: 10.3201/eid2311.170480. PubMed PMID: WOS:000413109500002.

1. Lenglet Y, Barau G, Robillard PY, Randrianaivo H, Michault A, Bouveret A, et al. [Chikungunya infection in pregnancy: Evidence for intrauterine infection in pregnant women and vertical transmission in the parturient. Survey of the Reunion Island outbreak]. J Gynecol Obstet Biol Reprod (Paris). 2006;35(6):578-83. PubMed PMID: 17003745.

2. Robillard PY, Boumahni B, Gerardin P, Michault A, Fourmaintraux A, Schuffenecker I, et al. Vertical maternal fetal transmission of the chikungunya virus - Ten cases among 84 pregnant women. Presse Med. 2006;35(5):785-8. doi: Doi 10.1016/S0755-4982(06)74690-5. PubMed PMID: WOS:000237918800011.

3. Ramful D, Carbonnier M, Pasquet M, Bouhmani B, Ghazouani J, Noormahomed T, et al. Mother-to-child transmission of Chikungunya virus infection. Pediatr Infect Dis J. 2007;26(9):811-5. doi: 10.1097/INF.0b013e3180616d4f. PubMed PMID: WOS:000249455800008.

4. Gerardin P, Barau G, Michault A, Bintner M, Randrianaivo H, Choker G, et al. Multidisciplinary prospective study of mother-to-child chikungunya virus infections on the Island of La Reunion. Plos Med. 2008;5(3):413-23. doi: ARTN 060

10.1371/journal.pmed.0050060. PubMed PMID: WOS:000254928900016.

5. Fritel X, Rollot O, Gerardin P, Gauzere BA, Bideault J, Lagarde L, et al. Chikungunya virus infection during pregnancy, Reunion, France, 2006. Emerg Infect Dis. 2010;16(3):418-25. doi: 10.3201/eid1603.091403. PubMed PMID: 20202416; PubMed Central PMCID: PMCPMC3322036.

6. Ramful D, Samperiz S, Fritel X, Michault A, Jaffar-Bandjee MC, Rollot O, et al. Antibody kinetics in infants exposed to Chikungunya virus infection during pregnancy reveals absence of congenital infection. J Infect Dis. 2014;209(11):1726-30. doi: 10.1093/infdis/jit814. PubMed PMID: 24338351.

7. Gerardin P, Samperiz S, Ramful D, Boumahni B, Bintner M, Alessandri JL, et al. Neurocognitive outcome of children exposed to perinatal mother-to-child Chikungunya virus infection: the CHIMERE cohort study on Reunion Island. PLoS Negl Trop Dis. 2014;8(7):e2996. doi: 10.1371/journal.pntd.0002996. PubMed PMID: 25033077; PubMed Central PMCID: PMCPMC4102444.

8. Sissoko D, Malvy D, Giry C, Delmas G, Paquet C, Gabrie P, et al. Outbreak of Chikungunya fever in Mayotte, Comoros archipelago, 2005-2006. T Roy Soc Trop Med H. 2008;102(8):780-6. doi: 10.1016/j.trstmh.2008.02.018. PubMed PMID: WOS:000258201600008.

9. Watanaveeradej V, Endy TP, Simasathien S, Kerdpanich A, Polprasert N, Aree C, et al. Transplacental chikungunya virus antibody kinetics, Thailand. Emerg Infect Dis. 2006;12(11):1770-2. PubMed PMID: WOS:000241573900025.

10. Senanayake MP SS, Vidanage KK, Gunassena S, Lamabadusurlya SP. Vertical transmission in Chikungunya infection. Cylon Med J. 2009;54(2):47-50.

11. Laoprasopwattana K, Suntharasaj T, Petmanee P, Suddeaugrai O, Geater A. Chikungunya and dengue virus infections during pregnancy: seroprevalence, seroincidence and maternal-fetal transmission, southern Thailand, 2009-2010. Epidemiol Infect. 2016;144(2):381-8. doi: 10.1017/S0950268815001065. PubMed PMID: WOS:000368638100020.

12. Torres JR, Falleiros-Arlant LH, Duenas L, Pleitez-Navarrete J, Salgado DM, Brea-Del Castillo J. Congenital and perinatal complications of chikungunya fever: a Latin American experience. Int J Infect Dis. 2016;51:85-8. doi: 10.1016/j.ijid.2016.09.009. PubMed PMID: WOS:000388326700020.

13. Escobar M, Nieto AJ, Loaiza-Osorio S, Barona JS, Rosso F. Pregnant Women Hospitalized with Chikungunya Virus Infection, Colombia, 2015. Emerg Infect Dis. 2017;23(11):1777-83. doi: 10.3201/eid2311.170480. PubMed PMID: WOS:000413109500002.

1. Lenglet Y, Barau G, Robillard PY, Randrianaivo H, Michault A, Bouveret A, et al. [Chikungunya infection in pregnancy: Evidence for intrauterine infection in pregnant women and vertical transmission in the parturient. Survey of the Reunion Island outbreak]. J Gynecol Obstet Biol Reprod (Paris). 2006;35(6):578-83. PubMed PMID: 17003745.

2. Robillard PY, Boumahni B, Gerardin P, Michault A, Fourmaintraux A, Schuffenecker I, et al. Vertical maternal fetal transmission of the chikungunya virus - Ten cases among 84 pregnant women. Presse Med. 2006;35(5):785-8. doi: Doi 10.1016/S0755-4982(06)74690-5. PubMed PMID: WOS:000237918800011.

3. Ramful D, Carbonnier M, Pasquet M, Bouhmani B, Ghazouani J, Noormahomed T, et al. Mother-to-child transmission of Chikungunya virus infection. Pediatr Infect Dis J. 2007;26(9):811-5. doi: 10.1097/INF.0b013e3180616d4f. PubMed PMID: WOS:000249455800008.

4. Gerardin P, Barau G, Michault A, Bintner M, Randrianaivo H, Choker G, et al. Multidisciplinary prospective study of mother-to-child chikungunya virus infections on the Island of La Reunion. Plos Med. 2008;5(3):413-23. doi: ARTN 060

10.1371/journal.pmed.0050060. PubMed PMID: WOS:000254928900016.

5. Fritel X, Rollot O, Gerardin P, Gauzere BA, Bideault J, Lagarde L, et al. Chikungunya virus infection during pregnancy, Reunion, France, 2006. Emerg Infect Dis. 2010;16(3):418-25. doi: 10.3201/eid1603.091403. PubMed PMID: 20202416; PubMed Central PMCID: PMCPMC3322036.

6. Ramful D, Samperiz S, Fritel X, Michault A, Jaffar-Bandjee MC, Rollot O, et al. Antibody kinetics in infants exposed to Chikungunya virus infection during pregnancy reveals absence of congenital infection. J Infect Dis. 2014;209(11):1726-30. doi: 10.1093/infdis/jit814. PubMed PMID: 24338351.

7. Gerardin P, Samperiz S, Ramful D, Boumahni B, Bintner M, Alessandri JL, et al. Neurocognitive outcome of children exposed to perinatal mother-to-child Chikungunya virus infection: the CHIMERE cohort study on Reunion Island. PLoS Negl Trop Dis. 2014;8(7):e2996. doi: 10.1371/journal.pntd.0002996. PubMed PMID: 25033077; PubMed Central PMCID: PMCPMC4102444.

8. Sissoko D, Malvy D, Giry C, Delmas G, Paquet C, Gabrie P, et al. Outbreak of Chikungunya fever in Mayotte, Comoros archipelago, 2005-2006. T Roy Soc Trop Med H. 2008;102(8):780-6. doi: 10.1016/j.trstmh.2008.02.018. PubMed PMID: WOS:000258201600008.

9. Watanaveeradej V, Endy TP, Simasathien S, Kerdpanich A, Polprasert N, Aree C, et al. Transplacental chikungunya virus antibody kinetics, Thailand. Emerg Infect Dis. 2006;12(11):1770-2. PubMed PMID: WOS:000241573900025.

10. Senanayake MP SS, Vidanage KK, Gunassena S, Lamabadusurlya SP. Vertical transmission in Chikungunya infection. Cylon Med J. 2009;54(2):47-50.

11. Laoprasopwattana K, Suntharasaj T, Petmanee P, Suddeaugrai O, Geater A. Chikungunya and dengue virus infections during pregnancy: seroprevalence, seroincidence and maternal-fetal transmission, southern Thailand, 2009-2010. Epidemiol Infect. 2016;144(2):381-8. doi: 10.1017/S0950268815001065. PubMed PMID: WOS:000368638100020.

12. Torres JR, Falleiros-Arlant LH, Duenas L, Pleitez-Navarrete J, Salgado DM, Brea-Del Castillo J. Congenital and perinatal complications of chikungunya fever: a Latin American experience. Int J Infect Dis. 2016;51:85-8. doi: 10.1016/j.ijid.2016.09.009. PubMed PMID: WOS:000388326700020.

13. Escobar M, Nieto AJ, Loaiza-Osorio S, Barona JS, Rosso F. Pregnant Women Hospitalized with Chikungunya Virus Infection, Colombia, 2015. Emerg Infect Dis. 2017;23(11):1777-83. doi: 10.3201/eid2311.170480. PubMed PMID: WOS:000413109500002.

14. Touret Y, Randrianaivo H, Michault A, Schuffenecker I, Kauffmann E, Lenglet Y, et al. Early maternal-fetal transmission of the Chikungunya virus. Presse Med. 2006;35(11):1656-8. doi: Doi 10.1016/S0755-4982(06)74874-6. PubMed PMID: WOS:000242164400010.

15. Robin S, Rainful D, Le Seach F, Jaffar-Bandjee MC, Rigou G, Alessandri JL. Neurologic manifestations of pediatric chikungunya infection. J Child Neurol. 2008;23(9):1028-35. doi: 10.1177/0883073808314151. PubMed PMID: WOS:000258841800007.

16. Gerardin P, Couderc T, Randrianaivo H, Fritel X, Lecuit M. CHIKUNGUNYA VIRUS-ASSOCIATED ENCEPHALITIS: A COHORT STUDY ON LA REUNION ISLAND, 2005-2009 Response. Neurology. 2016;86(21):2025-6. PubMed PMID: WOS:000376959900023.

17. Boumahni B, Kaplan C, Clabe A, Randrianaivo H, Lanza F. Maternal-fetal chikungunya infection associated with Bernard-Soulier syndrome. Arch Pediatrie. 2011;18(3):272-5. doi: 10.1016/j.arcped.2010.12.002. PubMed PMID: WOS:000288186400006.

18. Alvarado-Socarras JL, Ocampo-Gonzalez M, Vargas-Soler JA, Rodriguez-Morales AJ, Franco-Paredes C. Congenital and Neonatal Chikungunya in Colombia. J Pediatr Infect Dis. 2016;5(3):E17-E20. doi: 10.1093/jpids/piw021. PubMed PMID: WOS:000386138100001.

19. Bandeira AC, Campos GS, Sardi SI, Rocha VFD, Rocha GCM. Neonatal encephalitis due to Chikungunya vertical transmission: First report in Brazil. IDCases. 2016;5:57-9. doi: 10.1016/j.idcr.2016.07.008. PubMed PMID: WOS:000399150800019.

20. Evans-Gilbert T. Case Report: Chikungunya and Neonatal Immunity: Fatal Vertically Transmitted Chikungunya Infection. Am J Trop Med Hyg. 2017;96(4):913-5. doi: 10.4269/ajtmh.16-0491. PubMed PMID: WOS:000401763000027.

21. Karthiga V, Kommu PPK, Krishnan L. Perinatal chikungunya in twins. J Pediatr Neurosci. 2016;11(3):223-4. doi: 10.4103/1817-1745.193369. PubMed PMID: WOS:000390115700012.

22. Khandelwal K, Aara N, Ghiya BC, Bumb RA, Satoskar AR. Centro-Facial Pigmentation in Asymptomatic Congenital Chikungunya Viral Infection. J Paediatr Child H. 2012;48(6):542-3. doi: 10.1111/j.1440-1754.2012.02484.x. PubMed PMID: WOS:000305186200021.

23. Kumar N, Gupta V, Thomas N. Brownie-nose: Hyperpigmentation in Neonatal Chikungunya. Indian Pediatr. 2014;51(5):419-. PubMed PMID: WOS:000336049800023.

24. Lyra PPR, Campos GS, Bandeira ID, Sardi SI, Costa LFD, Santos FR, et al. Congenital Chikungunya Virus Infection after an Outbreak in Salvador, Bahia, Brazil. Ajp Rep. 2016;6(3):E299-E300. doi: 10.1055/s-0036-1587323. PubMed PMID: WOS:000382531200008.

25. Passi GR, Khan YZ, Chitnis DS. Chikungunya infection in neonates. Indian Pediatr. 2008;45(3):240-2. PubMed PMID: WOS:000254357300016.

26. Boumahni B, Bintner M. [Five-year outcome of mother-to-child transmission of chikungunya virus]. Med Trop (Mars). 2012;72 Spec No:94-6. PubMed PMID: 22693938.

27. Pinzon-Redondo H, Paternina-Caicedo A, Barrios-Redondo K, Zarate-Vergara A, Tirado-Perez I, Fortich R, et al. RISK FACTORS FOR SEVERITY OF CHIKUNGUNYA IN CHILDREN A Prospective Assessment. Pediatr Infect Dis J. 2016;35(6):702-4. doi: 10.1097/Inf.0000000000001135. PubMed PMID: WOS:000379343700024.

28. Shenoy S, Pradeep GCM. Neurodevelopmental Outcome of Neonates with Vertically Transmitted Chikungunya Fever with Encephalopathy. Indian Pediatr. 2012;49(3):238-40. PubMed PMID: WOS:000304110800015.

29. Shrivastava A, Beg MW, Gujrati C, Gopalan N, Rao PVL. Management of a Vertically Transmitted Neonatal Chikungunya Thrombocytopenia. Indian J Pediatr. 2011;78(8):1008-9. doi: 10.1007/s12098-011-0371-7. PubMed PMID: WOS:000293143700015.

30. Valamparampil JJ, Chirakkarot S, Letha S, Jayakumar C, Gopinathan KM. Clinical profile of Chikungunya in infants. Indian J Pediatr. 2009;76(2):151-5. doi: 10.1007/s12098-009-0045-x. PubMed PMID: WOS:000264631100003.

31. Vasani R, Kanhere S, Chaudhari K, Phadke V, Mukherjee P, Gupta S, et al. Congenital Chikungunya-A Cause of Neonatal Hyperpigmentation. Pediatr Dermatol. 2016;33(2):209-12. doi: 10.1111/pde.12650. PubMed PMID: WOS:000373067800055.

32. Villamil-Gomez W, Alba-Silvera L, Menco-Ramos A, Gonzalez-Vergara A, Molinares-Palacios T, Barrios-Corrales M, et al. Congenital Chikungunya Virus Infection in Sincelejo, Colombia: A Case Series. J Trop Pediatrics. 2015;61(5):386-92. doi: 10.1093/tropej/fmv051. PubMed PMID: WOS:000365384300010.

33. Rodriguez-Nieves M, Garcia-Garcia I, Garcia-Fragoso L. Perinatally Acquired Chikungunya Infection: The Puerto Rico Experience. Pediatr Infect Dis J. 2016;35(10):1163. doi: 10.1097/INF.0000000000001261. PubMed PMID: 27622689.

34. Gopakumar H, Ramachandran S. Congenital chikungunya. J Clin Neonatol. 2012;1(3):155-6. doi: 10.4103/2249-4847.101704. PubMed PMID: 24027715; PubMed Central PMCID: PMCPMC3762016.
