# Supplementary material for: Mother-to-child transmission of Chikungunya virus: A systematic review and meta-analysis
Source: PLoS Negl Trop Dis. 2018 Jun 13;12(6):e0006510. doi: 10.1371/journal.pntd.0006510 (PMC6075784; doi:10.1371/journal.pntd.0006510)
Supplement: S3 Table — (DOCX) [file pntd.0006510.s003.docx]

**S3 Table:** Risks from Maternal CHIKV-infections during gestation (MTCT-risk, APFD-risk, APFD-CHIKV-confirmed-risk, Symptomatic disease-risk; Neonatal death-risk) [1-13]

| **Cohorts** | **Author (year; Outbreak location; Outbreak Period)** | **MTCT-risk**  **(% per total maternal infections)** | **MTCT-risk from Antepartum or Peripartum maternal infections**  **(% per total maternal infections during these periods)** | **MTCT-risk from intrapartum maternal infections**  **(% per total near term maternal infection)** | - **APFD-risk (% per total maternal infections)** - **CHIKV-confirmed-APFD-risk** | - **Symptomatic neonatal disease risk among maternal infections during gestation (%, n/N)** - **Symptomatic neonatal disease risk among intrapartum maternal infections during gestation (%, n/N)** - **Symptomatic neonatal disease risk among antepartum/peripartum maternal infections during gestation (%, n/N)** | - **Neonatal Death risk among maternal infections (%; n/N)** - **Neonatal deaths risk among neonatal infections (%; n/N)** |
| --- | --- | --- | --- | --- | --- | --- | --- |
| 1 | Lenglet 2006; La Reunion; 6/2005-2/2006 (possible overlap with Gerardin’ s 2008 data for this period) | - Overall rate NR in this report - By extrapolation of data of symptomatic neonatal cases then the overall MTCT-risk was at least 10.60% (n=16 symptomatic neonatal /151) among symptomatic maternal infections during gestation | Overall rate NR in this report.  By extrapolation of data of symptomatic neonatal cases:  0% (0/116) for infants from antepartum/peripartum maternal infections with mothers non-viremic at the time of delivery | Overall rate NR in this report.  By extrapolation of data of symptomatic neonatal cases the risk was at least:  48.48% (16/33) from intrapartum maternal infection -infants born to viremic mothers at time of delivery; (there were also 2 APFD/151 women not due to CHIKV infection) | - Overall: 7.28% APFD (11 APFD/ 151) among maternal infections during gestation; - Overall: 1.99% CHIKV-confirmed APFD (3 APFD/151 maternal infection) among maternal infections - N=9 APFD <22 wks - N=2 APFD >22 wks - 1.98% CHIKV-confirmed-APFD <22 wks (3/151) among maternal infections (N=3/9 APFD <22 weeks were also confirmed to be attributed to fetal CHIKV infections by positive AF RT-PCR) - 0% CHIKV-confirmed -APFD >22 wks (0/151) among maternal infections (N=0/2 APFD >22 weeks from maternal CHIKV infection was confirmed to be due to CHIKV infection) | - Overall risk of symptomatic neonatal disease=10.60% (16 /151) among maternal infections during gestation   - Symptomatic neonatal cases: were asymptomatic at the time of birth and developed symptoms within 3-7 ds from birth   - (Rates of prematurity in infected infants were similar to the general population (10% vs 13.9%)) - 48.48% (16/33) risk of symptomatic neonatal disease among the 33 intrapartum maternal infections - 0% (0/116) risk of symptomatic neonatal disease among the 116 maternal infections non-viremic at the time of delivery | - Overall rate NR in this report - Overall rate NR in this report |
| 1 | Robillard 2006; La Reunion; 6/2005-1/2006 (possible overlap with Gerardin’s 2008 data for the same period) | - By extrapolation of data of severely symptomatic neonatal cases then the MTCT-risk was at least 11.9% (10/84) among maternal infections during gestation | Overall rate NR in this report | Overall rate NR in this report | NR | - Overall risk of severely symptomatic neonatal disease=11.9% (10/84) among maternal infections during gestation - 100% (10/10) symptomatic neonates among intrapartum maternal infections   - All 10 severely symptomatic newborns were from maternal infections viremic (PCR +) at the time of birth (all had cutaneous rashes, extremity edema, painful syndrome; 6 requiring intubation; 4 had seizures /meningoencephalitis also requiring intubation and 3 had DIC) - 0% (0/74) symptomatic neonates among antepartum/peripartum maternal infections   - 88% (n=74/84) of the neonates born to mothers with maternal infections distant from the time of delivery were asymptomatic; however, the CHIKV infection status for these infants was not ascertained in this report | - 0% (0 neonatal deaths/84) neonatal death risk among maternal infections during gestation - 0% (0/10) neonatal death risk among severely symptomatic neonates |
| 1 | Touret 2006; La Reunion; 2005-2006 | NR in this report   - (Data were reported only for the mothers of the APFD cases) | Overall rate NR in this report | Overall rate NR in this report | Overall rate NR rate in this report   - Description of the 3 APFD with early maternal infections at 12 weeks (n=1) and 15 weeks (n=2) (confirmed with AF PCR) 4(the N of infected mothers w5as not reported) | Overall rate NR in this report | - Overall rate NR in this report - Overall rate NR in this report |
| 1 | Ramful 2007; La Reunion; 3/2005-4/2006 (possible overlap with Gerardin’s 2008 data for the same period) | - By extrapolation of data of symptomatic neonatal cases then the MTCT risk was at least 6.43% (38/591) among maternal infections during gestation - (In this report, data reported only on 38 mothers of 38 symptomatic newborn infants) - 24/24 RT-PCR tested neonates were positive - 26/30 CHIKV IgM tested neonatal were positive | Overall rate NR in this report | Overall rate NR in this report.  By extrapolation of data of symptomatic neonatal cases:  N=38 symptomatic infected infants/38 infected mothers in the perinatal period (-4ds PTD to +1 day post- delivery) and 18 mothers were symptomatic at the time of delivery | Overall rate NR in this report | - By extrapolation of these data to the 591 maternal infections in this cohort: Risk of 6.43% (38/591) for symptomatic neonatal disease among maternal infections during gestation   - N=38 symptomatic neonates by d3-d7 (23/38 were uncomplicated; 15/38 were complicated neonatal infections) - NR per intrapartum maternal infections in this report - NR per antepartum/peripartum maternal infections in this report | - 0.17% (1/591) neonatal death risk among maternal infections (by extrapolation to cohort data) - 2.6% (1/38) neonatal deaths among 38 symptomatic neonatal infections |
| 1 | Gerardin 2008; La Reunion; 6/2005-12/2006 | - Overall rate NR in this report - By extrapolation of data of symptomatic neonatal cases then the overall MTCT-risk was at least 3.2% (19/591) among symptomatic maternal infections during gestation - (neonatal infection status was ascertained by neonatal serology or RT-PCR only for 591 neonates; thus for 591 of the total 739 infected mothers in this cohort) - (Only 5% [39/739] of maternal infections were intrapartum and another 3% [22/739] were peripartum) - All neonatal infections occurred in the context of near term deliveries, at a median GA of 38 wks (range 35-40 wks); in the context of maternal viremia; - All infected neonates had a median onset of symptoms at 4 days (range 3-7) after births: pain, prostration, fever, thrombocytopenia; - The absence of neonatal viremia on DOL #1 was consistent with intrapartum mother-to-fetal transmission; - The Placental Viral Load was higher in transmitting than in non transmitting women (however the N of placenta tested was not reported) | Overall rate NR in this report.  By extrapolation of data of symptomatic neonatal cases:  0% [0/678] from antepartum maternal infections >7 ds PTD and  0% [0/22] from peripartum maternal infections from -7 ds to -3 ds PTD | Overall rate NR in this report.  By extrapolation of data of symptomatic neonatal cases the risk was at least:  48.72% [19/39] from only intrapartum maternal infections from -2 ds PTD to +2 ds after delivery | - Overall: 2.17% APFD [16 APFD /739] among maternal infections during gestation - 0.44% (3/739) CHIKV-confirmed APFD among maternal infections during gestation - 0.94% APFDs <22 wks (7 APFD from maternal infections <22 wks /678) among maternal infections during gestation; - 1.9% APFD >22 wks (9 APFD from maternal infections>22 wks/678) among maternal infections during gestation - 0.44% (3/739) CHIK-confirmed APFD <22 wks among maternal infections during gestation - 0% CHIKV-confirmed APFD >22 wks among maternal infections during gestation - No difference in APFD >22 wks from years prior to the CHIKV outbreaks (e.g in 2001=0.5%; in 2002=0.7%; in 2003=1%; in 2004=1.2%) | - Overall risk of symptomatic neonatal disease=3.21% (19/591) among maternal infections during gestation   - Risk of severely symptomatic neonatal disease=1.69% (n=10 /591) among maternal infections during gestation - 48.72% (19/39) risk for symptomatic neonatal disease among intrapartum maternal infections   - Risk of 15.64% (10/39) for severely symptomatic neonatal disease from intrapartum maternal infections during gestation   - 53% [10/19] of neonatal infections from maternal infections in the intrapartum period were severely symptomatic (encephalopathy [n=9], pathologic brain MRIs and development of persistent disabilities [n=4]; hemorrhagic fever [n=1])   - All symptomatic infants developed symptoms between d3-d7; but at birth were asymptomatic without detection of neonatal viremia at DOL #1   - There were also 3 early APFD   - The remaining 47% [9/19] of neonatal infections had normal clinical status and Brain MRI in the F/up) - 0% (0/700) risk for symptomatic neonatal disease among antepartum/peripartum maternal infections | - Overall rate NR in this report - Overall rate NR in this report |
| 1 | Fritel 2010; La Reunion; 4/2006-11/2006 (possible overlap with Gerardin’s 2008 data for this period) | Overall risk NR in this report   - (in this case-control analysis, no VT rates were reported from this cohort; these VT rates were reported in the Gerardin et al 2008 study) - (0.16% (1/625) of examined placentas from infected mothers had histologic signs compatible with viral infection) - Among 658 symptomatic maternal infections during gestations: - 15% (99/658) occurred during first trimester - 59% (387/658) occurred during second trimester - 26% (172/658) occurred during third trimester - Only 4 women (0.6%) were infected in the 7 ds PTD | Overall rate NR in this report | 25% (1 neonatal infection/4 intrapartum/peripartum maternal infections <7 ds PTD) | Overall 1.5% [10 APFD/656] among maternal infections; nevertheless CHIKV-testing was done only in 4 APFDs and was positive in 3 (0.45%)  0.45% CHIKV-confirmed (3/656) among maternal infections  N=5 APFDs for maternal infections >22 weeks (RT-PCR done in 3 placentas/or AF of these stillbirths and was (+) in 2/3 APFDs); and  N=5 APFDs for maternal infections <22 weeks (RT PCR was done on trophoblast tissue for 1/ 5 APFDs and was negative.  (Study began in April 2006, after the CHIKV outbreak had peaked; thus, the study does not describe the consequences of the outbreak on the risk for APFD during the peak of the outbreak during the first quarter of 2006)  Comparison of APFD cases in infected vs non-infected pregnant women   - 5 vs 8 APFD >22 wks - 5 vs 8 APFD <22 wks   Among 2 cases of CHIKV-confirmed APFD: maternal CHIKV symptoms began 25 and 70 days respectively before the fetal loss | Overall rate NR in this report | - Overall rate NR in this report - Overall rate NR in this report |
| 1 | Ramful 2014; La Reunion; 2005-2006 outbreak (possible overlap with data from Geradin’s 2008) | Overall rate NR in this report | Overall rate NR in this report | Overall rate NR in this report | Overall rate NR in this report | - Overall rate NR - 1 of symptomatic RT-PCR + in serum and CSF case reported from a maternal symptomatic infection 1 d PTD - (only 591 neonates were tested/658 maternal infections [90%]) - The focus of this study was on the kinetics of neonatal CHIKV serology: Neonatal IgG can be present >12 mo of age in >75% of infants from maternal infections in first trimester; in >30% of 6 infants if maternal infections in second trimester and only in 1% if maternal infections in third trimester - Among 368 initially seropositive infants with complete serologic f/up the mean time to seroconversion to IgG negative status as 7.7 mo and the maximum was 24 mo - Among all neonates born to CHIKV infected mothers during gestation (independently of the neonatal CHIKV-infection status):43% of neonates have positive IgG if maternal infection occurred ≤2 wks PTD vs 99% of maternal infections were >15 ds PTD - Reported also a case report of a symptomatic neonatal CHIKV-infections who were initially negative for CHIKV IgG and IgM screening, and subsequently developed IgM in week 3 and IgG in week 4 after birth; with high IgG levels remained at 18 mo of age (confirming neonatal infection) | - Overall rate NR in this report - Overall rate NR in this report |
| 1 | Gerardin 2014; La Reunion; 6/2005-4/2006 | - By extrapolation of data of symptomatic neonatal cases then the MTCT-risk was at least 6.26% (37/591) among maternal infections during gestation - As there were at least 37 symptomatic infants from maternal infections during gestation: 33 participated in this long-term-f/up study (12 severely encephalopathic+ 22 with mild/moderate prostration) plus 4 symptomatic who could not be included in this f/up (1 encephalopathic+2 neonatal prostrations+1 severe CP) - However, the total number of neonatal infections (symptomatic + asymptomatic) was not reported in this report | Overall rate NR in this report | Overall rate NR in this report | Overall rate NR in this report | - Overall risk of symptomatic neonatal disease=6.26% (37/591) of all maternal infections during gestation - NR per intrapartum maternal infections in this report - NR per antepartum/peripartum maternal infections in this report - Rate of long-term global neurodevelopmental delay at 2 years of age=2.8% (17/591) of maternal infections during gestation - Long term outcome were reported at 2 years of age among 33 infected infants from maternal infections during gestationd - The Development quotients (DQ) scores were lower in p-CHIKV compared to uninfected children; - 51% (n = 17/33) of symptomatic p-CHIK had a global neurodevelopmental delay (GND) at 2 years of age compared to 15% (n = 21) of uninfected children (P<0.001); - Of these 33 c-p-CHIKV infected infants: 12 severely encephalopathic (seizures, coma or cytotoxic or vasogenic edema on early brain MRI) and 21 non-severely prostrated infants (conscious but unable to feed, or conscious with ventilatory support for sedation and analgesia) - 75% GDD among encephalopathic children vs 38% among mildly prostrated non encephalopathic children - After adjustment for maternal social situation, small for GA, and HC; p-CHIKV infected infants had a 2.79 higher incidence rate of GND | - Overall rate NR in this report - Overall rate NR in this report |
| 1 | Gerardin 2016; La Reunion; 2005-2006 | Overall rate NR in this report | Overall rate NR in this report | Overall rate NR in this report | Overall rate NR in this report | NR in this report   - Among the total number of cases with CNS-CHIKV disease (n=17 infants evaluated at 38 months of age) for only 3 cases it was specifically mentioned that they had occurred in infants infected from maternal infections during gestation (1 developed cerebral palsy and blindness; while the other 2 had normal neurodevelopmental outcome at 38 months of age) | - Overall rate NR in this report - Overall rate NR in this report |
| 2 | Sissoko 2008; Mayotte (Indian Ocean); 2005-2006 | - By extrapolation of data of symptomatic neonatal cases then the MTCT-risk was at least 5.52% (9/163) among maternal infections during gestation | NR | NR | - 0% (0/163) APFD among maternal infections during gestation - 0% (0/163) CHIKV-confirmed APFD among maternal infections during gestation | - Overall risk of symptomatic neonatal disease=5.52% (9/163) among maternal infections during gestation   - (9 hospitalized symptomatic cases of infected infants/163 recent maternal infections during gestation) - NR number of intrapartum maternal infections - NR number of antepartum/peripartum maternal infections | - NR in this report - NR in this report |
| 3 | Watanaveeradej 2006; Thailand; 1998-1999 | Overall MTCT-risk=0% (0/28) among maternal infections during gestation   - All 28 infants of these infected mothers lost their initially positive IgG antibodies by 9 months of age and thus were considered not infected | NR | NR | NR | - 0% (0/28) among maternal infections during gestation - Neonatal outcomes were provided for 28 infants from 79 CHIK + mothers who agreed to have serologic f/up of their infants until 18 months of age) - NR number of intrapartum maternal infections - NR number of antepartum/peripartum maternal infections | NA (there were no neonatal infections from maternal infections during gestation) |
| 4 | Senanayake 2009; Shri Lanka; 2006-2007 | Overall MTCT-risk=14.0% (7/50) among maternal infections during gestation   - However, neonatal CHIKV IgM was done in only 32 neonates among the 50 maternal infections and confirmed positive in only 7 - 2 infants from maternal infections in first trimester; 1 in second trimester; 3 in third trimester and 1 from post-partum maternal infections) - (Only 8% [4/50] of women were viremic at the time of delivery) - VT of c-p CHIK infections were confirmed from maternal infections at all trimesters - (Shri Lanka had not previously reported CHIKV outbreaks since 1965) | NR | 100% (4/4) of intrapartum maternal infections   - However, only 1 of these 4 infants was CHIKV IgM positive - (All 4 of these infants were symptomatic) | - 6% APFD (3 APFD/ 50) among maternal infections; - however, these were not tested for their CHIKV infection status - N=3 APFD (including 2 abortions, 1 during acute maternal symptomatic CHIKV infection and the 1 at 33 weeks GA with multiple congenital anomalies) | - Overall risk of severely symptomatic neonatal disease=8% (4/50) among maternal infections during gestation   - However, only 2% (1/50) of maternal infections were also confirmed to be IgM positive - 100% (4/4) risk of severely symptomatic neonatal disease from intrapartum maternal infections   - There were only 4 symptomatic neonates from intrapartum maternal infections; but only 1 of those was confirmed CHIKV IgM positive   - There were 19 infants with some clinical abnormalities reported plus 3 APFD: but only 7/19 neonates were also CHIKV IgM positive - 0% (0/46) risk of severely symptomatic neonatal disease from antepartum/peripartum maternal infections | - 0% (0/50) neonatal death risk among maternal infections - 0% (0/19) neonatal death risk among symptomatic neonatal infections |
| 5 | Laoprasopwattana 2016; Thailand; 2009-2010 | Overall MTCT-risk=0% (0/88) among maternal infections during gestation   - 0 of recent maternal infections had a positive cord blood testing for CHIKV IgM - Neonatal infections were confirmed only by cord blood testing for CHIK IgM and there were NO neonatal infections identified; - (NR numbers of intra/peri-partum maternal infections - Among the 5/88 symptomatic maternal infections during gestation, none was in the intra-peripartum period | NR | NR | NR | - 0% (0/88) among maternal infections during gestation   - Neonatal infections were confirmed only by cord blood testing for CHIK IgM   - And there were NO neonatal infections - NR number of intrapartum maternal infections - NR number of antepartum/peripartum maternal infections | - 0% (0/88) neonatal death risk among maternal infections during gestation - NA (there were no neonatal infections) |
| 6 | Torres 2016; Latin America; 2014-2015 | - El Salvador: - By extrapolation of data of symptomatic neonatal cases, then the overall MTCT-risk was at least 27.7% (53/191) among symptomatic maternal infections during gestation - 100% of infected infants were also symptomatic - Neonatal infections confirmed by neonatal RT-PCR | NR | NR | El Salvador:   - 0% (0/191) APFD among maternal infections during gestation - 0% (0/191) CHIKV-confirmed APFD among maternal infections during gestation | a) El Salvador: Overall rate of symptomatic disease=27.7% [53/191] of maternal infections during gestation   - 100% of infected infants were also symptomatic   - No congenital malformations were identified - NR number of intrapartum maternal infections - NR number of antepartum/peripartum maternal infections | El Salvador: NR |
| NA | Torres 2016; Latin America; 2014-2015 | 1. Colombia:  - NR overall VT rate - (37 infected infants, but the N of infected mothers during gestation was not reported) | NR | NR | Colombia: NR APFD | b) Colombia: NR (Data on 37 symptomatic newborns, but no data on N of maternal infections to estimate rate of symptomatic congenital infections); | Colombia: NR |
| 7 | Torres 2016; Latin America; 2014-2015 | c) Santo Domingo:   - By extrapolation of data of symptomatic neonatal cases, then the overall MTCT-risk was at least 48.29% (99/205) among symptomatic maternal infections. - The diagnosis of neonatal infections was based on clinical symptoms only and not on any confirmatory testing | NR | NR | Santo Domingo: NR APFD | c) Santo Domingo:   - Overall risk of symptomatic disease=48.29% (99/ 205) of maternal infections during gestation - NR number of intrapartum maternal infections - NR number of antepartum/peripartum maternal infections | Santo Domingo: 5.1% [4/79] neonatal death risk among symptomatic neonatal disease  (neonatal mortality data were reported for only 79/99 symptomatic neonatal disease) |
| 8 | Escobar 2017; Colombia, 2015 | Overall rate NR for N=60 maternal infections during gestation   - But it was 0% (0/15) at least among intrapartum/peripartum maternal infections | 0% (0/12) among maternal infections during peripartum period | 0% (0/3) among maternal infections during intrapartum period (average 6.3±1.9 days from onset of Sx to labor)   - All maternal infections during intrapartum period received tocolysis to delay time of birth | 1.66% APFD (1 APFD in 1st trimester/60) among maternal infections;   - no confirmation was done for the fetal CHIKV status for this APFD - (however, 2/3 of the pregnant women in this cohort were in their third trimester) - this APFD occurred from an early gestation acute maternal CHIKV infection | - 0% (0/15) risk of symptomatic neonatal disease among intrapartum/peripartum maternal infections; (only 6 neonates were tested by RT-PCR, and all were negative)   - These 15 infants were born during their mother’s acute CHIKV infection   - The average interval from onset of maternal symptoms to delivery was 6.3+/- 1.4 days   - Tocolysis was implemented in 3/15 (20%) of women with intrapartum fever to delay labor (an approach previously used also in their institution for prevention of vertical transmission of other arboviral infections e.g. Dengue fever)   - Tocolysis with nifedipine for 2.3±0.94 days, to delay labor and prevent births during the period of intrapartum fever - 0% (0/3) risk of symptomatic neonatal disease among intrapartum maternal infections - 0% (0/12) risk of symptomatic neonatal disease among antepartum/peripartum maternal infections | 0% (0/15) neonatal deaths among maternal infection during peripartum/intrapartum period  NA (there were no neonatal infections) |
|  | **TOTAL**  **(Pooled Risk across cohorts^c^)** | **Overall MTCT-risk was at least 15.48% (206/1331) among maternal infections during gestation ^a^** | **0% MTCT-risk (0/712) among antepartum/peripartum maternal infections** | **At least 50.0% MTCT-risk (23/46) among intrapartum maternal infections** (maternal viremia at time of delivery) (La Reunion, Shri Lanka and Colombia Cohorts only) | - **1.66% (20/1203) APFD among maternal infections during gestation** - **0.25%(3/1203) CHIKV-confirmed APFD among maternal infections during gestation** | - **Overall risk of symptomatic neonatal disease: 15.25% (203/1331) among maternal infections during gestation**   - **50.00% symptomatic neonatal disease-risk (23/46) among intrapartum maternal infections**   - **0% symptomatic neonatal disease risk (0/758) among antepartum/peripartum maternal infections** | - **0.60% (5/832) neonatal death risk among maternal infections during gestations** - **2.75% (5/182) neonatal death risk among neonatal infections, from maternal infections during gestation** |
|  | **Overall risk by REM synthesis (Risk and 95% CIs)** | **Overall MTCT risk was at least 12.62% (95% CIs: 4.47%-20.77%) among maternal infections during gestation ^a^** |  | **At least 50.34%**  **(95% CIs: 3.75% -96.93%)** | - **NA** - **NA** | - **Overall risk of symptomatic neonatal disease: 11.92% (95% CIs: 3.89%-19.95%)**    - **50.34% (95% CIs: 3.75%- 96.93%) symptomatic neonatal disease among intrapartum maternal infections** - **[8]NA** | - **NA** - **NA** |

**Abbreviations:** APFD: antepartum fetal deaths; CHIKV: chikungunya virus; CIs: confidence intervals; GA: gestational age; mo: months; MTCT: mother to child transmission; NA: not available; NR: not reported; PTD: prior to delivery; VT: vertical transmission, wks: weeks

^a^ For the synthesis of MTCT-risk data across cohorts, when individual cohorts did not report the overall risk of MTCT (symptomatic and asymptomatic neonates) and rather reported only the risk for symptomatic neonatal disease, we extrapolated these data for our calculations, and estimated that the risk of MTCT for these individual studies would have been at least the risk of symptomatic disease (most likely higher). *Most cohorts that reported neonatal infections, had reported only symptomatic cases[4, 8, 12].*

REFERENCES FOR SUPPLEMENTARY MATERIAL

1. Lenglet Y, Barau G, Robillard PY, Randrianaivo H, Michault A, Bouveret A, et al. [Chikungunya infection in pregnancy: Evidence for intrauterine infection in pregnant women and vertical transmission in the parturient. Survey of the Reunion Island outbreak]. J Gynecol Obstet Biol Reprod (Paris). 2006;35(6):578-83. PubMed PMID: 17003745.

2. Robillard PY, Boumahni B, Gerardin P, Michault A, Fourmaintraux A, Schuffenecker I, et al. Vertical maternal fetal transmission of the chikungunya virus - Ten cases among 84 pregnant women. Presse Med. 2006;35(5):785-8. doi: Doi 10.1016/S0755-4982(06)74690-5. PubMed PMID: WOS:000237918800011.

3. Ramful D, Carbonnier M, Pasquet M, Bouhmani B, Ghazouani J, Noormahomed T, et al. Mother-to-child transmission of Chikungunya virus infection. Pediatr Infect Dis J. 2007;26(9):811-5. doi: 10.1097/INF.0b013e3180616d4f. PubMed PMID: WOS:000249455800008.

4. Gerardin P, Barau G, Michault A, Bintner M, Randrianaivo H, Choker G, et al. Multidisciplinary prospective study of mother-to-child chikungunya virus infections on the Island of La Reunion. Plos Med. 2008;5(3):413-23. doi: ARTN 060

10.1371/journal.pmed.0050060. PubMed PMID: WOS:000254928900016.

5. Fritel X, Rollot O, Gerardin P, Gauzere BA, Bideault J, Lagarde L, et al. Chikungunya virus infection during pregnancy, Reunion, France, 2006. Emerg Infect Dis. 2010;16(3):418-25. doi: 10.3201/eid1603.091403. PubMed PMID: 20202416; PubMed Central PMCID: PMCPMC3322036.

6. Ramful D, Samperiz S, Fritel X, Michault A, Jaffar-Bandjee MC, Rollot O, et al. Antibody kinetics in infants exposed to Chikungunya virus infection during pregnancy reveals absence of congenital infection. J Infect Dis. 2014;209(11):1726-30. doi: 10.1093/infdis/jit814. PubMed PMID: 24338351.

7. Gerardin P, Samperiz S, Ramful D, Boumahni B, Bintner M, Alessandri JL, et al. Neurocognitive outcome of children exposed to perinatal mother-to-child Chikungunya virus infection: the CHIMERE cohort study on Reunion Island. PLoS Negl Trop Dis. 2014;8(7):e2996. doi: 10.1371/journal.pntd.0002996. PubMed PMID: 25033077; PubMed Central PMCID: PMCPMC4102444.

8. Sissoko D, Malvy D, Giry C, Delmas G, Paquet C, Gabrie P, et al. Outbreak of Chikungunya fever in Mayotte, Comoros archipelago, 2005-2006. T Roy Soc Trop Med H. 2008;102(8):780-6. doi: 10.1016/j.trstmh.2008.02.018. PubMed PMID: WOS:000258201600008.

9. Watanaveeradej V, Endy TP, Simasathien S, Kerdpanich A, Polprasert N, Aree C, et al. Transplacental chikungunya virus antibody kinetics, Thailand. Emerg Infect Dis. 2006;12(11):1770-2. PubMed PMID: WOS:000241573900025.

10. Senanayake MP SS, Vidanage KK, Gunassena S, Lamabadusurlya SP. Vertical transmission in Chikungunya infection. Cylon Med J. 2009;54(2):47-50.

11. Laoprasopwattana K, Suntharasaj T, Petmanee P, Suddeaugrai O, Geater A. Chikungunya and dengue virus infections during pregnancy: seroprevalence, seroincidence and maternal-fetal transmission, southern Thailand, 2009-2010. Epidemiol Infect. 2016;144(2):381-8. doi: 10.1017/S0950268815001065. PubMed PMID: WOS:000368638100020.

12. Torres JR, Falleiros-Arlant LH, Duenas L, Pleitez-Navarrete J, Salgado DM, Brea-Del Castillo J. Congenital and perinatal complications of chikungunya fever: a Latin American experience. Int J Infect Dis. 2016;51:85-8. doi: 10.1016/j.ijid.2016.09.009. PubMed PMID: WOS:000388326700020.

13. Escobar M, Nieto AJ, Loaiza-Osorio S, Barona JS, Rosso F. Pregnant Women Hospitalized with Chikungunya Virus Infection, Colombia, 2015. Emerg Infect Dis. 2017;23(11):1777-83. doi: 10.3201/eid2311.170480. PubMed PMID: WOS:000413109500002.

1. Lenglet Y, Barau G, Robillard PY, Randrianaivo H, Michault A, Bouveret A, et al. [Chikungunya infection in pregnancy: Evidence for intrauterine infection in pregnant women and vertical transmission in the parturient. Survey of the Reunion Island outbreak]. J Gynecol Obstet Biol Reprod (Paris). 2006;35(6):578-83. PubMed PMID: 17003745.

2. Robillard PY, Boumahni B, Gerardin P, Michault A, Fourmaintraux A, Schuffenecker I, et al. Vertical maternal fetal transmission of the chikungunya virus - Ten cases among 84 pregnant women. Presse Med. 2006;35(5):785-8. doi: Doi 10.1016/S0755-4982(06)74690-5. PubMed PMID: WOS:000237918800011.

3. Ramful D, Carbonnier M, Pasquet M, Bouhmani B, Ghazouani J, Noormahomed T, et al. Mother-to-child transmission of Chikungunya virus infection. Pediatr Infect Dis J. 2007;26(9):811-5. doi: 10.1097/INF.0b013e3180616d4f. PubMed PMID: WOS:000249455800008.

4. Gerardin P, Barau G, Michault A, Bintner M, Randrianaivo H, Choker G, et al. Multidisciplinary prospective study of mother-to-child chikungunya virus infections on the Island of La Reunion. Plos Med. 2008;5(3):413-23. doi: ARTN 060

10.1371/journal.pmed.0050060. PubMed PMID: WOS:000254928900016.

5. Fritel X, Rollot O, Gerardin P, Gauzere BA, Bideault J, Lagarde L, et al. Chikungunya virus infection during pregnancy, Reunion, France, 2006. Emerg Infect Dis. 2010;16(3):418-25. doi: 10.3201/eid1603.091403. PubMed PMID: 20202416; PubMed Central PMCID: PMCPMC3322036.

6. Ramful D, Samperiz S, Fritel X, Michault A, Jaffar-Bandjee MC, Rollot O, et al. Antibody kinetics in infants exposed to Chikungunya virus infection during pregnancy reveals absence of congenital infection. J Infect Dis. 2014;209(11):1726-30. doi: 10.1093/infdis/jit814. PubMed PMID: 24338351.

7. Gerardin P, Samperiz S, Ramful D, Boumahni B, Bintner M, Alessandri JL, et al. Neurocognitive outcome of children exposed to perinatal mother-to-child Chikungunya virus infection: the CHIMERE cohort study on Reunion Island. PLoS Negl Trop Dis. 2014;8(7):e2996. doi: 10.1371/journal.pntd.0002996. PubMed PMID: 25033077; PubMed Central PMCID: PMCPMC4102444.

8. Sissoko D, Malvy D, Giry C, Delmas G, Paquet C, Gabrie P, et al. Outbreak of Chikungunya fever in Mayotte, Comoros archipelago, 2005-2006. T Roy Soc Trop Med H. 2008;102(8):780-6. doi: 10.1016/j.trstmh.2008.02.018. PubMed PMID: WOS:000258201600008.

9. Watanaveeradej V, Endy TP, Simasathien S, Kerdpanich A, Polprasert N, Aree C, et al. Transplacental chikungunya virus antibody kinetics, Thailand. Emerg Infect Dis. 2006;12(11):1770-2. PubMed PMID: WOS:000241573900025.

10. Senanayake MP SS, Vidanage KK, Gunassena S, Lamabadusurlya SP. Vertical transmission in Chikungunya infection. Cylon Med J. 2009;54(2):47-50.

11. Laoprasopwattana K, Suntharasaj T, Petmanee P, Suddeaugrai O, Geater A. Chikungunya and dengue virus infections during pregnancy: seroprevalence, seroincidence and maternal-fetal transmission, southern Thailand, 2009-2010. Epidemiol Infect. 2016;144(2):381-8. doi: 10.1017/S0950268815001065. PubMed PMID: WOS:000368638100020.

12. Torres JR, Falleiros-Arlant LH, Duenas L, Pleitez-Navarrete J, Salgado DM, Brea-Del Castillo J. Congenital and perinatal complications of chikungunya fever: a Latin American experience. Int J Infect Dis. 2016;51:85-8. doi: 10.1016/j.ijid.2016.09.009. PubMed PMID: WOS:000388326700020.

13. Escobar M, Nieto AJ, Loaiza-Osorio S, Barona JS, Rosso F. Pregnant Women Hospitalized with Chikungunya Virus Infection, Colombia, 2015. Emerg Infect Dis. 2017;23(11):1777-83. doi: 10.3201/eid2311.170480. PubMed PMID: WOS:000413109500002.

14. Touret Y, Randrianaivo H, Michault A, Schuffenecker I, Kauffmann E, Lenglet Y, et al. Early maternal-fetal transmission of the Chikungunya virus. Presse Med. 2006;35(11):1656-8. doi: Doi 10.1016/S0755-4982(06)74874-6. PubMed PMID: WOS:000242164400010.

15. Robin S, Rainful D, Le Seach F, Jaffar-Bandjee MC, Rigou G, Alessandri JL. Neurologic manifestations of pediatric chikungunya infection. J Child Neurol. 2008;23(9):1028-35. doi: 10.1177/0883073808314151. PubMed PMID: WOS:000258841800007.

16. Gerardin P, Couderc T, Randrianaivo H, Fritel X, Lecuit M. CHIKUNGUNYA VIRUS-ASSOCIATED ENCEPHALITIS: A COHORT STUDY ON LA REUNION ISLAND, 2005-2009 Response. Neurology. 2016;86(21):2025-6. PubMed PMID: WOS:000376959900023.

17. Boumahni B, Kaplan C, Clabe A, Randrianaivo H, Lanza F. Maternal-fetal chikungunya infection associated with Bernard-Soulier syndrome. Arch Pediatrie. 2011;18(3):272-5. doi: 10.1016/j.arcped.2010.12.002. PubMed PMID: WOS:000288186400006.

18. Alvarado-Socarras JL, Ocampo-Gonzalez M, Vargas-Soler JA, Rodriguez-Morales AJ, Franco-Paredes C. Congenital and Neonatal Chikungunya in Colombia. J Pediatr Infect Dis. 2016;5(3):E17-E20. doi: 10.1093/jpids/piw021. PubMed PMID: WOS:000386138100001.

19. Bandeira AC, Campos GS, Sardi SI, Rocha VFD, Rocha GCM. Neonatal encephalitis due to Chikungunya vertical transmission: First report in Brazil. IDCases. 2016;5:57-9. doi: 10.1016/j.idcr.2016.07.008. PubMed PMID: WOS:000399150800019.

20. Evans-Gilbert T. Case Report: Chikungunya and Neonatal Immunity: Fatal Vertically Transmitted Chikungunya Infection. Am J Trop Med Hyg. 2017;96(4):913-5. doi: 10.4269/ajtmh.16-0491. PubMed PMID: WOS:000401763000027.

21. Karthiga V, Kommu PPK, Krishnan L. Perinatal chikungunya in twins. J Pediatr Neurosci. 2016;11(3):223-4. doi: 10.4103/1817-1745.193369. PubMed PMID: WOS:000390115700012.

22. Khandelwal K, Aara N, Ghiya BC, Bumb RA, Satoskar AR. Centro-Facial Pigmentation in Asymptomatic Congenital Chikungunya Viral Infection. J Paediatr Child H. 2012;48(6):542-3. doi: 10.1111/j.1440-1754.2012.02484.x. PubMed PMID: WOS:000305186200021.

23. Kumar N, Gupta V, Thomas N. Brownie-nose: Hyperpigmentation in Neonatal Chikungunya. Indian Pediatr. 2014;51(5):419-. PubMed PMID: WOS:000336049800023.

24. Lyra PPR, Campos GS, Bandeira ID, Sardi SI, Costa LFD, Santos FR, et al. Congenital Chikungunya Virus Infection after an Outbreak in Salvador, Bahia, Brazil. Ajp Rep. 2016;6(3):E299-E300. doi: 10.1055/s-0036-1587323. PubMed PMID: WOS:000382531200008.

25. Passi GR, Khan YZ, Chitnis DS. Chikungunya infection in neonates. Indian Pediatr. 2008;45(3):240-2. PubMed PMID: WOS:000254357300016.

26. Boumahni B, Bintner M. [Five-year outcome of mother-to-child transmission of chikungunya virus]. Med Trop (Mars). 2012;72 Spec No:94-6. PubMed PMID: 22693938.

27. Pinzon-Redondo H, Paternina-Caicedo A, Barrios-Redondo K, Zarate-Vergara A, Tirado-Perez I, Fortich R, et al. RISK FACTORS FOR SEVERITY OF CHIKUNGUNYA IN CHILDREN A Prospective Assessment. Pediatr Infect Dis J. 2016;35(6):702-4. doi: 10.1097/Inf.0000000000001135. PubMed PMID: WOS:000379343700024.

28. Shenoy S, Pradeep GCM. Neurodevelopmental Outcome of Neonates with Vertically Transmitted Chikungunya Fever with Encephalopathy. Indian Pediatr. 2012;49(3):238-40. PubMed PMID: WOS:000304110800015.

29. Shrivastava A, Beg MW, Gujrati C, Gopalan N, Rao PVL. Management of a Vertically Transmitted Neonatal Chikungunya Thrombocytopenia. Indian J Pediatr. 2011;78(8):1008-9. doi: 10.1007/s12098-011-0371-7. PubMed PMID: WOS:000293143700015.

30. Valamparampil JJ, Chirakkarot S, Letha S, Jayakumar C, Gopinathan KM. Clinical profile of Chikungunya in infants. Indian J Pediatr. 2009;76(2):151-5. doi: 10.1007/s12098-009-0045-x. PubMed PMID: WOS:000264631100003.

31. Vasani R, Kanhere S, Chaudhari K, Phadke V, Mukherjee P, Gupta S, et al. Congenital Chikungunya-A Cause of Neonatal Hyperpigmentation. Pediatr Dermatol. 2016;33(2):209-12. doi: 10.1111/pde.12650. PubMed PMID: WOS:000373067800055.

32. Villamil-Gomez W, Alba-Silvera L, Menco-Ramos A, Gonzalez-Vergara A, Molinares-Palacios T, Barrios-Corrales M, et al. Congenital Chikungunya Virus Infection in Sincelejo, Colombia: A Case Series. J Trop Pediatrics. 2015;61(5):386-92. doi: 10.1093/tropej/fmv051. PubMed PMID: WOS:000365384300010.

33. Rodriguez-Nieves M, Garcia-Garcia I, Garcia-Fragoso L. Perinatally Acquired Chikungunya Infection: The Puerto Rico Experience. Pediatr Infect Dis J. 2016;35(10):1163. doi: 10.1097/INF.0000000000001261. PubMed PMID: 27622689.

34. Gopakumar H, Ramachandran S. Congenital chikungunya. J Clin Neonatol. 2012;1(3):155-6. doi: 10.4103/2249-4847.101704. PubMed PMID: 24027715; PubMed Central PMCID: PMCPMC3762016.

1. Lenglet Y, Barau G, Robillard PY, Randrianaivo H, Michault A, Bouveret A, et al. [Chikungunya infection in pregnancy: Evidence for intrauterine infection in pregnant women and vertical transmission in the parturient. Survey of the Reunion Island outbreak]. J Gynecol Obstet Biol Reprod (Paris). 2006;35(6):578-83. PubMed PMID: 17003745.

2. Robillard PY, Boumahni B, Gerardin P, Michault A, Fourmaintraux A, Schuffenecker I, et al. Vertical maternal fetal transmission of the chikungunya virus - Ten cases among 84 pregnant women. Presse Med. 2006;35(5):785-8. doi: Doi 10.1016/S0755-4982(06)74690-5. PubMed PMID: WOS:000237918800011.

3. Ramful D, Carbonnier M, Pasquet M, Bouhmani B, Ghazouani J, Noormahomed T, et al. Mother-to-child transmission of Chikungunya virus infection. Pediatr Infect Dis J. 2007;26(9):811-5. doi: 10.1097/INF.0b013e3180616d4f. PubMed PMID: WOS:000249455800008.

4. Gerardin P, Barau G, Michault A, Bintner M, Randrianaivo H, Choker G, et al. Multidisciplinary prospective study of mother-to-child chikungunya virus infections on the Island of La Reunion. Plos Med. 2008;5(3):413-23. doi: ARTN 060

10.1371/journal.pmed.0050060. PubMed PMID: WOS:000254928900016.

5. Fritel X, Rollot O, Gerardin P, Gauzere BA, Bideault J, Lagarde L, et al. Chikungunya virus infection during pregnancy, Reunion, France, 2006. Emerg Infect Dis. 2010;16(3):418-25. doi: 10.3201/eid1603.091403. PubMed PMID: 20202416; PubMed Central PMCID: PMCPMC3322036.

6. Ramful D, Samperiz S, Fritel X, Michault A, Jaffar-Bandjee MC, Rollot O, et al. Antibody kinetics in infants exposed to Chikungunya virus infection during pregnancy reveals absence of congenital infection. J Infect Dis. 2014;209(11):1726-30. doi: 10.1093/infdis/jit814. PubMed PMID: 24338351.

7. Gerardin P, Samperiz S, Ramful D, Boumahni B, Bintner M, Alessandri JL, et al. Neurocognitive outcome of children exposed to perinatal mother-to-child Chikungunya virus infection: the CHIMERE cohort study on Reunion Island. PLoS Negl Trop Dis. 2014;8(7):e2996. doi: 10.1371/journal.pntd.0002996. PubMed PMID: 25033077; PubMed Central PMCID: PMCPMC4102444.

8. Sissoko D, Malvy D, Giry C, Delmas G, Paquet C, Gabrie P, et al. Outbreak of Chikungunya fever in Mayotte, Comoros archipelago, 2005-2006. T Roy Soc Trop Med H. 2008;102(8):780-6. doi: 10.1016/j.trstmh.2008.02.018. PubMed PMID: WOS:000258201600008.

9. Watanaveeradej V, Endy TP, Simasathien S, Kerdpanich A, Polprasert N, Aree C, et al. Transplacental chikungunya virus antibody kinetics, Thailand. Emerg Infect Dis. 2006;12(11):1770-2. PubMed PMID: WOS:000241573900025.

10. Senanayake MP SS, Vidanage KK, Gunassena S, Lamabadusurlya SP. Vertical transmission in Chikungunya infection. Cylon Med J. 2009;54(2):47-50.

11. Laoprasopwattana K, Suntharasaj T, Petmanee P, Suddeaugrai O, Geater A. Chikungunya and dengue virus infections during pregnancy: seroprevalence, seroincidence and maternal-fetal transmission, southern Thailand, 2009-2010. Epidemiol Infect. 2016;144(2):381-8. doi: 10.1017/S0950268815001065. PubMed PMID: WOS:000368638100020.

12. Torres JR, Falleiros-Arlant LH, Duenas L, Pleitez-Navarrete J, Salgado DM, Brea-Del Castillo J. Congenital and perinatal complications of chikungunya fever: a Latin American experience. Int J Infect Dis. 2016;51:85-8. doi: 10.1016/j.ijid.2016.09.009. PubMed PMID: WOS:000388326700020.

13. Escobar M, Nieto AJ, Loaiza-Osorio S, Barona JS, Rosso F. Pregnant Women Hospitalized with Chikungunya Virus Infection, Colombia, 2015. Emerg Infect Dis. 2017;23(11):1777-83. doi: 10.3201/eid2311.170480. PubMed PMID: WOS:000413109500002.

14. Touret Y, Randrianaivo H, Michault A, Schuffenecker I, Kauffmann E, Lenglet Y, et al. Early maternal-fetal transmission of the Chikungunya virus. Presse Med. 2006;35(11):1656-8. doi: Doi 10.1016/S0755-4982(06)74874-6. PubMed PMID: WOS:000242164400010.

15. Robin S, Rainful D, Le Seach F, Jaffar-Bandjee MC, Rigou G, Alessandri JL. Neurologic manifestations of pediatric chikungunya infection. J Child Neurol. 2008;23(9):1028-35. doi: 10.1177/0883073808314151. PubMed PMID: WOS:000258841800007.

16. Gerardin P, Couderc T, Randrianaivo H, Fritel X, Lecuit M. CHIKUNGUNYA VIRUS-ASSOCIATED ENCEPHALITIS: A COHORT STUDY ON LA REUNION ISLAND, 2005-2009 Response. Neurology. 2016;86(21):2025-6. PubMed PMID: WOS:000376959900023.

17. Boumahni B, Kaplan C, Clabe A, Randrianaivo H, Lanza F. Maternal-fetal chikungunya infection associated with Bernard-Soulier syndrome. Arch Pediatrie. 2011;18(3):272-5. doi: 10.1016/j.arcped.2010.12.002. PubMed PMID: WOS:000288186400006.

18. Alvarado-Socarras JL, Ocampo-Gonzalez M, Vargas-Soler JA, Rodriguez-Morales AJ, Franco-Paredes C. Congenital and Neonatal Chikungunya in Colombia. J Pediatr Infect Dis. 2016;5(3):E17-E20. doi: 10.1093/jpids/piw021. PubMed PMID: WOS:000386138100001.

19. Bandeira AC, Campos GS, Sardi SI, Rocha VFD, Rocha GCM. Neonatal encephalitis due to Chikungunya vertical transmission: First report in Brazil. IDCases. 2016;5:57-9. doi: 10.1016/j.idcr.2016.07.008. PubMed PMID: WOS:000399150800019.

20. Evans-Gilbert T. Case Report: Chikungunya and Neonatal Immunity: Fatal Vertically Transmitted Chikungunya Infection. Am J Trop Med Hyg. 2017;96(4):913-5. doi: 10.4269/ajtmh.16-0491. PubMed PMID: WOS:000401763000027.

21. Karthiga V, Kommu PPK, Krishnan L. Perinatal chikungunya in twins. J Pediatr Neurosci. 2016;11(3):223-4. doi: 10.4103/1817-1745.193369. PubMed PMID: WOS:000390115700012.

22. Khandelwal K, Aara N, Ghiya BC, Bumb RA, Satoskar AR. Centro-Facial Pigmentation in Asymptomatic Congenital Chikungunya Viral Infection. J Paediatr Child H. 2012;48(6):542-3. doi: 10.1111/j.1440-1754.2012.02484.x. PubMed PMID: WOS:000305186200021.

23. Kumar N, Gupta V, Thomas N. Brownie-nose: Hyperpigmentation in Neonatal Chikungunya. Indian Pediatr. 2014;51(5):419-. PubMed PMID: WOS:000336049800023.

24. Lyra PPR, Campos GS, Bandeira ID, Sardi SI, Costa LFD, Santos FR, et al. Congenital Chikungunya Virus Infection after an Outbreak in Salvador, Bahia, Brazil. Ajp Rep. 2016;6(3):E299-E300. doi: 10.1055/s-0036-1587323. PubMed PMID: WOS:000382531200008.

25. Passi GR, Khan YZ, Chitnis DS. Chikungunya infection in neonates. Indian Pediatr. 2008;45(3):240-2. PubMed PMID: WOS:000254357300016.

26. Boumahni B, Bintner M. [Five-year outcome of mother-to-child transmission of chikungunya virus]. Med Trop (Mars). 2012;72 Spec No:94-6. PubMed PMID: 22693938.

27. Pinzon-Redondo H, Paternina-Caicedo A, Barrios-Redondo K, Zarate-Vergara A, Tirado-Perez I, Fortich R, et al. RISK FACTORS FOR SEVERITY OF CHIKUNGUNYA IN CHILDREN A Prospective Assessment. Pediatr Infect Dis J. 2016;35(6):702-4. doi: 10.1097/Inf.0000000000001135. PubMed PMID: WOS:000379343700024.

28. Shenoy S, Pradeep GCM. Neurodevelopmental Outcome of Neonates with Vertically Transmitted Chikungunya Fever with Encephalopathy. Indian Pediatr. 2012;49(3):238-40. PubMed PMID: WOS:000304110800015.

29. Shrivastava A, Beg MW, Gujrati C, Gopalan N, Rao PVL. Management of a Vertically Transmitted Neonatal Chikungunya Thrombocytopenia. Indian J Pediatr. 2011;78(8):1008-9. doi: 10.1007/s12098-011-0371-7. PubMed PMID: WOS:000293143700015.

30. Valamparampil JJ, Chirakkarot S, Letha S, Jayakumar C, Gopinathan KM. Clinical profile of Chikungunya in infants. Indian J Pediatr. 2009;76(2):151-5. doi: 10.1007/s12098-009-0045-x. PubMed PMID: WOS:000264631100003.

31. Vasani R, Kanhere S, Chaudhari K, Phadke V, Mukherjee P, Gupta S, et al. Congenital Chikungunya-A Cause of Neonatal Hyperpigmentation. Pediatr Dermatol. 2016;33(2):209-12. doi: 10.1111/pde.12650. PubMed PMID: WOS:000373067800055.

32. Villamil-Gomez W, Alba-Silvera L, Menco-Ramos A, Gonzalez-Vergara A, Molinares-Palacios T, Barrios-Corrales M, et al. Congenital Chikungunya Virus Infection in Sincelejo, Colombia: A Case Series. J Trop Pediatrics. 2015;61(5):386-92. doi: 10.1093/tropej/fmv051. PubMed PMID: WOS:000365384300010.

33. Rodriguez-Nieves M, Garcia-Garcia I, Garcia-Fragoso L. Perinatally Acquired Chikungunya Infection: The Puerto Rico Experience. Pediatr Infect Dis J. 2016;35(10):1163. doi: 10.1097/INF.0000000000001261. PubMed PMID: 27622689.

34. Gopakumar H, Ramachandran S. Congenital chikungunya. J Clin Neonatol. 2012;1(3):155-6. doi: 10.4103/2249-4847.101704. PubMed PMID: 24027715; PubMed Central PMCID: PMCPMC3762016.
