# Supplementary material for: Mother-to-child transmission of Chikungunya virus: A systematic review and meta-analysis
Source: PLoS Negl Trop Dis. 2018 Jun 13;12(6):e0006510. doi: 10.1371/journal.pntd.0006510 (PMC6075784; doi:10.1371/journal.pntd.0006510)
Supplement: S4 Table — (DOCX) [file pntd.0006510.s004.docx]

**S4 Table:** Data in Analyses [1-13]

| **N^a^** | **MTCT-risk (Author, Year)** | **Outbreak** | **Percentage** | **Events** | **Maternal Infections** | **MTCT-risk (%)^b^**  **(by extrapolation of data for symptomatic neonatal disease risk for those cohorts not reporting the total number of neonatal infections) (by simple pooling) (%)** | **Total Cases** | **Total Maternal Infections** |
| --- | --- | --- | --- | --- | --- | --- | --- | --- |
|  | Lenglet 2006 | La Reunion | 10.60% | 16 | 151 | **15.48%** | **206** | **1331** |
|  | Ramful 2007 | La Reunion (by data extrapolation from symptomatic neonatal cases: at least this MTCT-risk) | 6.43% | 38 | 591 |  |  |  |
|  | Robillard 2006 | La Reunion (by data extrapolation from symptomatic neonatal cases: at least this MTCT-risk) | 11.9% | 10 | 84 |  |  |  |
|  | Gerardin 2008 | La Reunion | 3.21% | 19 | 591 |  |  |  |
|  | Gerardin 2014 | La Reunion (by data extrapolation from symptomatic neonatal cases: at least this MTCT-risk) | 6.26% | 37 | 591 |  |  |  |
| 1 | La Reunion-overall | La Reunion (by data extrapolation from symptomatic neonatal cases: at least this MTCT-risk) | 6.43% | 38 | 591 |  |  |  |
| 2 | Watanaveeradej 2006 | Thailand | 0.00% | 0 | 28 |  |  |  |
| 3 | Sissoko 2008 | Mayotte (by data extrapolation from symptomatic neonatal cases: at least this MTCT-risk) | 5.52% | 9 | 163 |  |  |  |
| 4 | Senanayake 2009 | Shri Lanka | 14.00% | 7 | 50 |  |  |  |
| 5 | Laoprasopwattan 2016 | Thailand | 0.00% | 0 | 88 |  |  |  |
| 6 | Torres 2016 | El Salvador (by data extrapolation from symptomatic neonatal cases: at least this MTCT-risk) | 27.75% | 53 | 191 |  |  |  |
| 7 | Torres 2016 | Santo Domingo (by data extrapolation from symptomatic neonatal cases: at least this MTCT-risk) | 48.29% | 99 | 205 |  |  |  |
| 8 | Escobar 2017 | Colombia (analyzed only 12 periopartum+3 intrapartum maternal infections) | 0.00% | 0 | 15 |  |  |  |
|  | **MTCT from anterpartum/peripartum maternal infections** | **Outbreak** | **Percentage** | **Events** | **Maternal Infections** | **MTCT-risk from ante/peripartum infections (by simple pooling) (%)** | **Total Cases** | **Total Antepartum/Peripartum Maternal Infections** |
|  | Lenglet 2006 | La Reunion | 0% | 0 | 116 | **0%** | **0** | **712** |
| 1 | Geradin 2008 | La Reunion | 0% | 0 | 700 |  |  |  |
| 2 | **Escobar 2017** | Colombia | 0% | 0 | 12 |  |  |  |
|  | **MTCT from intrapartum maternal infections** | **Outbreak** | **Percentage** | **Events** | **Maternal Infections** | **MTCT-risk from intrapartum infections (by simple pooling) (%)** | **Total Cases** | **Total Intrapartum Maternal Infections** |
|  | Lenglet 2006 | La Reunion | 48.48% | 16 | 33 | **50.00%** | **23** | **46** |
|  | Geradin 2008 | La Reunion | 48.72% | 19 | 39 |  |  |  |
|  | Fritel 2010 | La Reunion | 25.00% | 1 | 4 |  |  |  |
| 1 | La Reunion-overall | La Reunion | 48.72% | 19 | 39 |  |  |  |
| 2 | Senanayake 2009 | Shri Lanka | 100.00% | 4 | 4 |  |  |  |
| 3 | Escobar 2017 | Colombia | 0.00% | 0 | 3 |  |  |  |
|  | **APFD-risk (Author, Year)** | **Outbreak** | **Percentage** | **Events** | **Maternal Infections** | **APFD-risk (%)**  **(by simple pooling) (%)** | **Total Cases** | **Total Maternal Infections** |
|  | Lenglet 2006 | La Reunion | 7.28% | 11 | 151 | **1.66%** | **20** | **1203** |
|  | Gerardin 2008 | La Reunion | 2.17% | 16 | 739 |  |  |  |
|  | Fritel 2010 | La Reunion | 1.52% | 10 | 656 |  |  |  |
| 1 | La Reunion-overall | La Reunion | 2.17% | 16 | 739 |  |  |  |
| 2 | Sissoko 2008 | Mayotte | 0.00% | 0 | 163 |  |  |  |
| 3 | Senanayake 2009 | Shri Lanka | 6.00% | 3 | 50 |  |  |  |
| 4 | Torres 2016 | El Salvador | 0.00% | 0 | 191 |  |  |  |
| 5 | Escobar 2017 | Colombia | 1.67% | 1 | 60 |  |  |  |
|  | **CHIKV-confirmed APFDs** | **Outbreak** | **Percentage** | **Events** | **Maternal Infections** | **CHIKV-confirmed-APFD-risk (%)**  **(by simple pooling) (%)** | **Total Cases** | **Total Maternal Infections** |
|  | Lenglet 2006 | La Reunion | 1.99% | 3 | 151 | **0.25%** | **3** | **1203** |
|  | Gerardin 2008 | La Reunion | 0.41% | 3 | 739 |  |  |  |
|  | Fritel 2010 | La Reunion | 0.46% | 3 | 656 |  |  |  |
| 1 | La Reunion-Overall | La Reunion | 0.41% | 3 | 739 |  |  |  |
| 2 | Sissoko 2008 | Mayotte | 0.00% | 0 | 163 |  |  |  |
| 3 | Senanayake 2009 | Shri Lanka | 0.00% | 0 | 50 |  |  |  |
| 4 | Torres 2016 | El Salvador | 0.00% | 0 | 191 |  |  |  |
| 5 | Escobar 2017 | Colombia | 0.00% | 0 | 60 |  |  |  |
|  | **Symptomatic Neonatal Disease risk from maternal infection during gestation (Author, Year)** | **Outbreak** | **Percentage** | **Events** | **Maternal Infections** | **Symptomatic neonatal disease risk**  **(by simple pooling) (%)** | **Total Cases** | **Total Maternal Infections** |
|  | Lenglet 2006 | La Reunion | 10.60% | 16 | 151 | **15.25%** | **203** | **1331** |
|  | Robillard 2006 | La Reunion (severe neonatal disease) | 11.90% | 10 | 84 |  |  |  |
|  | Ramful 2007 | La Reunion (by data extrapolation to the 591 cohort-maternal infections) | 6.43% | 38 | 591 |  |  |  |
|  | Gerardin 2008 | La Reunion | 3.21% | 19 | 591 |  |  |  |
|  | Gerardin 2014 | La Reunion | 6.26% | 37 | 591 |  |  |  |
| 1 | La Reunion-overall | La Reunion | 6.43% | 38 | 591 |  |  |  |
| 2 | Watanaveeradej 2006 | Thailand | 0.00% | 0 | 28 |  |  |  |
| 3 | Sissoko 2008 | Mayotte | 5.52% | 9 | 163 |  |  |  |
| 4 | Senanayake 2009 | Shri Lanka (severe neonatal disease risk) | 8.00% | 4 | 50 |  |  |  |
| 5 | Laoprasopwattan 2016 | Thailand | 0.00% | 0 | 88 |  |  |  |
| 6 | Torres 2016 | El Salvador | 27.75% | 53 | 191 |  |  |  |
| 7 | Torres 2016 | Santo Domingo | 48.29% | 99 | 205 |  |  |  |
| 8 | Escobar 2017 | Colombia (analyzed only 12 periopartum+3 intrapartum maternal infections) | 0.00% | 0 | 15 |  |  |  |
|  | **Symptomatic neonatal disease from intrapartum maternal infections (Author, Year)** | **Outbreak** | **Percentage** | **Events** | **Maternal Infections during intrapartum period** | **Symptomatic Neonatal Disease risk-from intrapartum infections (by simple pooling) (%** | **Total Cases** | **Total Intrapartum Maternal Infections** |
|  | Lenglet 2006 | La Reunion | 48.48% | 16 | 33 | **50.00%** | **23** | **46** |
|  | Robillard 2006 | La Reunion | 100.00% | 10 | 10 |  |  |  |
| 1 | Gerardin 2008 | La Reunion | 48.72% | 19 | 39 |  |  |  |
| 2 | Senanayake 2009 | Shri Lanka (severely symptomatic neonates) | 100.00% | 4 | 4 |  |  |  |
| 3 | Escobar 2017 | Colombia | 0.00% | 0 | 3 |  |  |  |
|  | **Symptomatic neonatal disease from antepartum/peripartum maternal infections (Author, Year)** | **Outbreak** | **Percentage** | **Events** | **Maternal Infections during intrapartum period** | **Symptomatic Neonatal Disease risk-from intrapartum infections (by simple pooling) (%)** | **Total Cases** | **Total Antepartum/Peripartum Maternal Infections** |
|  | Lenglet 2006 | La Reunion | 0.00% | 0 | 116 | **0%** | **0** | **758** |
|  | Robillard 2006 | La Reunion | 0.00% | 0 | 74 |  |  |  |
| 1 | Gerardin 2008 | La Reunion | 0.00% | 0 | 700 |  |  |  |
| 2 | Senanayake 2009 | Shri Lanka (severely symptomatic neonates) | 0.00% | 0 | 46 |  |  |  |
| 3 | Escobar 2017 | Colombia | 0.00% | 0 | 12 |  |  |  |
|  | **Neonatal Mortality (Author, Year)** | **Outbreak** | **Percentage** | **Events** | **Maternal Infections** | **Neonatal Mortality Risk-among maternal infections (by simple pooling) (%)** | **Neonatal deaths** | **Maternal Infections** |
|  | Robillard 2006 | La Reunion | 0.00% | 0 | 84 | **0.60%** | **5** | **832** |
|  | Ramful 2007 | La Reunion | 0.17% | 1 | 591 |  |  |  |
| 1 | La Reunion-Overall | La Reunion | 0.17% | 1 | 591 |  |  |  |
| 2 | Senanayake 2009 | Shri Lanka (severely symptomatic neonates) | 0.00% | 0 | 50 |  |  |  |
| 3 | Torres 2016 | Santo Domingo | 2.09% | 4 | 191 |  |  |  |
|  | **Neonatal Mortality (Author, Year)** | **Outbreak** | **Percentage** | **Events** | **Neonatal Infections** | **Neonatal Mortality Risk-among neonatal infections (by simple pooling) (%)** | **Neonatal deaths** | **Neonatal Infections** |
|  | Ramful 2007 | La Reunion | 2.63% | 1 | 38 | **2.75%** | **5** | **182** |
| 1 | La Reunion-Overall | La Reunion | 1.19% | 1 | 84 |  |  |  |
| 2 | Senanayake 2009 | Shri Lanka | 0.00% | 0 | 19 |  |  |  |
| 3 | Torres 2016 | Santo Domingo | 5.06% | 4 | 79 |  |  |  |

**Footnotes:**

^a^ For cohorts with multiple publications, we included in the pooled analyses only data from the articles numbered in the first column N

^b^ Risks are estimated by simple pooling across cohorts. Only non-overlapping data were kept from cohorts with multiple publications, considering the total number of pertinent outcomes for all maternal infections in each cohort.

REFERENCES FOR SUPPLEMENTARY MATERIAL

1. Lenglet Y, Barau G, Robillard PY, Randrianaivo H, Michault A, Bouveret A, et al. [Chikungunya infection in pregnancy: Evidence for intrauterine infection in pregnant women and vertical transmission in the parturient. Survey of the Reunion Island outbreak]. J Gynecol Obstet Biol Reprod (Paris). 2006;35(6):578-83. PubMed PMID: 17003745.

2. Robillard PY, Boumahni B, Gerardin P, Michault A, Fourmaintraux A, Schuffenecker I, et al. Vertical maternal fetal transmission of the chikungunya virus - Ten cases among 84 pregnant women. Presse Med. 2006;35(5):785-8. doi: Doi 10.1016/S0755-4982(06)74690-5. PubMed PMID: WOS:000237918800011.

3. Ramful D, Carbonnier M, Pasquet M, Bouhmani B, Ghazouani J, Noormahomed T, et al. Mother-to-child transmission of Chikungunya virus infection. Pediatr Infect Dis J. 2007;26(9):811-5. doi: 10.1097/INF.0b013e3180616d4f. PubMed PMID: WOS:000249455800008.

4. Gerardin P, Barau G, Michault A, Bintner M, Randrianaivo H, Choker G, et al. Multidisciplinary prospective study of mother-to-child chikungunya virus infections on the Island of La Reunion. Plos Med. 2008;5(3):413-23. doi: ARTN 060

10.1371/journal.pmed.0050060. PubMed PMID: WOS:000254928900016.

5. Fritel X, Rollot O, Gerardin P, Gauzere BA, Bideault J, Lagarde L, et al. Chikungunya virus infection during pregnancy, Reunion, France, 2006. Emerg Infect Dis. 2010;16(3):418-25. doi: 10.3201/eid1603.091403. PubMed PMID: 20202416; PubMed Central PMCID: PMCPMC3322036.

6. Ramful D, Samperiz S, Fritel X, Michault A, Jaffar-Bandjee MC, Rollot O, et al. Antibody kinetics in infants exposed to Chikungunya virus infection during pregnancy reveals absence of congenital infection. J Infect Dis. 2014;209(11):1726-30. doi: 10.1093/infdis/jit814. PubMed PMID: 24338351.

7. Gerardin P, Samperiz S, Ramful D, Boumahni B, Bintner M, Alessandri JL, et al. Neurocognitive outcome of children exposed to perinatal mother-to-child Chikungunya virus infection: the CHIMERE cohort study on Reunion Island. PLoS Negl Trop Dis. 2014;8(7):e2996. doi: 10.1371/journal.pntd.0002996. PubMed PMID: 25033077; PubMed Central PMCID: PMCPMC4102444.

8. Sissoko D, Malvy D, Giry C, Delmas G, Paquet C, Gabrie P, et al. Outbreak of Chikungunya fever in Mayotte, Comoros archipelago, 2005-2006. T Roy Soc Trop Med H. 2008;102(8):780-6. doi: 10.1016/j.trstmh.2008.02.018. PubMed PMID: WOS:000258201600008.

9. Watanaveeradej V, Endy TP, Simasathien S, Kerdpanich A, Polprasert N, Aree C, et al. Transplacental chikungunya virus antibody kinetics, Thailand. Emerg Infect Dis. 2006;12(11):1770-2. PubMed PMID: WOS:000241573900025.

10. Senanayake MP SS, Vidanage KK, Gunassena S, Lamabadusurlya SP. Vertical transmission in Chikungunya infection. Cylon Med J. 2009;54(2):47-50.

11. Laoprasopwattana K, Suntharasaj T, Petmanee P, Suddeaugrai O, Geater A. Chikungunya and dengue virus infections during pregnancy: seroprevalence, seroincidence and maternal-fetal transmission, southern Thailand, 2009-2010. Epidemiol Infect. 2016;144(2):381-8. doi: 10.1017/S0950268815001065. PubMed PMID: WOS:000368638100020.

12. Torres JR, Falleiros-Arlant LH, Duenas L, Pleitez-Navarrete J, Salgado DM, Brea-Del Castillo J. Congenital and perinatal complications of chikungunya fever: a Latin American experience. Int J Infect Dis. 2016;51:85-8. doi: 10.1016/j.ijid.2016.09.009. PubMed PMID: WOS:000388326700020.

13. Escobar M, Nieto AJ, Loaiza-Osorio S, Barona JS, Rosso F. Pregnant Women Hospitalized with Chikungunya Virus Infection, Colombia, 2015. Emerg Infect Dis. 2017;23(11):1777-83. doi: 10.3201/eid2311.170480. PubMed PMID: WOS:000413109500002.

1. Lenglet Y, Barau G, Robillard PY, Randrianaivo H, Michault A, Bouveret A, et al. [Chikungunya infection in pregnancy: Evidence for intrauterine infection in pregnant women and vertical transmission in the parturient. Survey of the Reunion Island outbreak]. J Gynecol Obstet Biol Reprod (Paris). 2006;35(6):578-83. PubMed PMID: 17003745.

2. Robillard PY, Boumahni B, Gerardin P, Michault A, Fourmaintraux A, Schuffenecker I, et al. Vertical maternal fetal transmission of the chikungunya virus - Ten cases among 84 pregnant women. Presse Med. 2006;35(5):785-8. doi: Doi 10.1016/S0755-4982(06)74690-5. PubMed PMID: WOS:000237918800011.

3. Ramful D, Carbonnier M, Pasquet M, Bouhmani B, Ghazouani J, Noormahomed T, et al. Mother-to-child transmission of Chikungunya virus infection. Pediatr Infect Dis J. 2007;26(9):811-5. doi: 10.1097/INF.0b013e3180616d4f. PubMed PMID: WOS:000249455800008.

4. Gerardin P, Barau G, Michault A, Bintner M, Randrianaivo H, Choker G, et al. Multidisciplinary prospective study of mother-to-child chikungunya virus infections on the Island of La Reunion. Plos Med. 2008;5(3):413-23. doi: ARTN 060

10.1371/journal.pmed.0050060. PubMed PMID: WOS:000254928900016.

5. Fritel X, Rollot O, Gerardin P, Gauzere BA, Bideault J, Lagarde L, et al. Chikungunya virus infection during pregnancy, Reunion, France, 2006. Emerg Infect Dis. 2010;16(3):418-25. doi: 10.3201/eid1603.091403. PubMed PMID: 20202416; PubMed Central PMCID: PMCPMC3322036.

6. Ramful D, Samperiz S, Fritel X, Michault A, Jaffar-Bandjee MC, Rollot O, et al. Antibody kinetics in infants exposed to Chikungunya virus infection during pregnancy reveals absence of congenital infection. J Infect Dis. 2014;209(11):1726-30. doi: 10.1093/infdis/jit814. PubMed PMID: 24338351.

7. Gerardin P, Samperiz S, Ramful D, Boumahni B, Bintner M, Alessandri JL, et al. Neurocognitive outcome of children exposed to perinatal mother-to-child Chikungunya virus infection: the CHIMERE cohort study on Reunion Island. PLoS Negl Trop Dis. 2014;8(7):e2996. doi: 10.1371/journal.pntd.0002996. PubMed PMID: 25033077; PubMed Central PMCID: PMCPMC4102444.

8. Sissoko D, Malvy D, Giry C, Delmas G, Paquet C, Gabrie P, et al. Outbreak of Chikungunya fever in Mayotte, Comoros archipelago, 2005-2006. T Roy Soc Trop Med H. 2008;102(8):780-6. doi: 10.1016/j.trstmh.2008.02.018. PubMed PMID: WOS:000258201600008.

9. Watanaveeradej V, Endy TP, Simasathien S, Kerdpanich A, Polprasert N, Aree C, et al. Transplacental chikungunya virus antibody kinetics, Thailand. Emerg Infect Dis. 2006;12(11):1770-2. PubMed PMID: WOS:000241573900025.

10. Senanayake MP SS, Vidanage KK, Gunassena S, Lamabadusurlya SP. Vertical transmission in Chikungunya infection. Cylon Med J. 2009;54(2):47-50.

11. Laoprasopwattana K, Suntharasaj T, Petmanee P, Suddeaugrai O, Geater A. Chikungunya and dengue virus infections during pregnancy: seroprevalence, seroincidence and maternal-fetal transmission, southern Thailand, 2009-2010. Epidemiol Infect. 2016;144(2):381-8. doi: 10.1017/S0950268815001065. PubMed PMID: WOS:000368638100020.

12. Torres JR, Falleiros-Arlant LH, Duenas L, Pleitez-Navarrete J, Salgado DM, Brea-Del Castillo J. Congenital and perinatal complications of chikungunya fever: a Latin American experience. Int J Infect Dis. 2016;51:85-8. doi: 10.1016/j.ijid.2016.09.009. PubMed PMID: WOS:000388326700020.

13. Escobar M, Nieto AJ, Loaiza-Osorio S, Barona JS, Rosso F. Pregnant Women Hospitalized with Chikungunya Virus Infection, Colombia, 2015. Emerg Infect Dis. 2017;23(11):1777-83. doi: 10.3201/eid2311.170480. PubMed PMID: WOS:000413109500002.

14. Touret Y, Randrianaivo H, Michault A, Schuffenecker I, Kauffmann E, Lenglet Y, et al. Early maternal-fetal transmission of the Chikungunya virus. Presse Med. 2006;35(11):1656-8. doi: Doi 10.1016/S0755-4982(06)74874-6. PubMed PMID: WOS:000242164400010.

15. Robin S, Rainful D, Le Seach F, Jaffar-Bandjee MC, Rigou G, Alessandri JL. Neurologic manifestations of pediatric chikungunya infection. J Child Neurol. 2008;23(9):1028-35. doi: 10.1177/0883073808314151. PubMed PMID: WOS:000258841800007.

16. Gerardin P, Couderc T, Randrianaivo H, Fritel X, Lecuit M. CHIKUNGUNYA VIRUS-ASSOCIATED ENCEPHALITIS: A COHORT STUDY ON LA REUNION ISLAND, 2005-2009 Response. Neurology. 2016;86(21):2025-6. PubMed PMID: WOS:000376959900023.

17. Boumahni B, Kaplan C, Clabe A, Randrianaivo H, Lanza F. Maternal-fetal chikungunya infection associated with Bernard-Soulier syndrome. Arch Pediatrie. 2011;18(3):272-5. doi: 10.1016/j.arcped.2010.12.002. PubMed PMID: WOS:000288186400006.

18. Alvarado-Socarras JL, Ocampo-Gonzalez M, Vargas-Soler JA, Rodriguez-Morales AJ, Franco-Paredes C. Congenital and Neonatal Chikungunya in Colombia. J Pediatr Infect Dis. 2016;5(3):E17-E20. doi: 10.1093/jpids/piw021. PubMed PMID: WOS:000386138100001.

19. Bandeira AC, Campos GS, Sardi SI, Rocha VFD, Rocha GCM. Neonatal encephalitis due to Chikungunya vertical transmission: First report in Brazil. IDCases. 2016;5:57-9. doi: 10.1016/j.idcr.2016.07.008. PubMed PMID: WOS:000399150800019.

20. Evans-Gilbert T. Case Report: Chikungunya and Neonatal Immunity: Fatal Vertically Transmitted Chikungunya Infection. Am J Trop Med Hyg. 2017;96(4):913-5. doi: 10.4269/ajtmh.16-0491. PubMed PMID: WOS:000401763000027.

21. Karthiga V, Kommu PPK, Krishnan L. Perinatal chikungunya in twins. J Pediatr Neurosci. 2016;11(3):223-4. doi: 10.4103/1817-1745.193369. PubMed PMID: WOS:000390115700012.

22. Khandelwal K, Aara N, Ghiya BC, Bumb RA, Satoskar AR. Centro-Facial Pigmentation in Asymptomatic Congenital Chikungunya Viral Infection. J Paediatr Child H. 2012;48(6):542-3. doi: 10.1111/j.1440-1754.2012.02484.x. PubMed PMID: WOS:000305186200021.

23. Kumar N, Gupta V, Thomas N. Brownie-nose: Hyperpigmentation in Neonatal Chikungunya. Indian Pediatr. 2014;51(5):419-. PubMed PMID: WOS:000336049800023.

24. Lyra PPR, Campos GS, Bandeira ID, Sardi SI, Costa LFD, Santos FR, et al. Congenital Chikungunya Virus Infection after an Outbreak in Salvador, Bahia, Brazil. Ajp Rep. 2016;6(3):E299-E300. doi: 10.1055/s-0036-1587323. PubMed PMID: WOS:000382531200008.

25. Passi GR, Khan YZ, Chitnis DS. Chikungunya infection in neonates. Indian Pediatr. 2008;45(3):240-2. PubMed PMID: WOS:000254357300016.

26. Boumahni B, Bintner M. [Five-year outcome of mother-to-child transmission of chikungunya virus]. Med Trop (Mars). 2012;72 Spec No:94-6. PubMed PMID: 22693938.

27. Pinzon-Redondo H, Paternina-Caicedo A, Barrios-Redondo K, Zarate-Vergara A, Tirado-Perez I, Fortich R, et al. RISK FACTORS FOR SEVERITY OF CHIKUNGUNYA IN CHILDREN A Prospective Assessment. Pediatr Infect Dis J. 2016;35(6):702-4. doi: 10.1097/Inf.0000000000001135. PubMed PMID: WOS:000379343700024.

28. Shenoy S, Pradeep GCM. Neurodevelopmental Outcome of Neonates with Vertically Transmitted Chikungunya Fever with Encephalopathy. Indian Pediatr. 2012;49(3):238-40. PubMed PMID: WOS:000304110800015.

29. Shrivastava A, Beg MW, Gujrati C, Gopalan N, Rao PVL. Management of a Vertically Transmitted Neonatal Chikungunya Thrombocytopenia. Indian J Pediatr. 2011;78(8):1008-9. doi: 10.1007/s12098-011-0371-7. PubMed PMID: WOS:000293143700015.

30. Valamparampil JJ, Chirakkarot S, Letha S, Jayakumar C, Gopinathan KM. Clinical profile of Chikungunya in infants. Indian J Pediatr. 2009;76(2):151-5. doi: 10.1007/s12098-009-0045-x. PubMed PMID: WOS:000264631100003.

31. Vasani R, Kanhere S, Chaudhari K, Phadke V, Mukherjee P, Gupta S, et al. Congenital Chikungunya-A Cause of Neonatal Hyperpigmentation. Pediatr Dermatol. 2016;33(2):209-12. doi: 10.1111/pde.12650. PubMed PMID: WOS:000373067800055.

32. Villamil-Gomez W, Alba-Silvera L, Menco-Ramos A, Gonzalez-Vergara A, Molinares-Palacios T, Barrios-Corrales M, et al. Congenital Chikungunya Virus Infection in Sincelejo, Colombia: A Case Series. J Trop Pediatrics. 2015;61(5):386-92. doi: 10.1093/tropej/fmv051. PubMed PMID: WOS:000365384300010.

33. Rodriguez-Nieves M, Garcia-Garcia I, Garcia-Fragoso L. Perinatally Acquired Chikungunya Infection: The Puerto Rico Experience. Pediatr Infect Dis J. 2016;35(10):1163. doi: 10.1097/INF.0000000000001261. PubMed PMID: 27622689.

34. Gopakumar H, Ramachandran S. Congenital chikungunya. J Clin Neonatol. 2012;1(3):155-6. doi: 10.4103/2249-4847.101704. PubMed PMID: 24027715; PubMed Central PMCID: PMCPMC3762016.

1. Lenglet Y, Barau G, Robillard PY, Randrianaivo H, Michault A, Bouveret A, et al. [Chikungunya infection in pregnancy: Evidence for intrauterine infection in pregnant women and vertical transmission in the parturient. Survey of the Reunion Island outbreak]. J Gynecol Obstet Biol Reprod (Paris). 2006;35(6):578-83. PubMed PMID: 17003745.

2. Robillard PY, Boumahni B, Gerardin P, Michault A, Fourmaintraux A, Schuffenecker I, et al. Vertical maternal fetal transmission of the chikungunya virus - Ten cases among 84 pregnant women. Presse Med. 2006;35(5):785-8. doi: Doi 10.1016/S0755-4982(06)74690-5. PubMed PMID: WOS:000237918800011.

3. Ramful D, Carbonnier M, Pasquet M, Bouhmani B, Ghazouani J, Noormahomed T, et al. Mother-to-child transmission of Chikungunya virus infection. Pediatr Infect Dis J. 2007;26(9):811-5. doi: 10.1097/INF.0b013e3180616d4f. PubMed PMID: WOS:000249455800008.

4. Gerardin P, Barau G, Michault A, Bintner M, Randrianaivo H, Choker G, et al. Multidisciplinary prospective study of mother-to-child chikungunya virus infections on the Island of La Reunion. Plos Med. 2008;5(3):413-23. doi: ARTN 060

10.1371/journal.pmed.0050060. PubMed PMID: WOS:000254928900016.

5. Fritel X, Rollot O, Gerardin P, Gauzere BA, Bideault J, Lagarde L, et al. Chikungunya virus infection during pregnancy, Reunion, France, 2006. Emerg Infect Dis. 2010;16(3):418-25. doi: 10.3201/eid1603.091403. PubMed PMID: 20202416; PubMed Central PMCID: PMCPMC3322036.

6. Ramful D, Samperiz S, Fritel X, Michault A, Jaffar-Bandjee MC, Rollot O, et al. Antibody kinetics in infants exposed to Chikungunya virus infection during pregnancy reveals absence of congenital infection. J Infect Dis. 2014;209(11):1726-30. doi: 10.1093/infdis/jit814. PubMed PMID: 24338351.

7. Gerardin P, Samperiz S, Ramful D, Boumahni B, Bintner M, Alessandri JL, et al. Neurocognitive outcome of children exposed to perinatal mother-to-child Chikungunya virus infection: the CHIMERE cohort study on Reunion Island. PLoS Negl Trop Dis. 2014;8(7):e2996. doi: 10.1371/journal.pntd.0002996. PubMed PMID: 25033077; PubMed Central PMCID: PMCPMC4102444.

8. Sissoko D, Malvy D, Giry C, Delmas G, Paquet C, Gabrie P, et al. Outbreak of Chikungunya fever in Mayotte, Comoros archipelago, 2005-2006. T Roy Soc Trop Med H. 2008;102(8):780-6. doi: 10.1016/j.trstmh.2008.02.018. PubMed PMID: WOS:000258201600008.

9. Watanaveeradej V, Endy TP, Simasathien S, Kerdpanich A, Polprasert N, Aree C, et al. Transplacental chikungunya virus antibody kinetics, Thailand. Emerg Infect Dis. 2006;12(11):1770-2. PubMed PMID: WOS:000241573900025.

10. Senanayake MP SS, Vidanage KK, Gunassena S, Lamabadusurlya SP. Vertical transmission in Chikungunya infection. Cylon Med J. 2009;54(2):47-50.

11. Laoprasopwattana K, Suntharasaj T, Petmanee P, Suddeaugrai O, Geater A. Chikungunya and dengue virus infections during pregnancy: seroprevalence, seroincidence and maternal-fetal transmission, southern Thailand, 2009-2010. Epidemiol Infect. 2016;144(2):381-8. doi: 10.1017/S0950268815001065. PubMed PMID: WOS:000368638100020.

12. Torres JR, Falleiros-Arlant LH, Duenas L, Pleitez-Navarrete J, Salgado DM, Brea-Del Castillo J. Congenital and perinatal complications of chikungunya fever: a Latin American experience. Int J Infect Dis. 2016;51:85-8. doi: 10.1016/j.ijid.2016.09.009. PubMed PMID: WOS:000388326700020.

13. Escobar M, Nieto AJ, Loaiza-Osorio S, Barona JS, Rosso F. Pregnant Women Hospitalized with Chikungunya Virus Infection, Colombia, 2015. Emerg Infect Dis. 2017;23(11):1777-83. doi: 10.3201/eid2311.170480. PubMed PMID: WOS:000413109500002.

14. Touret Y, Randrianaivo H, Michault A, Schuffenecker I, Kauffmann E, Lenglet Y, et al. Early maternal-fetal transmission of the Chikungunya virus. Presse Med. 2006;35(11):1656-8. doi: Doi 10.1016/S0755-4982(06)74874-6. PubMed PMID: WOS:000242164400010.

15. Robin S, Rainful D, Le Seach F, Jaffar-Bandjee MC, Rigou G, Alessandri JL. Neurologic manifestations of pediatric chikungunya infection. J Child Neurol. 2008;23(9):1028-35. doi: 10.1177/0883073808314151. PubMed PMID: WOS:000258841800007.

16. Gerardin P, Couderc T, Randrianaivo H, Fritel X, Lecuit M. CHIKUNGUNYA VIRUS-ASSOCIATED ENCEPHALITIS: A COHORT STUDY ON LA REUNION ISLAND, 2005-2009 Response. Neurology. 2016;86(21):2025-6. PubMed PMID: WOS:000376959900023.

17. Boumahni B, Kaplan C, Clabe A, Randrianaivo H, Lanza F. Maternal-fetal chikungunya infection associated with Bernard-Soulier syndrome. Arch Pediatrie. 2011;18(3):272-5. doi: 10.1016/j.arcped.2010.12.002. PubMed PMID: WOS:000288186400006.

18. Alvarado-Socarras JL, Ocampo-Gonzalez M, Vargas-Soler JA, Rodriguez-Morales AJ, Franco-Paredes C. Congenital and Neonatal Chikungunya in Colombia. J Pediatr Infect Dis. 2016;5(3):E17-E20. doi: 10.1093/jpids/piw021. PubMed PMID: WOS:000386138100001.

19. Bandeira AC, Campos GS, Sardi SI, Rocha VFD, Rocha GCM. Neonatal encephalitis due to Chikungunya vertical transmission: First report in Brazil. IDCases. 2016;5:57-9. doi: 10.1016/j.idcr.2016.07.008. PubMed PMID: WOS:000399150800019.

20. Evans-Gilbert T. Case Report: Chikungunya and Neonatal Immunity: Fatal Vertically Transmitted Chikungunya Infection. Am J Trop Med Hyg. 2017;96(4):913-5. doi: 10.4269/ajtmh.16-0491. PubMed PMID: WOS:000401763000027.

21. Karthiga V, Kommu PPK, Krishnan L. Perinatal chikungunya in twins. J Pediatr Neurosci. 2016;11(3):223-4. doi: 10.4103/1817-1745.193369. PubMed PMID: WOS:000390115700012.

22. Khandelwal K, Aara N, Ghiya BC, Bumb RA, Satoskar AR. Centro-Facial Pigmentation in Asymptomatic Congenital Chikungunya Viral Infection. J Paediatr Child H. 2012;48(6):542-3. doi: 10.1111/j.1440-1754.2012.02484.x. PubMed PMID: WOS:000305186200021.

23. Kumar N, Gupta V, Thomas N. Brownie-nose: Hyperpigmentation in Neonatal Chikungunya. Indian Pediatr. 2014;51(5):419-. PubMed PMID: WOS:000336049800023.

24. Lyra PPR, Campos GS, Bandeira ID, Sardi SI, Costa LFD, Santos FR, et al. Congenital Chikungunya Virus Infection after an Outbreak in Salvador, Bahia, Brazil. Ajp Rep. 2016;6(3):E299-E300. doi: 10.1055/s-0036-1587323. PubMed PMID: WOS:000382531200008.

25. Passi GR, Khan YZ, Chitnis DS. Chikungunya infection in neonates. Indian Pediatr. 2008;45(3):240-2. PubMed PMID: WOS:000254357300016.

26. Boumahni B, Bintner M. [Five-year outcome of mother-to-child transmission of chikungunya virus]. Med Trop (Mars). 2012;72 Spec No:94-6. PubMed PMID: 22693938.

27. Pinzon-Redondo H, Paternina-Caicedo A, Barrios-Redondo K, Zarate-Vergara A, Tirado-Perez I, Fortich R, et al. RISK FACTORS FOR SEVERITY OF CHIKUNGUNYA IN CHILDREN A Prospective Assessment. Pediatr Infect Dis J. 2016;35(6):702-4. doi: 10.1097/Inf.0000000000001135. PubMed PMID: WOS:000379343700024.

28. Shenoy S, Pradeep GCM. Neurodevelopmental Outcome of Neonates with Vertically Transmitted Chikungunya Fever with Encephalopathy. Indian Pediatr. 2012;49(3):238-40. PubMed PMID: WOS:000304110800015.

29. Shrivastava A, Beg MW, Gujrati C, Gopalan N, Rao PVL. Management of a Vertically Transmitted Neonatal Chikungunya Thrombocytopenia. Indian J Pediatr. 2011;78(8):1008-9. doi: 10.1007/s12098-011-0371-7. PubMed PMID: WOS:000293143700015.

30. Valamparampil JJ, Chirakkarot S, Letha S, Jayakumar C, Gopinathan KM. Clinical profile of Chikungunya in infants. Indian J Pediatr. 2009;76(2):151-5. doi: 10.1007/s12098-009-0045-x. PubMed PMID: WOS:000264631100003.

31. Vasani R, Kanhere S, Chaudhari K, Phadke V, Mukherjee P, Gupta S, et al. Congenital Chikungunya-A Cause of Neonatal Hyperpigmentation. Pediatr Dermatol. 2016;33(2):209-12. doi: 10.1111/pde.12650. PubMed PMID: WOS:000373067800055.

32. Villamil-Gomez W, Alba-Silvera L, Menco-Ramos A, Gonzalez-Vergara A, Molinares-Palacios T, Barrios-Corrales M, et al. Congenital Chikungunya Virus Infection in Sincelejo, Colombia: A Case Series. J Trop Pediatrics. 2015;61(5):386-92. doi: 10.1093/tropej/fmv051. PubMed PMID: WOS:000365384300010.

33. Rodriguez-Nieves M, Garcia-Garcia I, Garcia-Fragoso L. Perinatally Acquired Chikungunya Infection: The Puerto Rico Experience. Pediatr Infect Dis J. 2016;35(10):1163. doi: 10.1097/INF.0000000000001261. PubMed PMID: 27622689.

34. Gopakumar H, Ramachandran S. Congenital chikungunya. J Clin Neonatol. 2012;1(3):155-6. doi: 10.4103/2249-4847.101704. PubMed PMID: 24027715; PubMed Central PMCID: PMCPMC3762016.

1. Lenglet Y, Barau G, Robillard PY, Randrianaivo H, Michault A, Bouveret A, et al. [Chikungunya infection in pregnancy: Evidence for intrauterine infection in pregnant women and vertical transmission in the parturient. Survey of the Reunion Island outbreak]. J Gynecol Obstet Biol Reprod (Paris). 2006;35(6):578-83. PubMed PMID: 17003745.

2. Robillard PY, Boumahni B, Gerardin P, Michault A, Fourmaintraux A, Schuffenecker I, et al. Vertical maternal fetal transmission of the chikungunya virus - Ten cases among 84 pregnant women. Presse Med. 2006;35(5):785-8. doi: Doi 10.1016/S0755-4982(06)74690-5. PubMed PMID: WOS:000237918800011.

3. Ramful D, Carbonnier M, Pasquet M, Bouhmani B, Ghazouani J, Noormahomed T, et al. Mother-to-child transmission of Chikungunya virus infection. Pediatr Infect Dis J. 2007;26(9):811-5. doi: 10.1097/INF.0b013e3180616d4f. PubMed PMID: WOS:000249455800008.

4. Gerardin P, Barau G, Michault A, Bintner M, Randrianaivo H, Choker G, et al. Multidisciplinary prospective study of mother-to-child chikungunya virus infections on the Island of La Reunion. Plos Med. 2008;5(3):413-23. doi: ARTN 060

10.1371/journal.pmed.0050060. PubMed PMID: WOS:000254928900016.

5. Fritel X, Rollot O, Gerardin P, Gauzere BA, Bideault J, Lagarde L, et al. Chikungunya virus infection during pregnancy, Reunion, France, 2006. Emerg Infect Dis. 2010;16(3):418-25. doi: 10.3201/eid1603.091403. PubMed PMID: 20202416; PubMed Central PMCID: PMCPMC3322036.

6. Ramful D, Samperiz S, Fritel X, Michault A, Jaffar-Bandjee MC, Rollot O, et al. Antibody kinetics in infants exposed to Chikungunya virus infection during pregnancy reveals absence of congenital infection. J Infect Dis. 2014;209(11):1726-30. doi: 10.1093/infdis/jit814. PubMed PMID: 24338351.

7. Gerardin P, Samperiz S, Ramful D, Boumahni B, Bintner M, Alessandri JL, et al. Neurocognitive outcome of children exposed to perinatal mother-to-child Chikungunya virus infection: the CHIMERE cohort study on Reunion Island. PLoS Negl Trop Dis. 2014;8(7):e2996. doi: 10.1371/journal.pntd.0002996. PubMed PMID: 25033077; PubMed Central PMCID: PMCPMC4102444.

8. Sissoko D, Malvy D, Giry C, Delmas G, Paquet C, Gabrie P, et al. Outbreak of Chikungunya fever in Mayotte, Comoros archipelago, 2005-2006. T Roy Soc Trop Med H. 2008;102(8):780-6. doi: 10.1016/j.trstmh.2008.02.018. PubMed PMID: WOS:000258201600008.

9. Watanaveeradej V, Endy TP, Simasathien S, Kerdpanich A, Polprasert N, Aree C, et al. Transplacental chikungunya virus antibody kinetics, Thailand. Emerg Infect Dis. 2006;12(11):1770-2. PubMed PMID: WOS:000241573900025.

10. Senanayake MP SS, Vidanage KK, Gunassena S, Lamabadusurlya SP. Vertical transmission in Chikungunya infection. Cylon Med J. 2009;54(2):47-50.

11. Laoprasopwattana K, Suntharasaj T, Petmanee P, Suddeaugrai O, Geater A. Chikungunya and dengue virus infections during pregnancy: seroprevalence, seroincidence and maternal-fetal transmission, southern Thailand, 2009-2010. Epidemiol Infect. 2016;144(2):381-8. doi: 10.1017/S0950268815001065. PubMed PMID: WOS:000368638100020.

12. Torres JR, Falleiros-Arlant LH, Duenas L, Pleitez-Navarrete J, Salgado DM, Brea-Del Castillo J. Congenital and perinatal complications of chikungunya fever: a Latin American experience. Int J Infect Dis. 2016;51:85-8. doi: 10.1016/j.ijid.2016.09.009. PubMed PMID: WOS:000388326700020.

13. Escobar M, Nieto AJ, Loaiza-Osorio S, Barona JS, Rosso F. Pregnant Women Hospitalized with Chikungunya Virus Infection, Colombia, 2015. Emerg Infect Dis. 2017;23(11):1777-83. doi: 10.3201/eid2311.170480. PubMed PMID: WOS:000413109500002.

14. Touret Y, Randrianaivo H, Michault A, Schuffenecker I, Kauffmann E, Lenglet Y, et al. Early maternal-fetal transmission of the Chikungunya virus. Presse Med. 2006;35(11):1656-8. doi: Doi 10.1016/S0755-4982(06)74874-6. PubMed PMID: WOS:000242164400010.

15. Robin S, Rainful D, Le Seach F, Jaffar-Bandjee MC, Rigou G, Alessandri JL. Neurologic manifestations of pediatric chikungunya infection. J Child Neurol. 2008;23(9):1028-35. doi: 10.1177/0883073808314151. PubMed PMID: WOS:000258841800007.

16. Gerardin P, Couderc T, Randrianaivo H, Fritel X, Lecuit M. CHIKUNGUNYA VIRUS-ASSOCIATED ENCEPHALITIS: A COHORT STUDY ON LA REUNION ISLAND, 2005-2009 Response. Neurology. 2016;86(21):2025-6. PubMed PMID: WOS:000376959900023.

17. Boumahni B, Kaplan C, Clabe A, Randrianaivo H, Lanza F. Maternal-fetal chikungunya infection associated with Bernard-Soulier syndrome. Arch Pediatrie. 2011;18(3):272-5. doi: 10.1016/j.arcped.2010.12.002. PubMed PMID: WOS:000288186400006.

18. Alvarado-Socarras JL, Ocampo-Gonzalez M, Vargas-Soler JA, Rodriguez-Morales AJ, Franco-Paredes C. Congenital and Neonatal Chikungunya in Colombia. J Pediatr Infect Dis. 2016;5(3):E17-E20. doi: 10.1093/jpids/piw021. PubMed PMID: WOS:000386138100001.

19. Bandeira AC, Campos GS, Sardi SI, Rocha VFD, Rocha GCM. Neonatal encephalitis due to Chikungunya vertical transmission: First report in Brazil. IDCases. 2016;5:57-9. doi: 10.1016/j.idcr.2016.07.008. PubMed PMID: WOS:000399150800019.

20. Evans-Gilbert T. Case Report: Chikungunya and Neonatal Immunity: Fatal Vertically Transmitted Chikungunya Infection. Am J Trop Med Hyg. 2017;96(4):913-5. doi: 10.4269/ajtmh.16-0491. PubMed PMID: WOS:000401763000027.

21. Karthiga V, Kommu PPK, Krishnan L. Perinatal chikungunya in twins. J Pediatr Neurosci. 2016;11(3):223-4. doi: 10.4103/1817-1745.193369. PubMed PMID: WOS:000390115700012.

22. Khandelwal K, Aara N, Ghiya BC, Bumb RA, Satoskar AR. Centro-Facial Pigmentation in Asymptomatic Congenital Chikungunya Viral Infection. J Paediatr Child H. 2012;48(6):542-3. doi: 10.1111/j.1440-1754.2012.02484.x. PubMed PMID: WOS:000305186200021.

23. Kumar N, Gupta V, Thomas N. Brownie-nose: Hyperpigmentation in Neonatal Chikungunya. Indian Pediatr. 2014;51(5):419-. PubMed PMID: WOS:000336049800023.

24. Lyra PPR, Campos GS, Bandeira ID, Sardi SI, Costa LFD, Santos FR, et al. Congenital Chikungunya Virus Infection after an Outbreak in Salvador, Bahia, Brazil. Ajp Rep. 2016;6(3):E299-E300. doi: 10.1055/s-0036-1587323. PubMed PMID: WOS:000382531200008.

25. Passi GR, Khan YZ, Chitnis DS. Chikungunya infection in neonates. Indian Pediatr. 2008;45(3):240-2. PubMed PMID: WOS:000254357300016.

26. Boumahni B, Bintner M. [Five-year outcome of mother-to-child transmission of chikungunya virus]. Med Trop (Mars). 2012;72 Spec No:94-6. PubMed PMID: 22693938.

27. Pinzon-Redondo H, Paternina-Caicedo A, Barrios-Redondo K, Zarate-Vergara A, Tirado-Perez I, Fortich R, et al. RISK FACTORS FOR SEVERITY OF CHIKUNGUNYA IN CHILDREN A Prospective Assessment. Pediatr Infect Dis J. 2016;35(6):702-4. doi: 10.1097/Inf.0000000000001135. PubMed PMID: WOS:000379343700024.

28. Shenoy S, Pradeep GCM. Neurodevelopmental Outcome of Neonates with Vertically Transmitted Chikungunya Fever with Encephalopathy. Indian Pediatr. 2012;49(3):238-40. PubMed PMID: WOS:000304110800015.

29. Shrivastava A, Beg MW, Gujrati C, Gopalan N, Rao PVL. Management of a Vertically Transmitted Neonatal Chikungunya Thrombocytopenia. Indian J Pediatr. 2011;78(8):1008-9. doi: 10.1007/s12098-011-0371-7. PubMed PMID: WOS:000293143700015.

30. Valamparampil JJ, Chirakkarot S, Letha S, Jayakumar C, Gopinathan KM. Clinical profile of Chikungunya in infants. Indian J Pediatr. 2009;76(2):151-5. doi: 10.1007/s12098-009-0045-x. PubMed PMID: WOS:000264631100003.

31. Vasani R, Kanhere S, Chaudhari K, Phadke V, Mukherjee P, Gupta S, et al. Congenital Chikungunya-A Cause of Neonatal Hyperpigmentation. Pediatr Dermatol. 2016;33(2):209-12. doi: 10.1111/pde.12650. PubMed PMID: WOS:000373067800055.

32. Villamil-Gomez W, Alba-Silvera L, Menco-Ramos A, Gonzalez-Vergara A, Molinares-Palacios T, Barrios-Corrales M, et al. Congenital Chikungunya Virus Infection in Sincelejo, Colombia: A Case Series. J Trop Pediatrics. 2015;61(5):386-92. doi: 10.1093/tropej/fmv051. PubMed PMID: WOS:000365384300010.

33. Rodriguez-Nieves M, Garcia-Garcia I, Garcia-Fragoso L. Perinatally Acquired Chikungunya Infection: The Puerto Rico Experience. Pediatr Infect Dis J. 2016;35(10):1163. doi: 10.1097/INF.0000000000001261. PubMed PMID: 27622689.

34. Gopakumar H, Ramachandran S. Congenital chikungunya. J Clin Neonatol. 2012;1(3):155-6. doi: 10.4103/2249-4847.101704. PubMed PMID: 24027715; PubMed Central PMCID: PMCPMC3762016.
