# Supplementary material for: Mother-to-child transmission of Chikungunya virus: A systematic review and meta-analysis
Source: PLoS Negl Trop Dis. 2018 Jun 13;12(6):e0006510. doi: 10.1371/journal.pntd.0006510 (PMC6075784; doi:10.1371/journal.pntd.0006510)
Supplement: S5 Table — (DOCX) [file pntd.0006510.s005.docx]

**S5 Table**: Obstetric complications and Maternal Clinical Manifestations from maternal CHIKV infections during gestation (in the analyzed cohort)

|  |  | Obstetric complications in CHIKV-infected pregnant women | Maternal Clinical Manifestations |
| --- | --- | --- | --- |
| 1 | Lenglet 2006 (La Reunion) | - C/S rate was elevated among 33 women infected in the intrapartum period (39.4%; n=13); the most common reason for these C/S was fetal distress and fetal heart rate abnormalities - Amniotic fluid was meconium stained in 25% of these cases | NR in this report |
| 2 | Robillard 2006 (La Reunion) | - 1/10 symptomatic c-p-CHIKV was premature (36 weeks, 1800 grams) - 6/10 born via C/S | - 2/3 of the 84 infected pregnant women were hospitalized |
| 3 | Ramful 2007 (La Reunion) | Among 38 infected neonates:   - The mean GA was 38 weeks (range 35-41 weeks) - Mean Birth Weight was 3000 grams (range 1900-4010) | - 38 mothers of 38 symptomatic c-p-CHIKV - 18 mothers (50%) developed clinical signs on the day of delivery   2 mothers (5%) had an asymptomatic form of infection |
| 4 | Gerardin 2008 (La Reunion) | - 46/61 fetuses with maternal infections around term (22 peripartum and 39 intrapartum) exhibited deep spikes or late decelarations on fetal heart monitoring - These abnormalities were more likely to occur in neonates of CHIKV-infected mothers than in uninfected control-neonates - The C/S rate among these 61 infected mothers was 42.6%; and significantly exceeded the baseline C/S rate of 17.4% - C/S had no influence on MTCT: C/S rate among infected neonates was 48.7%; while C/S rate among uninfected neonates was 52.9%, NS difference - The placental viral load was significantly higher in 7/19 transmitters vs the 13 placenta from non-transmitter mothers (42,000+/- 20,167 copies/mg of tissue vs 10,742+/- 8,182 copies/mg of tissue) - Among the 19 transmitters, one gave birth to dizygotic twins, one neonate remained uninfected while the other became infected | - NR in this report |
| 5 | Fritel 2010 (La Reunion) | - Infected pregnant women had a higher risk of hospitalization than uninfected control women (40% vs 29%) | Among 658 maternal infections:   - 15% (n-99) occurred in the first trimester - 59% (n=387) occurred in the second trimester - 26% (n=172) occurred in the third trimester   Maternal Clinical Manifestations:   - Fever (62%), arthralgias (93%), headache (54%), edema (54%), diarrhea (12%), aphthae (10%), epistaxis or gingivorrhagia (9%), rash (76%), - Overall 21% (n=137) were hospitalized for CHIKV infection for a median of 2 days (range 1-75 ds) - Only 4 infected women had symptoms in the 7 ds before delivery |
| 6 | Ramful 2014 (La Reunion) | NR in this report | NR in this report |
| 7 | Gerardin 2014 (La Reunion) | NR in this report | NR in this report |
| 8 | Watanaveeradej 2006 (Thailand 1998-1999) | NR | NR |
| 9 | Sissoko 2008 (Mauotte) | NR | - Fever (94%), polyarthralgia (95%, headaches (77%) |
| 10 | Senanayake 2009 (Shri Lanka) | NR   - The timing of maternal infections was: - 42% (21/50) in first trimester - 42% (21/50) in second trimester - 14% (7/50) in third trimester | NR |
| 11 | Laoprasopwattana 2016 (Thailand 2009-2010) | Among 88 recent maternal infections during gestation   - Preterm birth (6%; 5/88) - PROM (0%; 0/88) - Abnormal Vaginal Bleeding (1%; 1/88) - GA 38.9 wks (38-41 wks) - Normal vaginal delivery 98% (86/88) - None of the newborns had perinatal asphyxia (0%) - None of the newborns had congenital anomalies (0%) | NR |
| 12 | Torres 2016 (El Salvador) | NR | Among 191 infected pregnant women   - All were infected in the third trimester |
| 13 | Torres 2016 (Santo Domingo) | NR | NR |
| 14 | Torres 2016 (Colombia) | NR | NR |
| 15 | Escobar 2017 (Colombia) | - First trimester: 1 APFD (however 2/3 of the pregnant women were in their third trimester) - Second trimester: 3 preeclampsia, 1 CHIKV sepsis-ICU admission - Third trimester: 8 CHIKV sepsis - **No maternal death** - Third trimester maternal infections:   - PROM (8%, 3/38),   - IUGR (5%, 2/38),   - preeclampsia (16%, 6/38),   - preterm delivery (3%, 1/38),   - postdelivery hemorrhages (8%; 3/38) | - Fever, arthralgias, headache, rash, myalgias, hemorrhagic manifestations among pregnant women in the third trimester; post-delivery hemorrhages (11%), leukopenia, thrombocytopenia, elevated transaminases, hyponatremia, hypokalemia - CHIKV infection also caused severe sepsis syndrome with organ dysfunction and tissue hypoperfusion. - 9/60 ICU admission with severe maternal morbidity: haptic dysfunction (n=7); renal dysfunction (n=2); vascular dysfunction (n=2); severe preeclampsia (n=3); severe postpartum hemorrhage (n=1); sepsis syndrome (n=9); need for Blood transfusion (n=2 - Lactic acid elevation (2.6 ±2.39 mmol/L for 9/60; 15% with ICU admissions); base deficit (-5 ±1.4 mmol/L - Need for fluid resuscitation (15%; 9/9 ICU admissions) - Among 36 women with 1 year post delivery clinical f/up 36% (13/36) had recurrence of their arthralgias ~ 2.5 months after acute CHIKV infections that lasted for ~ 6 months; 5 required evaluation by rheumatologists |

| 31 | 2016 | Rodrigues-Nieves PIDJ 2016 | N=10 c-p CHIK;  Puerto Rico; 8/2014-1/2015 | 10 newborn infants born to mothers with CHIKV like symptoms:   - In 7 newborns; their mothers with symptoms within 5 days PTD (70% of infants with intrapartum infections were symptomatic=higher than previously reported in the literature) - In 3 newborns: their mothers had symptoms >5 days PTD (not specified further timing of maternal infection during gestation) - In the 7/10 newborns whose mothers had symptoms within 5 days PTD: Irritability (50%), eczema (50%), fever (40%), malaise (40%), apnea (40%), tachypnea (40%), poor sucking (30%), cyanosis (30%), peripheral edema (30%), leukopenia (30%), leukocytosis (10%), thrombocytopenia (30%), low albumin (40%), elevated AST (40%), elevated ALT (10%), prolonged PR-interval (10%), prolonged PTT (40%) - In the 3/10 newborns whose mothers had symptoms >5 days PTD (not specified further timing of maternal infection during gestation): No fever, No Irritability BUT with congenital anomalies: Hydrocephalus and Brain Infarct |
| --- | --- | --- | --- | --- |
| 32 | 2016 | Torres International J Infect Dis 2016 | N=169 symptomatic c-p CHIK;  2015 Outbreak  (El Salvador: N=51  Santo Domingo: N= 79; Colombia n=37) | - El Salvador (n=53): Fever (100%); poor feeding (98%); Irritability (91%), rash (85%), hyperalgesis/allodynia (94%), diffuse lower limb edema (87%), hemodynamic instability (53%), dermatosis bullosa/skin scaling (15%), respiratory failure (8%); meningoencephalitis (19%) - Santo Domingo (n=79): Fever (100%); poor feeding (100%); irritability (100%); rash (43%); Hyperalgesia/allodynia (57%); diffuse lower limb edema (42%); dermatosis bullosa (9%); respiratory failure (10%); meningoencephalitis (1%); hyperpigmentation (5%); myocarditis (1%) - Colombia (n=37): Fever (100%); poor feeding (97%); irritability (100%); rash (97%); Hyperalgesia/allodynia (14%); diffuse lower limb edema (27%); hemodynamic instability (3%); dermatosis bullosa (5%); respiratory failure (3%); meningoencephalitis (3%); myocarditis (5%) |
| 33 | 2016 | Vasani Pediatric Dermatology 2016 | N=1 c-p CHIK  India; (NR) | - Full term newborn born to a mother with high grade fever 7 ds PTD, which continued also until 3 days post-delivery (no joint pains or rashes) Mother on day#9 post-delivery developed and blackish discoloration over the nose, lips, streaky pigmentation over the trunk and patchy pigmentation over the extremities; Infant at Birth: mild respiratory distress which resolved by DOL 6 and mild acrocyanosis (without hemodynamic instability; Infant discharged home on DOL#6; DOL#9: Hyperpigmentation (over nose, extension in upper and lower lips; few freckled like macules in alar area bilaterally; flagellate hypergpigmentation over the sides of the trunk; hyperpigmented patches over the dorsa of hands, ankles; groins; penile shaft and scrotum); Infant and Mother CHIKV IgM positive; Hyperpigmentation improved of midface after 6 weeks |

**Abbreviations:** APRF: antepartum fetal deaths; CHIKV: chikungunya virus; c-p-CHIKV: congenital-perinatal CHIKV infections; CP: cerebral palsy; DIC: disseminated intravascular coagulation; DOL: day of life; FT: full term; GA: gestational age; PTD: prior to delivery; c-p-CHIK: congenital-perinatal Chikungunya virus infections; wks: weeks

REFERENCES FOR SUPPLEMENTARY MATERIAL

1. Gerardin P, Barau G, Michault A, Bintner M, Randrianaivo H, Choker G, et al. Multidisciplinary prospective study of mother-to-child chikungunya virus infections on the Island of La Reunion. Plos Med. 2008;5(3):413-23. doi: ARTN 060

10.1371/journal.pmed.0050060. PubMed PMID: WOS:000254928900016.

2. Touret Y, Randrianaivo H, Michault A, Schuffenecker I, Kauffmann E, Lenglet Y, et al. Early maternal-fetal transmission of the Chikungunya virus. Presse Med. 2006;35(11):1656-8. doi: Doi 10.1016/S0755-4982(06)74874-6. PubMed PMID: WOS:000242164400010.

3. Robin S, Rainful D, Le Seach F, Jaffar-Bandjee MC, Rigou G, Alessandri JL. Neurologic manifestations of pediatric chikungunya infection. J Child Neurol. 2008;23(9):1028-35. doi: 10.1177/0883073808314151. PubMed PMID: WOS:000258841800007.

4. Robillard PY, Boumahni B, Gerardin P, Michault A, Fourmaintraux A, Schuffenecker I, et al. Vertical maternal fetal transmission of the chikungunya virus - Ten cases among 84 pregnant women. Presse Med. 2006;35(5):785-8. doi: Doi 10.1016/S0755-4982(06)74690-5. PubMed PMID: WOS:000237918800011.

5. Ramful D, Carbonnier M, Pasquet M, Bouhmani B, Ghazouani J, Noormahomed T, et al. Mother-to-child transmission of Chikungunya virus infection. Pediatr Infect Dis J. 2007;26(9):811-5. doi: 10.1097/INF.0b013e3180616d4f. PubMed PMID: WOS:000249455800008.

6. Lenglet Y, Barau G, Robillard PY, Randrianaivo H, Michault A, Bouveret A, et al. [Chikungunya infection in pregnancy: Evidence for intrauterine infection in pregnant women and vertical transmission in the parturient. Survey of the Reunion Island outbreak]. J Gynecol Obstet Biol Reprod (Paris). 2006;35(6):578-83. PubMed PMID: 17003745.

7. Ramful D, Samperiz S, Fritel X, Michault A, Jaffar-Bandjee MC, Rollot O, et al. Antibody kinetics in infants exposed to Chikungunya virus infection during pregnancy reveals absence of congenital infection. J Infect Dis. 2014;209(11):1726-30. doi: 10.1093/infdis/jit814. PubMed PMID: 24338351.

8. Gerardin P, Couderc T, Randrianaivo H, Fritel X, Lecuit M. CHIKUNGUNYA VIRUS-ASSOCIATED ENCEPHALITIS: A COHORT STUDY ON LA REUNION ISLAND, 2005-2009 Response. Neurology. 2016;86(21):2025-6. PubMed PMID: WOS:000376959900023.

9. Gerardin P, Samperiz S, Ramful D, Boumahni B, Bintner M, Alessandri JL, et al. Neurocognitive outcome of children exposed to perinatal mother-to-child Chikungunya virus infection: the CHIMERE cohort study on Reunion Island. PLoS Negl Trop Dis. 2014;8(7):e2996. doi: 10.1371/journal.pntd.0002996. PubMed PMID: 25033077; PubMed Central PMCID: PMCPMC4102444.

10. Fritel X, Rollot O, Gerardin P, Gauzere BA, Bideault J, Lagarde L, et al. Chikungunya virus infection during pregnancy, Reunion, France, 2006. Emerg Infect Dis. 2010;16(3):418-25. doi: 10.3201/eid1603.091403. PubMed PMID: 20202416; PubMed Central PMCID: PMCPMC3322036.

11. Boumahni B, Kaplan C, Clabe A, Randrianaivo H, Lanza F. Maternal-fetal chikungunya infection associated with Bernard-Soulier syndrome. Arch Pediatrie. 2011;18(3):272-5. doi: 10.1016/j.arcped.2010.12.002. PubMed PMID: WOS:000288186400006.

12. Alvarado-Socarras JL, Ocampo-Gonzalez M, Vargas-Soler JA, Rodriguez-Morales AJ, Franco-Paredes C. Congenital and Neonatal Chikungunya in Colombia. J Pediatr Infect Dis. 2016;5(3):E17-E20. doi: 10.1093/jpids/piw021. PubMed PMID: WOS:000386138100001.

13. Bandeira AC, Campos GS, Sardi SI, Rocha VFD, Rocha GCM. Neonatal encephalitis due to Chikungunya vertical transmission: First report in Brazil. IDCases. 2016;5:57-9. doi: 10.1016/j.idcr.2016.07.008. PubMed PMID: WOS:000399150800019.

14. Evans-Gilbert T. Case Report: Chikungunya and Neonatal Immunity: Fatal Vertically Transmitted Chikungunya Infection. Am J Trop Med Hyg. 2017;96(4):913-5. doi: 10.4269/ajtmh.16-0491. PubMed PMID: WOS:000401763000027.

15. Karthiga V, Kommu PPK, Krishnan L. Perinatal chikungunya in twins. J Pediatr Neurosci. 2016;11(3):223-4. doi: 10.4103/1817-1745.193369. PubMed PMID: WOS:000390115700012.

16. Khandelwal K, Aara N, Ghiya BC, Bumb RA, Satoskar AR. Centro-Facial Pigmentation in Asymptomatic Congenital Chikungunya Viral Infection. J Paediatr Child H. 2012;48(6):542-3. doi: 10.1111/j.1440-1754.2012.02484.x. PubMed PMID: WOS:000305186200021.

17. Kumar N, Gupta V, Thomas N. Brownie-nose: Hyperpigmentation in Neonatal Chikungunya. Indian Pediatr. 2014;51(5):419-. PubMed PMID: WOS:000336049800023.

18. Laoprasopwattana K, Suntharasaj T, Petmanee P, Suddeaugrai O, Geater A. Chikungunya and dengue virus infections during pregnancy: seroprevalence, seroincidence and maternal-fetal transmission, southern Thailand, 2009-2010. Epidemiol Infect. 2016;144(2):381-8. doi: 10.1017/S0950268815001065. PubMed PMID: WOS:000368638100020.

19. Lyra PPR, Campos GS, Bandeira ID, Sardi SI, Costa LFD, Santos FR, et al. Congenital Chikungunya Virus Infection after an Outbreak in Salvador, Bahia, Brazil. Ajp Rep. 2016;6(3):E299-E300. doi: 10.1055/s-0036-1587323. PubMed PMID: WOS:000382531200008.

20. Passi GR, Khan YZ, Chitnis DS. Chikungunya infection in neonates. Indian Pediatr. 2008;45(3):240-2. PubMed PMID: WOS:000254357300016.

21. Boumahni B, Bintner M. [Five-year outcome of mother-to-child transmission of chikungunya virus]. Med Trop (Mars). 2012;72 Spec No:94-6. PubMed PMID: 22693938.

22. Pinzon-Redondo H, Paternina-Caicedo A, Barrios-Redondo K, Zarate-Vergara A, Tirado-Perez I, Fortich R, et al. RISK FACTORS FOR SEVERITY OF CHIKUNGUNYA IN CHILDREN A Prospective Assessment. Pediatr Infect Dis J. 2016;35(6):702-4. doi: 10.1097/Inf.0000000000001135. PubMed PMID: WOS:000379343700024.

23. Senanayake MP SS, Vidanage KK, Gunassena S, Lamabadusurlya SP. Vertical transmission in Chikungunya infection. Cylon Med J. 2009;54(2):47-50.

24. Shenoy S, Pradeep GCM. Neurodevelopmental Outcome of Neonates with Vertically Transmitted Chikungunya Fever with Encephalopathy. Indian Pediatr. 2012;49(3):238-40. PubMed PMID: WOS:000304110800015.

25. Shrivastava A, Beg MW, Gujrati C, Gopalan N, Rao PVL. Management of a Vertically Transmitted Neonatal Chikungunya Thrombocytopenia. Indian J Pediatr. 2011;78(8):1008-9. doi: 10.1007/s12098-011-0371-7. PubMed PMID: WOS:000293143700015.

26. Sissoko D, Malvy D, Giry C, Delmas G, Paquet C, Gabrie P, et al. Outbreak of Chikungunya fever in Mayotte, Comoros archipelago, 2005-2006. T Roy Soc Trop Med H. 2008;102(8):780-6. doi: 10.1016/j.trstmh.2008.02.018. PubMed PMID: WOS:000258201600008.

27. Torres JR, Falleiros-Arlant LH, Duenas L, Pleitez-Navarrete J, Salgado DM, Brea-Del Castillo J. Congenital and perinatal complications of chikungunya fever: a Latin American experience. Int J Infect Dis. 2016;51:85-8. doi: 10.1016/j.ijid.2016.09.009. PubMed PMID: WOS:000388326700020.

28. Valamparampil JJ, Chirakkarot S, Letha S, Jayakumar C, Gopinathan KM. Clinical profile of Chikungunya in infants. Indian J Pediatr. 2009;76(2):151-5. doi: 10.1007/s12098-009-0045-x. PubMed PMID: WOS:000264631100003.

29. Vasani R, Kanhere S, Chaudhari K, Phadke V, Mukherjee P, Gupta S, et al. Congenital Chikungunya-A Cause of Neonatal Hyperpigmentation. Pediatr Dermatol. 2016;33(2):209-12. doi: 10.1111/pde.12650. PubMed PMID: WOS:000373067800055.

30. Villamil-Gomez W, Alba-Silvera L, Menco-Ramos A, Gonzalez-Vergara A, Molinares-Palacios T, Barrios-Corrales M, et al. Congenital Chikungunya Virus Infection in Sincelejo, Colombia: A Case Series. J Trop Pediatrics. 2015;61(5):386-92. doi: 10.1093/tropej/fmv051. PubMed PMID: WOS:000365384300010.

31. Rodriguez-Nieves M, Garcia-Garcia I, Garcia-Fragoso L. Perinatally Acquired Chikungunya Infection: The Puerto Rico Experience. Pediatr Infect Dis J. 2016;35(10):1163. doi: 10.1097/INF.0000000000001261. PubMed PMID: 27622689.

32. Gopakumar H, Ramachandran S. Congenital chikungunya. J Clin Neonatol. 2012;1(3):155-6. doi: 10.4103/2249-4847.101704. PubMed PMID: 24027715; PubMed Central PMCID: PMCPMC3762016.

1. Lenglet Y, Barau G, Robillard PY, Randrianaivo H, Michault A, Bouveret A, et al. [Chikungunya infection in pregnancy: Evidence for intrauterine infection in pregnant women and vertical transmission in the parturient. Survey of the Reunion Island outbreak]. J Gynecol Obstet Biol Reprod (Paris). 2006;35(6):578-83. PubMed PMID: 17003745.

2. Robillard PY, Boumahni B, Gerardin P, Michault A, Fourmaintraux A, Schuffenecker I, et al. Vertical maternal fetal transmission of the chikungunya virus - Ten cases among 84 pregnant women. Presse Med. 2006;35(5):785-8. doi: Doi 10.1016/S0755-4982(06)74690-5. PubMed PMID: WOS:000237918800011.

3. Ramful D, Carbonnier M, Pasquet M, Bouhmani B, Ghazouani J, Noormahomed T, et al. Mother-to-child transmission of Chikungunya virus infection. Pediatr Infect Dis J. 2007;26(9):811-5. doi: 10.1097/INF.0b013e3180616d4f. PubMed PMID: WOS:000249455800008.

4. Gerardin P, Barau G, Michault A, Bintner M, Randrianaivo H, Choker G, et al. Multidisciplinary prospective study of mother-to-child chikungunya virus infections on the Island of La Reunion. Plos Med. 2008;5(3):413-23. doi: ARTN 060

10.1371/journal.pmed.0050060. PubMed PMID: WOS:000254928900016.

5. Fritel X, Rollot O, Gerardin P, Gauzere BA, Bideault J, Lagarde L, et al. Chikungunya virus infection during pregnancy, Reunion, France, 2006. Emerg Infect Dis. 2010;16(3):418-25. doi: 10.3201/eid1603.091403. PubMed PMID: 20202416; PubMed Central PMCID: PMCPMC3322036.

6. Ramful D, Samperiz S, Fritel X, Michault A, Jaffar-Bandjee MC, Rollot O, et al. Antibody kinetics in infants exposed to Chikungunya virus infection during pregnancy reveals absence of congenital infection. J Infect Dis. 2014;209(11):1726-30. doi: 10.1093/infdis/jit814. PubMed PMID: 24338351.

7. Gerardin P, Samperiz S, Ramful D, Boumahni B, Bintner M, Alessandri JL, et al. Neurocognitive outcome of children exposed to perinatal mother-to-child Chikungunya virus infection: the CHIMERE cohort study on Reunion Island. PLoS Negl Trop Dis. 2014;8(7):e2996. doi: 10.1371/journal.pntd.0002996. PubMed PMID: 25033077; PubMed Central PMCID: PMCPMC4102444.

8. Sissoko D, Malvy D, Giry C, Delmas G, Paquet C, Gabrie P, et al. Outbreak of Chikungunya fever in Mayotte, Comoros archipelago, 2005-2006. T Roy Soc Trop Med H. 2008;102(8):780-6. doi: 10.1016/j.trstmh.2008.02.018. PubMed PMID: WOS:000258201600008.

9. Watanaveeradej V, Endy TP, Simasathien S, Kerdpanich A, Polprasert N, Aree C, et al. Transplacental chikungunya virus antibody kinetics, Thailand. Emerg Infect Dis. 2006;12(11):1770-2. PubMed PMID: WOS:000241573900025.

10. Senanayake MP SS, Vidanage KK, Gunassena S, Lamabadusurlya SP. Vertical transmission in Chikungunya infection. Cylon Med J. 2009;54(2):47-50.

11. Laoprasopwattana K, Suntharasaj T, Petmanee P, Suddeaugrai O, Geater A. Chikungunya and dengue virus infections during pregnancy: seroprevalence, seroincidence and maternal-fetal transmission, southern Thailand, 2009-2010. Epidemiol Infect. 2016;144(2):381-8. doi: 10.1017/S0950268815001065. PubMed PMID: WOS:000368638100020.

12. Torres JR, Falleiros-Arlant LH, Duenas L, Pleitez-Navarrete J, Salgado DM, Brea-Del Castillo J. Congenital and perinatal complications of chikungunya fever: a Latin American experience. Int J Infect Dis. 2016;51:85-8. doi: 10.1016/j.ijid.2016.09.009. PubMed PMID: WOS:000388326700020.

13. Escobar M, Nieto AJ, Loaiza-Osorio S, Barona JS, Rosso F. Pregnant Women Hospitalized with Chikungunya Virus Infection, Colombia, 2015. Emerg Infect Dis. 2017;23(11):1777-83. doi: 10.3201/eid2311.170480. PubMed PMID: WOS:000413109500002.

14. Touret Y, Randrianaivo H, Michault A, Schuffenecker I, Kauffmann E, Lenglet Y, et al. Early maternal-fetal transmission of the Chikungunya virus. Presse Med. 2006;35(11):1656-8. doi: Doi 10.1016/S0755-4982(06)74874-6. PubMed PMID: WOS:000242164400010.

15. Robin S, Rainful D, Le Seach F, Jaffar-Bandjee MC, Rigou G, Alessandri JL. Neurologic manifestations of pediatric chikungunya infection. J Child Neurol. 2008;23(9):1028-35. doi: 10.1177/0883073808314151. PubMed PMID: WOS:000258841800007.

16. Gerardin P, Couderc T, Randrianaivo H, Fritel X, Lecuit M. CHIKUNGUNYA VIRUS-ASSOCIATED ENCEPHALITIS: A COHORT STUDY ON LA REUNION ISLAND, 2005-2009 Response. Neurology. 2016;86(21):2025-6. PubMed PMID: WOS:000376959900023.

17. Boumahni B, Kaplan C, Clabe A, Randrianaivo H, Lanza F. Maternal-fetal chikungunya infection associated with Bernard-Soulier syndrome. Arch Pediatrie. 2011;18(3):272-5. doi: 10.1016/j.arcped.2010.12.002. PubMed PMID: WOS:000288186400006.

18. Alvarado-Socarras JL, Ocampo-Gonzalez M, Vargas-Soler JA, Rodriguez-Morales AJ, Franco-Paredes C. Congenital and Neonatal Chikungunya in Colombia. J Pediatr Infect Dis. 2016;5(3):E17-E20. doi: 10.1093/jpids/piw021. PubMed PMID: WOS:000386138100001.

19. Bandeira AC, Campos GS, Sardi SI, Rocha VFD, Rocha GCM. Neonatal encephalitis due to Chikungunya vertical transmission: First report in Brazil. IDCases. 2016;5:57-9. doi: 10.1016/j.idcr.2016.07.008. PubMed PMID: WOS:000399150800019.

20. Evans-Gilbert T. Case Report: Chikungunya and Neonatal Immunity: Fatal Vertically Transmitted Chikungunya Infection. Am J Trop Med Hyg. 2017;96(4):913-5. doi: 10.4269/ajtmh.16-0491. PubMed PMID: WOS:000401763000027.

21. Karthiga V, Kommu PPK, Krishnan L. Perinatal chikungunya in twins. J Pediatr Neurosci. 2016;11(3):223-4. doi: 10.4103/1817-1745.193369. PubMed PMID: WOS:000390115700012.

22. Khandelwal K, Aara N, Ghiya BC, Bumb RA, Satoskar AR. Centro-Facial Pigmentation in Asymptomatic Congenital Chikungunya Viral Infection. J Paediatr Child H. 2012;48(6):542-3. doi: 10.1111/j.1440-1754.2012.02484.x. PubMed PMID: WOS:000305186200021.

23. Kumar N, Gupta V, Thomas N. Brownie-nose: Hyperpigmentation in Neonatal Chikungunya. Indian Pediatr. 2014;51(5):419-. PubMed PMID: WOS:000336049800023.

24. Lyra PPR, Campos GS, Bandeira ID, Sardi SI, Costa LFD, Santos FR, et al. Congenital Chikungunya Virus Infection after an Outbreak in Salvador, Bahia, Brazil. Ajp Rep. 2016;6(3):E299-E300. doi: 10.1055/s-0036-1587323. PubMed PMID: WOS:000382531200008.

25. Passi GR, Khan YZ, Chitnis DS. Chikungunya infection in neonates. Indian Pediatr. 2008;45(3):240-2. PubMed PMID: WOS:000254357300016.

26. Boumahni B, Bintner M. [Five-year outcome of mother-to-child transmission of chikungunya virus]. Med Trop (Mars). 2012;72 Spec No:94-6. PubMed PMID: 22693938.

27. Pinzon-Redondo H, Paternina-Caicedo A, Barrios-Redondo K, Zarate-Vergara A, Tirado-Perez I, Fortich R, et al. RISK FACTORS FOR SEVERITY OF CHIKUNGUNYA IN CHILDREN A Prospective Assessment. Pediatr Infect Dis J. 2016;35(6):702-4. doi: 10.1097/Inf.0000000000001135. PubMed PMID: WOS:000379343700024.

28. Shenoy S, Pradeep GCM. Neurodevelopmental Outcome of Neonates with Vertically Transmitted Chikungunya Fever with Encephalopathy. Indian Pediatr. 2012;49(3):238-40. PubMed PMID: WOS:000304110800015.

29. Shrivastava A, Beg MW, Gujrati C, Gopalan N, Rao PVL. Management of a Vertically Transmitted Neonatal Chikungunya Thrombocytopenia. Indian J Pediatr. 2011;78(8):1008-9. doi: 10.1007/s12098-011-0371-7. PubMed PMID: WOS:000293143700015.

30. Valamparampil JJ, Chirakkarot S, Letha S, Jayakumar C, Gopinathan KM. Clinical profile of Chikungunya in infants. Indian J Pediatr. 2009;76(2):151-5. doi: 10.1007/s12098-009-0045-x. PubMed PMID: WOS:000264631100003.

31. Vasani R, Kanhere S, Chaudhari K, Phadke V, Mukherjee P, Gupta S, et al. Congenital Chikungunya-A Cause of Neonatal Hyperpigmentation. Pediatr Dermatol. 2016;33(2):209-12. doi: 10.1111/pde.12650. PubMed PMID: WOS:000373067800055.

32. Villamil-Gomez W, Alba-Silvera L, Menco-Ramos A, Gonzalez-Vergara A, Molinares-Palacios T, Barrios-Corrales M, et al. Congenital Chikungunya Virus Infection in Sincelejo, Colombia: A Case Series. J Trop Pediatrics. 2015;61(5):386-92. doi: 10.1093/tropej/fmv051. PubMed PMID: WOS:000365384300010.

33. Rodriguez-Nieves M, Garcia-Garcia I, Garcia-Fragoso L. Perinatally Acquired Chikungunya Infection: The Puerto Rico Experience. Pediatr Infect Dis J. 2016;35(10):1163. doi: 10.1097/INF.0000000000001261. PubMed PMID: 27622689.

34. Gopakumar H, Ramachandran S. Congenital chikungunya. J Clin Neonatol. 2012;1(3):155-6. doi: 10.4103/2249-4847.101704. PubMed PMID: 24027715; PubMed Central PMCID: PMCPMC3762016.
