# Supplementary material for: Mother-to-child transmission of Chikungunya virus: A systematic review and meta-analysis
Source: PLoS Negl Trop Dis. 2018 Jun 13;12(6):e0006510. doi: 10.1371/journal.pntd.0006510 (PMC6075784; doi:10.1371/journal.pntd.0006510)
Supplement: S6 Table — (DOCX) [file pntd.0006510.s006.docx]

**S6 Table:** Clinical Manifestations in neonatal CHIKV-infections from maternal infections during gestation (listed in alphabetical order)

| **General/Systemic** | **Cardiologic** | **Neurologic** |
| --- | --- | --- |
| asymptomatic | anasarca edema (shock) | ataxia |
| Anorexia | coronary artery dilation (ECHO) | blindness |
| diffuse pain/hyperalgesia/allodynia syndrome | heart failure | brain hemorrhages |
| Fever | hemodynamic instability | CSF hypoglycorachea |
| hemorrhagic fever/hemorrhagic syndrome | hypotension | CSF protein elevation (>100 mg/dl) |
| irritability (constant cry/paradoxical cry) | left ventricular dysfunction | EEG abnormalities |
| Malaise | left ventricular hypertrophy | encephalitis/ meningoencephalitis |
| poor feeding/poor sucking | myocardial dysfunction | encephalopathy (also with normal CSF chemistry and cytology) |
| sepsis like picture/shock like picture | myocarditis | febrile seizures |
| **Fetal/Neonatal** | pericarditis | hydrocephalus and brain infarct |
| antepartum fetal death <22 wks (due to CHIKV by AF-PCR) | poor perfusion | hypotonia |
| antepartum fetal death >22 wks (due to CHIKV by AF-PCR) | prolonged PR-interval | meningeal syndrome |
| congenital malformations* | pulmonary artery hypertension | optic atrophy |
| fetal distress (need for emergency C/S) | septal dyskinesia (ECHO) | seizures |
| neonatal death | shock refractory (suggestive of myocarditis) | status epilepticus |
| placental histologic abnormalities rare** | shock/hypovolemia | strabismus |
| prematurity (during acute maternal febrile CHIKV infection) | **Respiratory** | Visual Evoked Potentials (VEPs) abnormalities |
| **Hematologic/Metabolic** | persistent apnea (requiring mechanical ventilation) | **Neurologic-long term outcome** |
| ALT elevation | respiratory distress mild | normal |
| Anemia | respiratory failure | global neurodevelopmental delays |
| AST elevation | tachypnea | behavioral problems |
| coagulopathy | apnea, cyanosis, hypoxia | cerebral palsy |
| coagulopathy (requiring FFP transfusion) | **Rheumatologic** | conceptual thinking inadequate |
| CRP elevation | distal joint edema | hypertonia, spastic diplegia |
| hyperbilirubinemia/direct hyperbilirubinemia | extremity edema | language skills inadequate |
| hypoalbuminemia | flexion deformity of finger (due to tenosynovitis) | microcephaly |
| hypocalcemia | **Cutaneous** | neurodevelopmental disabilities |
| hyponatremia | acrocyanosis (also without hemodynamic instability) | non-meaningful memory inadequate |
| hypoproteinemia | bullous dermatitis | nonverbal and numeric reasoning inadequate |
| hyporpothrombinemia | dermatosis bullosa/skin scaling | numeric reasoning poor |
| leukocytosis | distal limb ischemia | visual-motor skills poor |
| leukopenia | dyschromia cutaneous | **MRI findings in infected infants (from maternal infections during gestation)** |
| lymphopenia | hyperpigmented patches over the dorsa of hands/ankles/Groins/ penile shaft/scrotum (with improvement after 6 weeks) | brain MRI at the acute phase: scattered hyperintense signals on DWI (Diffusion Weighted Imaging) in the supratentorial white matter; involving the corpus callosum; frontal, parietal and temporal lobes evocative of cytotoxic edema. |
| PTT prolongation | hyperpigmentation flagellate over the sides of the trunk | brain MRI at the subacute phase: changes to very low intensity signals in DWI evocative of vasogenic edema |
| renal failure | hyperpigmentation midfacial (with persistence up to 3 mo of age) | Brain MRI subsequently: regression towards cavitation and subcortical atrophy |
| rhabdomyolysis (elevated CPK >1000) | hyperpigmentation over nose, extension in upper and lower lips; freckled like macules in alar area bilaterally | brain MRI: symmetrical hyperintensity in T2 images and in Diffusion Coefficient Imaging: restricted diffusion |
| thrombocytopenia | hyperpigmentation over nose, face and groin | brain MRI with Severe restriction of White Matter Areas predominantly in the frontal lobes |
| Uremia | lymphedema | brain MRI findings at 2 years of age for 8 infants with c-p CHIK encephalopathy: diffuse white matter changes (n=5); demyelination (n=5); cavitations (n=2); gliosis (n=1); spectroscopy with white mater hypometabolism or axonal loss (n=3) |
| **Other** | petechiae | brain MRI at 4 months of infant with encephalopathy, sepsis and DIC (at DOL#4): scattered areas of cytotoxic edema with decreased diffusion signs in ADC (Apparent Diffusion Coefficient); scattered demyelination, thinning of corpus callosum, passive dilation of supratentorial subarachnoid space |
| abdominal distension | roseola-like rash | brain MRI: widespread cytotoxic edema in sub-tentorial white matter; Brain spectroscopy: elevation of lactate |
| absence of organomegaly | rubella-like rash | brain MRI with hyperintensity signals on Diffusion weighted Images (DWI) in the subcortical white matter of frontal, parietal, temporal and corpus callosum |
| adenopathy | oral mucositis (erythematous had palate) | brain MRI: hemorrhagic leukoencephalopathy |
| necrotizing enterocolitis | oral mucositis-oral mucosal bleeding | brain MRI with areas of bleeding in basal ganglia and subcortical areas |
| coffee ground gastric aspirate |  | brain MRI normal (f/up of 10 infants with non-severe c-p CHIK, normal clinical status) |
| Diarrhea |  |  |
| Vomiting |  |  |
| **Footnote**:*(3% [19/658] in CHIKV infected vs 2.2% [15/655] in non-infected pregnant women (However only 15% [99 /658] of CHIK infected pregnant women were infected in the 1st trimester; it would have required 1,340 women in each group to a doubling of the risk [4% vs 2%] with an 80% power); ** (e.g only in 1/624 examined placentas from CHIK infected pregnant women); **Abbreviations**: APFD: antepartum fetal deaths; AF: amniotic fluid; DWI: diffusion weighted images; mo: months; PCR: polymerase chain reaction; wks: weeks; | | |
