# Supplementary material for: Mother-to-child transmission of Chikungunya virus: A systematic review and meta-analysis
Source: PLoS Negl Trop Dis. 2018 Jun 13;12(6):e0006510. doi: 10.1371/journal.pntd.0006510 (PMC6075784; doi:10.1371/journal.pntd.0006510)
Supplement: S7 Table — (DOCX) [file pntd.0006510.s007.docx]

**S7 Table:** Clinical Manifestations of Congenital/Neonatal CHIKV Infections, from Maternal Infections During Gestation (per study) [1-32]

|  |  | Author, year | N of c-p CHIKV cases, country, year | Clinical manifestations |
| --- | --- | --- | --- | --- |
| 1 | 2006 | Touret Press Medical 2006 (French) | N=3 APFD from c-CHIK (at 12 wks 4/7 ds; 15 wks; 15 wks 5/7 ds);La Reunion; -3/2006 | - 3 confirmed APFD with Documented Early Maternal Infections (Early in gestation) - Three spontaneous abortions at 12w4d, 15w0d and 15w5d. RT-PCR showed viral genome in amniotic fluid (n=3); brain (n=2) and placentas n=2). |
| 2 | 2006 | Robillard Press Medical 2006 (French) | N=10 c-p CHIK (from peripartum maternal infections);  La Reunion;  6/2005-1/2006 | - 10 infants with c-p CHIK from maternal infections at the time of delivery (Viremic mothers, RT-PCR positive and all infants either RT-PCR positive (n=7) or IgM positive(n=7): - Symptoms onset between DOL3-7; extremity edema (n=9), painful syndrome (n=10); rash (n=10), fever, seizures (n=4); DIC(n=3); thrombocytopenia (n=10); severe thrombocytopenia (n=3); One infant with severe thrombocytopenia had GI bleeding and Cerebral bleeding severe; EEG abnormalities (n=5); need for intubation for prolonged apneas (n=6);>No neonatal deaths - Another 74 infants born to mothers with CHIK infection distant to delivery: Asymptomatic (NR for CHIK infection status for these infants) |
| 3 | 2006 | Lenglet J Gynecol Obstet Biol Reprod 2006 | N= 16 c-p CHIK neonates, 3 spontaneous abortions;  La Reunion; 2005-2006 | - Likely overlap with cases reported in subsequent publications from CHIMERE cohort - Rate of prematurity among c-p CHIK infected infants=10% vs 14% in the general population - Among 9 APFD less than 22 weeks: 3 were confirmed to be from CHIKV (in Gerardin 2008 report: 7 APFD less than 22 weeks; 3 confirmed from CHIKV) - 16 infected infants/33 mothers viremic at the time of delivery (in Gerardin 2008 report: 19 infected infants/61 mothers viremic near term [n=39 viremic -2ds to +7 ds and n=22 viremic -7ds to -3ds) - None of the 116 infected mothers who delivered outside the viremic period delivered any infected infant (all infants were IgM negative at birth; NR for additional subsequent serologic testing of infants) |
| 4 | 2007 | Ramful PIDJ 2007 | N=38 c-p CHIK;  La Reunion; 3/2005-4/2006 | - 38 infants (24 were RT-PCR positive and 26 were CHIKV IgM positive) (Mothers with signs of CHIKV infection in the perinatal period -4 Ds to +1 d) - Pain score elevated (n=38; 100%); fever (n=30; 79%); rash (n=31; 82%); edema (n=22; 58%); diarrhea (n=12; 32%); seizures (n=6; 16%); hemorrhagic syndrome (n=6; 16%); hemodynamic disorder (n=10; 26%); neonatal death (n=1; 3%) - Thrombocytopenia <150,000/ml (n=29; 76%); Severe thrombocytopenia <50,000/ml (n=12; 32%); Lymphopenia (n=18; 47%); hypocalcemia (n=5; 19%); hypoprothrombinemia (n=17; 65%); transaminitis (n=23; 77%) - Cardiologic findings: Among 16 infants undergone cardiovascular investigation: 6 had abnormalities: - infant 1: Clinical=severe pain; ECHO=right/left coronary artery dilation; Left ventricular hypertrophy; Left ventricular dysfunction; AST=250; CK 1385; CKMB=148; TIc=0.13 - infant 2: Clinical: bradycardia, desaturation, severe pain; ECHO=pericarditis, left ventricular hypertrophy; right coronary artery dilation; AST=224; CK=460; CKMB=49; TIc=0.1 - infant 3: Clinical: Heart failure, collapse; ECHO=pericarditis, left ventricular hypertrophy; left ventricular dysfunction, pulmonary artery hypertension; right/left coronary dilation; AST=172; CK=236; CKMB=62; TIc=0.15//infant 4: Clinical: Severe pain, tachycardia; ECHO= right coronary dilation; AST=123; CK=306; CKMB=66; Tic=0.1//infant 5: Clinical=Severe pain, malaise; ECHO=left ventricular hypertrophy; right/left coronary dilation; AST=136; CK=1417; CKMB=74; TIc=0.11//infant 6: Clinical=Heart failure/collapse; ECHO=left ventricular hypertrophy with septal dyskinesia, right/left coronary dilation; AST=161; CK=1284; CKMB=125; TIc=1.64 |
| 5 | 2008 | Geradin 2008 | N= 19 c-p CHIK cases; La Reunion;  2005-2006 | - 19 infected newborns/61 peripartum infected mothers (viremic)   - 10/19 had severe neonatal disease - Among the 19 infected infants: median onset of disease DOL#4 (range 3-7); 0/16 infected neonates had positive CHIKV RT-PCR on DOL#1: fever (n=19; 100%), poor feeding (n=19; 100%), pain & need for analgesia (n=19; 100%); rheumatic signs (distal joint edema n=15/18; 79%); cutaneous signs (petechia n=9/19; 47%, polymorphous rubella-like rash n=10/19; 53%, roseola like rash n=7/19; 37%); Thrombocytopenia (n=17/19; 89%); severe thrombocytopenia (n=9/19; 47%); mild coagulopathy (32%); lymphopenia (n=13/19; 69%); AST elevation (n=10/19; 53%); moderate/severe hypocalcemia (n=9/19; 47%) - Among the 10/19 infants with severe c-p CHIKV: encephalopathy (n=9); hemorrhagic fever (n=1); CSF CHIKV RT-PCR positive (n=5/9 encephalopathic cases; with normal CSF chemistry and CSF cytology; except for 3 cases who had 3,500 RBC/mm3 in CSF; one of which with DIC); need for mechanical ventilation (n=7; for a median7 ds) ; shock/hypovolemia (n=6) hypokinesis in ECHO (n=4); need for vasoactive medications (n=4; for 2 ds); DIC(n=4; with brain parenchymal hemorrhages) - No neonatal mortality even in infants with severe shock or massive hemorrhage - MRI abnormalities in infants with encephalopathy:   - At the acute phase: scattered hyperintense signals on DWI in the supratentorial white matter; involving the corpus callosum; frontal, parietal and temporal lobes evocative of cytotoxic edema.   - At the subacute phase: changes to very low intensity signals in DWI evocative of vasogenic edema   - Subsequently: regression towards cavitation and subcortical atrophy - 3 confirmed APFD from CHIKV / 678 infected pregnant women with infections <22 wks - APFD (antepartum fetal deaths): 9 from antepartum maternal infections >22 weeks (0/9 confirmed to be due to CHIKV); 7 from Antepartum maternal infections<22 weeks (3/7 confirmed to be due to CHIKV) - Long term f/up of 9 infants with severe c-p CHIKV infection and encephalopathy: (follow up to 16-24 months): 4/9 developed persistent disabilities: one CP, ataxia and blindness; 3 behavioral and ocular problems; 4: multiple seizures - Regular f/up of 10 infants with non-severe c-p CHIK: Clinical status and brain MRI was considered normal |
| 6 | 2008 | Robin J Child Neurol 2008 | N=4 c-p CHIKV  (with Neurologic manifestations-Encephalitis);La Reunion; 2005-2006 | - 4/30 children with neurologic manifestations diagnosed <2 weeks of age and could possibly be from c-p CHIKV: 4 of them had diagnosis of Encephalitis: case 1 (10 ds):fever, seizure, somnolence, diffuse pain, rash, Dx: encephalitis; Outcome at D/C: cutaneous dyschromia; case 2 (8 ds):fever, somnolense, hypotonia, seizures, anorexia, diffuse pain, rash, Dx: encephalitis; Outcome at D/C: cutaneous dyschromia; Case 3 (6 ds): fever; seizures; faintness, anorexia, diffuse pain; Dx: Encephalitis; and neurodevelopmental delay at discharge, Brain MRI: symmetrical hyperintensity in T2 images and in Diffusion Coefficient Imaging: restricted diffusion; Cutaneouse dyschromia; case 4 (3 ds): fever, status epilepticus; anorexia, vomiting, rash, Dx: encephalitis; Outcome at D/C: Strabismus, hypotonia - (Overall described 30 infants and children with neurologic manifestations of CHIKV: encephalitis (n=12); febrile seizures (n=10); meningeal syndrome (n=4); acute encephalopathy (n=4); CSF chemistry and cytology were unremarkable except for 1 cases of acute disseminated encephalomyelitis; Abnormal brain MRI (n=5/14); EEG were non specific; Death (n=2) |
| 7 | 2010 | Fritel Emerg Infect Dis 2010 | N=4 c-p CHIK;  La Reunion; 4/2006-5/2206 | - No statistically significant difference in stillbirths and risk for congenital malformations between CHIKV infected and non-infected pregnant women: - Stillbirths >22 weeks: 5/658 in CHIKV infected vs 8/655 in non infected pregnant women; - Miscarriages <22 weeks: 5 in CHIKV infected vs 8 in non-infected pregnant women (although >62% of infected women had fever and contrary to the hypothesis that maternal fever plays a direct role in in utero deaths); - Congenital malformations: 3% [19/658] in CHIKV infected vs 2.2% [15/655] in non-infected pregnant women (However only 15% [99 /658] of CHIK infected pregnant women were infected in the 1^st^ trimester; it would have required 1,340 women in each group to a doubling of the risk [4% vs 2%] with an 80% power); >Rarity of placental Histologic abnormalities (only 1/624 examined placentas from CHIK infected pregnant women) - Among 4 children with maternal CHIKV infections during peripartum period (≤7 ds PTD): 1 was symptomatic on DOL#3 and CHIKV RT-PCR and IgM positive; other 3 were asymptomatic and uninfected (negative CHIK IgM) |
| 8 | 2011 | Boumahni Arch Pediatr 2011 | N=1 c-p CHIK;  La Reunion; (NR) | - Case of materno-fetal CHIKV infection associated with Bernard Suolier syndrome: Full term newborn: DOL#3 thrombocytopenia unresponsive to IVIG; DOL#5: fever (39), “painful syndrome”, seizures, apnea, required mechanical ventilation for 5 days; CHIKV RT-PCR and IgM positive ;Brain MRI: widespread cytotoxic edema in sub-tentorial white matter; Brain spectroscopy: elevation of lactate; Mother of infant also with thrombocytopenia in the 3d trimester of pregnancy, CHIKV RT-PCR and IgM positive. - Infant’s thrombocytopenia: absence of PLT glycoprotein Ib(GPIb) and IX confirming the diagnosis of Bernard Soulier syndrome. |
| 9 | 2012 | Boumahni Med Trop 2012 | N=18 c-p CHIK;  La Reunion; 2005-2006 | - 5 year outcomes of 18 infants with c-p CHIK; 3/18 had cerebral palsy; 2 had blindness, 1 myopia; AERs were normal and no cases with deafness were observed; 9 had behavioral problems and problems with attention and benefited from orthophonic management for language delays - Example of Brain MRI at 5 yrs: Flair T2 with increased signal in the white matter periventricularly |
| 10 | 2014 | Ramful JID 2014 (Serologic/IgG Kinetics study of infants exposed to maternal CHIKV infection) | N=1 c-p CHIK;  La Reunion; 2006-2008 | - Only 1/653 of live born infants exposed to maternal CHIKV infection during pregnancy developed clinical CHIKV infection on DOL#6 with CHIKV RT-PCR positive on serum and CSF (initial CHIKV IgG and IgM were negative; of DOL#3 wks: IgM positive IgG negative; DOL 34 weeks: IgG positive ); Mother had CHIKV infection clinical symptoms 1 day PTD - 652/653 infants remained Asymptomatic (590 who had serologic follow up: none had IgM at DOL # 3; among 580 asymptomatic IgG seropositive infants >10% were IgG positive>1 year of age; - Of those 368 had complete follow up and all of them seroconverted |
| 11 | 2014 | Gerardin PloS Negl trop Dis 2014 | N=33 c-p CHIKV;  La Reunion; 6/2005-4/2006 | - Assessment of neurocognitive function in 33 infants with c-p CHIK at 2 years of age (Another 4 symptomatic infected infants could not have this long term f/up: 1 severely encephalopathic, due to parental decline; 2 with severe neonatal prostration and 1 with CP) - N=12 (12/33) had neonatal CHIKV encephalopathy and subsequently developed Microcephaly (n=5 of those) and Cerebral Palsy (n=4 of those); Brain MRI of those children showed Severe restriction of White Matter Areas predominantly in the frontal lobes of these children. - N=17 (51%) had global Developmental Delay (n=4 severe and n=13 moderate): Of those: coordination/language delay (57%); sociability delay (36%); movement/posture abnormalities (27%); - Brain MRI findings at 2 years of age for 8 infants with c-p CHIK encephalopathy: Diffuse White Matter changes (n=5); Demyelination (n=5); Cavitations (n=2); Gliosis (n=1); Spectroscopy with white mater hypometabolism or axonal loss (n=3) - Brain MRI at 4 mo of age of infant with encephalopathy, sepsis and DIC (at DOL#4): scattered areas of cytotoxic edema with decreased diffusion signs in ADC (Apparent Diffusion Coefficient); scattered demyelination, thinning of corpus callosum, passive dilation of supratentorial subarachnoid space - p-CHIKV was associated with Global Neurodevelopmental Delay, even after adjustment for maternal social status; SGA and head circumference, Gestational age |
| 12 | 2016 | Gerardin Neurology 2016 | N=3 c-p CHIK;  La Reunion; 9/2005-6/2006 | - Case 1: Severe Cerebral Palsy and Hemorrhagic fever DOL#4; Subacute late stage Brain MRI: decrease in cerebral/cerebellar hemorrhages; replacement of brain edema features by subsequent demyelination (ADEM) - Case 2-3 (presumed vertically infected): Normal Neurologic outcome - (the remaining 54 cases with CHIKV associated CNS disease among which 23 with CHIKV encephalitis: were adults and older children) |
| 13 | 2006 | Watanaveeradej Emerg Infect Dis 2006 | N=0 c-p CHIK; Thailand; 1998-1999 | - Not applicable (Among 79 CHIK infected mothers less than half (n=28) agreed to have their infants followed up to 18 months of age; no infant was confirmed to be infected [24/28 infants who were cord blood IgM (+) lost the Abs by 9 months of age |
| 14 | 2007 | Passi Indian Pediatrics 2007 | N=2 c-p CHIK;  India; (NR) | - Full term infant born via C/S due to a mother with pregnancy induced hypertension ; Mother with fever and joint pains1 day PTD; Infant on DOL#3: decreased PO intake; fever (39 C); rash (generalized maculopapular); apnea and cyanosis; irritability and constant cry; (without organomegaly); Infant discharge home on DOL#10; CHIKV RT-PCR positive. - Newborn born via vaginal delivery to a mother with fever and joint pains 3-4 days PTD; Infant on DOL#5: fever, generalized hyperpigmentation (especially over face and nose; also over abdomen); respiratory distress; mucositis (erythematous hard palate); (without organomegaly); thrombocytopenia (PLTs=100,000/ml); (WBC=11,000/ml); Infant discharged home on DOL#7; hyperpigmentation slowly decreasing by 4 months of age |
| 15 | 2008 | Sissoko  Transactions of the Royal Society of Tropical Medicine and Hygiene 2008 | N=9 c-p CHIK;  Mayotte (Indian Ocean); 3/2005-4/2006 | - Neonate infected with CHIK virus after birth (signs first seen at DOL 13) with septicemia caused by K. pneumoniae. - 9 cases of c-p CHIK (6 with severe c-p CHIK): meningoencephalitis (n=3); severe hypotonia (n=-1); conventional form (n=3); severe pain (n=2) - Complications of the severe form: meningoencephalitis (n=2); renal failure (n=2); severe sepsis (n=1); fulminant hepatitis from paracetamol overdose (n=1) - No neonatal mortality of c-p CHIK cases |
| 16 | 2009 | Senanayake Cylon Med J 2009 | N=7 c-p CHIK; Shri Lanka 4/2007-10/2007 | - 7 cases of c-p CHIKV infection with IgM (+) and 3 APFD; however these 3 APFD were not virologically studied (2 abortions with one of these moms having symptoms at 17 wks GA and 1 APFD at 33 wks after a first trimester fetal infection [fetus weighted 1.6 kgs; had multiple congenital abnormalities of the heart and limbs) - There was 1 preterm delivery during the acute febrile phase of maternal CHIKV infection. - 4 women (8%) were clinically viremic at the time of delivery and their infants had serious illness needing hospitalization; however in only 1 neonatal IgM was (+); in 1 could not be done and in 2 was (-): a) case 1: preterm delivery at 30 wks; severe RDS and hyperpigmentation; newborn IgM (-); b) cases 2) term delivery; seizures, microcephaly, developmental delay; newborn serum sample hemolyzed; c) case 3: term delivery; splenomegaly and weight loss; negative neonatal serology; and d) case 4: term delivery; with myocarditis, heart failure and generalized pigmentation; IgM (+). All others had a period of 4 weeks between the febrile phase and delivery: the 36 infants with maternal infections distant to delivery were healthy - There were 11 cases with neonatal hyperpigmentation; however only 3/11 were tested IgM (+(; 6/11 were IgM (-) and 2/11 were not tested |
| 17 | 2009 | Valamparampil Indian J Pediatrics 2009 | N= 4 neonates <1 month of age  India 2007 | - Not applicable; there were 4 CHIKV infected infants <1 months of age, among the 56 infants 0-12 months of age; but their clinical manifestations were not reported separately stools (41%), lethargy and poor feeding (21%), irritability (27%), |
| 18 | 2010 | Shenoy Indian Pediat 2010 | N=2 c-p CHIK;  India; (NR) | - Two newborns with c-p CHIKV encephalopathy who developed persistent disabilities, including cerebral palsy; visual impairment; seizure disorder and behavioral problems - Newborn infant born via C/S to a mother with Fever and Joint pain few days PTD and CHIKV RT-PCR positive; DOL#5: lethargy, seizures, apnea; normal brain CT scan, normal CSF; hypoproteinemia, lymphedema; DOL#7: hyperpigmentation (over nose, face and groin; lasted X2 months); Infant discharged home after 1 week; Long term sequelae were: hypertonia, spastic diplegia; seizures starting at 11 months of age (with normal EEG, CT scan); borderline IQ (IQ=62); Developmental Skills: language: inadequate; non-meaningful memory=inadequate; conceptual thinking: inadequate; nonverbal and numeric reasoning: inadequate; also Hyperactivity - Newborn infant born via C/S to a mother with fever and joint pain just PTD; DOL#3: seizures, apnea and lethargy; Encephalopathy,DOL#4: required mechanical ventilation due to convulsive apneas; hypoproteinemia, lymphedema; Infant was discharged home ~ after 1 week of life; DOL#10: hyperpigmentation (perioral, limb and abdomen); Follow-up: hypotonia; 6 months of age: poor visual contact: primary optic atrophy; VEP: abnormal; 3 years of age: Hypotonic cerebral palsy, mental retardation (IQ=58); Developmental skills: poor social intelligence; poor visual-motor skills; poor numerical reasoning; poor language skills; inadequate conceptual thinking; inadequate verbal reasoning; inadequate meaningful memory. |
| 19 | 2011 | Shrivastava Indian J Pediatr 2011 | N=1 c-p CHIK;  India; (NR) | - Newborn 35 week old infant born via C/S to a mother with fever and joint pain 3 days PTD; Mother CHIKV RT-PCR positive; Infant at birth: mild respiratory distress (retractions); DOL#3: apnea, acrocyanosis; rash (generalized maculopapular); (without organomegaly, normal cardiac exam; Hgb=17.5 g/dl; WBC=8,800/mm3); thrombocytopenia (PLTs=135,000/mm3); DOL#10: Worsening thrombocytopenia (PLTs=17,000/mm3); transaminitis (AST=149 IU/L); (Brain MRI normal); Infant: CHIKV RT-PCR positive; CHIKV IgM: negative; (rash disappeared after 7 ds, no recurrence of apnea); Infant discharged home at 2 weeks of age. |
| 20 | 2012 | Khandewal J Paediatrics and Child Health 2012 | N=1 c-p CHIK;  India; (NR) | - Centro-Facial hyperpigmentation at DOL#10 in an otherwise clinically well afebrile infant; CHIKV IgM positive infant; born at 37 weeks to a mother with CHIKV infection 5 days PTD (fever (103 F); joint and muscle pain; rash erythematous maculopapular; CHIKV IgM positive); Infants centrofacial hyperpigmentation persisted for 3 months |
| 21 | 2012 | Gopakumar J Clinical Neonatology 2012 | N=1 c-p CHIK  India (NR) | - Case of presumed c-pCHIKV with maternal positive CHIV IgM and IgG at delivery; but infant’s serology was negative for CHIKV, nevertheless the diagnosis of presumed c-p CHIKV infection was made based on clinical manifestations and strong epidemiologic profile - FT infant born via elective C/S to a mother with fever and severe multiple joints pains 2 days prior to delivery and diffuse skin hyperpigmentation 4 ds after symptom onset; Infant on DOL#3: High grade fever (103); DOL#5: generalized edema, tenderness, paradoxical cry; thrombocytopenia, acute renal failure; DOL#12: clinically relatively improved but persistence of thrombocytopenia, DOL#18: paradoxical cry; mild bleeding manifestations; lethargy; skin hyperpigmentation; oral mucositis-oral mucosal bleeding; elevated CRP; leukocytosis (WBC=28,000/mm3); lymphocytosis (80%); thrombocytopenia (18000/mm3); hyponateremia (129 mmol/L); transaminitis (AST=445 IU; ALT=220 IU);refractory shock suggestive of possible myocarditis; brain MRI with areas of bleeding in basal ganglia and subcortical areas; DOL#21: Discharged home with persistent paradoxical cry and irritability; probably due to persistent joint inflammation; At 6 month of age, infant clinically normal with fixed flexion deformity of right thumb (probably due to tenosynovitis). |
| 22 | 2014 | Evans-Gilbert Am J Trop Med 2017 | N=2 severe c-p CHIKV cases;  Jamaica; 2014 | - 35 wk old infant born via emergency C/S due to fetal distress from a Mother with 1 d Hx of fever, joint pain and abdominal pain PTD (MOP died on d#4 post OP from DIC, leukocytosis, acute renal failure and rhabdomyolysis; CHIKV RT-PCR positive); Infant with Mild respiratory distress at birth that resolved at 24 HOL; on DOL#3: cyanosis, apnea, hypoxia, hypotension (BP56/27); abdominal distension, coffee-ground aspirate; hypoglycemia; (hgb=14.3 g/dl; WBC=7,100/mm3; PLTs=203,000/ml); DOL#4: fever (101 F); poor perfusion (prolonged CRF=10 sec); worsening acrocyanosis; distal limb ischemia; worsening abdominal distension progressed to shock and died at DOL#4 ; RT-PCR of infant from DOL#1 positive for CHIKV - *Severe case of presumed c-p CHIKV but without serologic or molecular confirmation of CHIKV infection in the mother or the newborn: FT infant born via Vaginal delivery from a mother with Fever and Joint pain on the day of delivery (during CHIKV outbreak period); Infant on DOL #3: Fever, rash (generalized maculopapular), lethargy, poor feeding; abdominal distension, respiratory distress; acrocyanosis; peripheral ischemia in foot digits; leukopenia (WBC=4.1X10^9^/L and thrombocytopenia PLTS=96x10^9^/L; acidosis, direct hyperbilirubinemia; transaminitis (AST=707 IU/L and ALT=118 IU/L); on DOL#7: infant progressed to shock; developed anasarca edema, anuria, respiratory failure and died |
| 23 | 2014 | Kumar Indian Pediatrics 2014 | N=1 c-p CHIK;  India; (NR) | - Neonate born to a mother with Fever 7 ds PTD: presented during the 1^st^ week of life with Respiratory distress, thrombocytopenia, midfacial (brownie-nose) hyperpigmentation; Mother and infant were CHIKV IgM positive; Thrombocytopenia resolved by DOL#10 |
| 24 | 2015 | Villamil-Gomez J Tropical Pediatrics 2015 | N=8 (one pair of twins)  Colombia;  Sept 2014-Feb 2015 | - Eight newborn infants born to 7 mothers CHIKV RT-PCR and IgM positive; Infants presented with Maculopapular rash (n=5; 63%), hyperalgesia and, respiratory distress (n=4; 50%), sepsis, necrotizing enterocolitis and adenopathies (n=3; 38%), hemodynamic instability requiring volume expansion and vasopressors (n=3; 38%), meningoencephalitis, myocarditis and edema (n=2; 25%); need for mechanical ventilation (n=2; 25%); need for PLTs and FFP transfusion (n=3; 38%) bullous dermatitis (n=1; 13%). Death occurred in 3 /8 (case fatality rate: 38%), including the twins (2 of the infants who died had necrotizing enterocolitis and sepsis); (No congenital malformation identified) |
| 25 | 2016 | Alvarardo-Socarras JPIDS 2016 | N=1 c-p CHIK;  Colombia; (NR; within 2014-2016 period) | - FT infant born to mother with fever 1 day PTD; Infant with Fever at DOL #3; erythematous maculopapular rash; tachypnea; direct hyperbilirubinemia (Tbil=15.2 ; Dbili=14.8 mg/dl) and thrombocytopenia (PLTs=112,000/ml); Infant was Chik IgM positive; Mother not tested; resolution of symptoms within 9 days; normal neurodevelopmental outcome at 12 mo of age |
| 26 | 2016 | Bandiera ID cases 2016 | N=1 c-p CHIK;  Brazil; 2015 | - FT infant born via C/S to mother with disseminated rash and fever 4 days PTD; Infant with fever (37.9 C) at DOL #4; Rash (erythematous, macular), hypoactivity, generalized seizures; Brain MRI with hyperintensity signals on Diffusion weighted Images (DWI) in the subcortical white matter of frontal, parietal, temporal and corpus callosum; elevated CSF protein (106 mg/dl); hypoglycorachea (23 mg/dl); infant with positive RT-PCR for CHIKV |
| 27 | 2016 | Karthiga J Pediatric Neuroscience 2016 | N=2 c-p CHIK (twins)  India (NR) | - Newborn twin A 36 wk GA infant born via vaginal delivery to a mother with peripartum fever; DOL#5: Seizures (multiple episodes, despite correction of hypocalcemia), hypocalcemia, encephalopathy, persistent apnea requiring mechanical ventilation X 4 days; DOL#8: midfacial hyperpigmentation; Brain MRI: hemorrhagic leukoencephalopathy; (WBC=18,600/mm3; Anemia (hgb=8.3 g/dl); thrombocytopenia (PLTS=25,000/mmm3); mild uremia X 3 ds; Infant CHIV IgM positive; Mother CHIKV IgM positive; Twin B: also with similar clinical presentation and CHIKV IgM positive; Infant discharged home on DOL#24 - Newborn twin B (dichorionic diamniotic twin pregnancy): DOL#5 with multiple seizures; encephalopathy, required mechanical ventilation; midfacial hyperpigmentation, anemia, thrombocytopenia, Brain MRI: hemorrhagic leukoencephalopathy; CHIKV IgM positive; Infant discharged home on DOL#24 |
| 28 | 2016 | Laoprasopwattana Epidemiol Infect 2016 | N=0 c-p CHIK /88 recently infected pregnant women;  Thailand; 2009-2010 | - Not applicable (None of the newborns with recent CHIKV infection had positive CHIKV IgM in the cord blood (NR of additional subsequent neonatal serologic testing or neonatal clinical follow up)) |
| 29 | 2016 | Lyra Am J Perinatol Reports 2016 | N=2 c-p CHIK  Brazil, 8/2015-9/2015 | - Two case reports of infants born to mothers with presumed perinatally acquired CHIKV infection 2 ds PTD ; based on clinical symptoms but without serologic or molecular confirmations in the mothers or the infants - Mother 2 ds PTD had arthralgia (wrists, elbows, hips and knees), maculopapular rash in the abdomen; and the DOD developed fever (39 C) and chills. Infant born at 38 wks GA with meconium stained amniotic fluid; DOL#1 mild respiratory distress, developed pulmonary infiltrates, required mechanical ventilation; DOL#6: fever and maculopapular rash and hemodynamic instability requiring vasopressors; (DOL#20. Staph endocarditis with vegetations that were RT-PCR neg for CHIK); Infant discharged home on DOL#37 - Mother with presumed CHIKV 2 ds PTD: Infant born at 41 wks; DOL#4: Fever (37.9), lethargy, poor perfusion; also grade 1 IVH by cranial ultrasound (without lymphopenia, thrombocytopenia or hypoprothrombinemia); Infant discharged home on DOL#17 |
| 30 | 2016 | Pinzon-Redondo PIDJ 2016 | N=2 c-p CHIK included among other cases ; Colombia; 9-2014-12/2014 | - Not applicable (the clinical data for the two c-p CHIK cases were not reported separately from the 11 infants with CHIKV at <1 months of age) |
| 31 | 2016 | Rodrigues-Nieves PIDJ 2016 | N=10 c-p CHIK;  Puerto Rico; 8/2014-1/2015 | 10 newborn infants born to mothers with CHIKV like symptoms:   - In 7 newborns; their mothers with symptoms within 5 days PTD (70% of infants with intrapartum infections were symptomatic=higher than previously reported in the literature) - In 3 newborns: their mothers had symptoms >5 days PTD (not specified further timing of maternal infection during gestation) - In the 7/10 newborns whose mothers had symptoms within 5 days PTD: Irritability (50%), eczema (50%), fever (40%), malaise (40%), apnea (40%), tachypnea (40%), poor sucking (30%), cyanosis (30%), peripheral edema (30%), leukopenia (30%), leukocytosis (10%), thrombocytopenia (30%), low albumin (40%), elevated AST (40%), elevated ALT (10%), prolonged PR-interval (10%), prolonged PTT (40%) - In the 3/10 newborns whose mothers had symptoms >5 days PTD (not specified further timing of maternal infection during gestation): No fever, No Irritability BUT with congenital anomalies: Hydrocephalus and Brain Infarct |
| 32 | 2016 | Torres International J Infect Dis 2016 | N=169 symptomatic c-p CHIK;  2015 Outbreak  (El Salvador: N=51  Santo Domingo: N= 79; Colombia n=37) | - El Salvador (n=53): Fever (100%); poor feeding (98%); Irritability (91%), rash (85%), hyperalgesis/allodynia (94%), diffuse lower limb edema (87%), hemodynamic instability (53%), dermatosis bullosa/skin scaling (15%), respiratory failure (8%); meningoencephalitis (19%) - Santo Domingo (n=79): Fever (100%); poor feeding (100%); irritability (100%); rash (43%); Hyperalgesia/allodynia (57%); diffuse lower limb edema (42%); dermatosis bullosa (9%); respiratory failure (10%); meningoencephalitis (1%); hyperpigmentation (5%); myocarditis (1%) - Colombia (n=37): Fever (100%); poor feeding (97%); irritability (100%); rash (97%); Hyperalgesia/allodynia (14%); diffuse lower limb edema (27%); hemodynamic instability (3%); dermatosis bullosa (5%); respiratory failure (3%); meningoencephalitis (3%); myocarditis (5%) |
| 33 | 2016 | Vasani Pediatric Dermatology 2016 | N=1 c-p CHIK  India; (NR) | - Full term newborn born to a mother with high grade fever 7 ds PTD, which continued also until 3 days post-delivery (no joint pains or rashes) Mother on day#9 post-delivery developed and blackish discoloration over the nose, lips, streaky pigmentation over the trunk and patchy pigmentation over the extremities; Infant at Birth: mild respiratory distress which resolved by DOL 6 and mild acrocyanosis (without hemodynamic instability; Infant discharged home on DOL#6; DOL#9: Hyperpigmentation (over nose, extension in upper and lower lips; few freckled like macules in alar area bilaterally; flagellate hypergpigmentation over the sides of the trunk; hyperpigmented patches over the dorsa of hands, ankles; groins; penile shaft and scrotum); Infant and Mother CHIKV IgM positive; Hyperpigmentation improved of midface after 6 weeks |

**Abbreviations:** APRF: antepartum fetal deaths; CHIKV: chikungunya virus; c-p-CHIKV: congenital-perinatal CHIKV infections; CP: cerebral palsy; DIC: disseminated intravascular coagulation; DOL: day of life; FT: full term; GA: gestational age; PTD: prior to delivery; c-p-CHIK: congenital-perinatal Chikungunya virus infections; wks: weeks

REFERENCES FOR SUPPLEMENTARY MATERIAL

1. Gerardin P, Barau G, Michault A, Bintner M, Randrianaivo H, Choker G, et al. Multidisciplinary prospective study of mother-to-child chikungunya virus infections on the Island of La Reunion. Plos Med. 2008;5(3):413-23. doi: ARTN 060

10.1371/journal.pmed.0050060. PubMed PMID: WOS:000254928900016.

2. Touret Y, Randrianaivo H, Michault A, Schuffenecker I, Kauffmann E, Lenglet Y, et al. Early maternal-fetal transmission of the Chikungunya virus. Presse Med. 2006;35(11):1656-8. doi: Doi 10.1016/S0755-4982(06)74874-6. PubMed PMID: WOS:000242164400010.

3. Robin S, Rainful D, Le Seach F, Jaffar-Bandjee MC, Rigou G, Alessandri JL. Neurologic manifestations of pediatric chikungunya infection. J Child Neurol. 2008;23(9):1028-35. doi: 10.1177/0883073808314151. PubMed PMID: WOS:000258841800007.

4. Robillard PY, Boumahni B, Gerardin P, Michault A, Fourmaintraux A, Schuffenecker I, et al. Vertical maternal fetal transmission of the chikungunya virus - Ten cases among 84 pregnant women. Presse Med. 2006;35(5):785-8. doi: Doi 10.1016/S0755-4982(06)74690-5. PubMed PMID: WOS:000237918800011.

5. Ramful D, Carbonnier M, Pasquet M, Bouhmani B, Ghazouani J, Noormahomed T, et al. Mother-to-child transmission of Chikungunya virus infection. Pediatr Infect Dis J. 2007;26(9):811-5. doi: 10.1097/INF.0b013e3180616d4f. PubMed PMID: WOS:000249455800008.

6. Lenglet Y, Barau G, Robillard PY, Randrianaivo H, Michault A, Bouveret A, et al. [Chikungunya infection in pregnancy: Evidence for intrauterine infection in pregnant women and vertical transmission in the parturient. Survey of the Reunion Island outbreak]. J Gynecol Obstet Biol Reprod (Paris). 2006;35(6):578-83. PubMed PMID: 17003745.

7. Ramful D, Samperiz S, Fritel X, Michault A, Jaffar-Bandjee MC, Rollot O, et al. Antibody kinetics in infants exposed to Chikungunya virus infection during pregnancy reveals absence of congenital infection. J Infect Dis. 2014;209(11):1726-30. doi: 10.1093/infdis/jit814. PubMed PMID: 24338351.

8. Gerardin P, Couderc T, Randrianaivo H, Fritel X, Lecuit M. CHIKUNGUNYA VIRUS-ASSOCIATED ENCEPHALITIS: A COHORT STUDY ON LA REUNION ISLAND, 2005-2009 Response. Neurology. 2016;86(21):2025-6. PubMed PMID: WOS:000376959900023.

9. Gerardin P, Samperiz S, Ramful D, Boumahni B, Bintner M, Alessandri JL, et al. Neurocognitive outcome of children exposed to perinatal mother-to-child Chikungunya virus infection: the CHIMERE cohort study on Reunion Island. PLoS Negl Trop Dis. 2014;8(7):e2996. doi: 10.1371/journal.pntd.0002996. PubMed PMID: 25033077; PubMed Central PMCID: PMCPMC4102444.

10. Fritel X, Rollot O, Gerardin P, Gauzere BA, Bideault J, Lagarde L, et al. Chikungunya virus infection during pregnancy, Reunion, France, 2006. Emerg Infect Dis. 2010;16(3):418-25. doi: 10.3201/eid1603.091403. PubMed PMID: 20202416; PubMed Central PMCID: PMCPMC3322036.

11. Boumahni B, Kaplan C, Clabe A, Randrianaivo H, Lanza F. Maternal-fetal chikungunya infection associated with Bernard-Soulier syndrome. Arch Pediatrie. 2011;18(3):272-5. doi: 10.1016/j.arcped.2010.12.002. PubMed PMID: WOS:000288186400006.

12. Alvarado-Socarras JL, Ocampo-Gonzalez M, Vargas-Soler JA, Rodriguez-Morales AJ, Franco-Paredes C. Congenital and Neonatal Chikungunya in Colombia. J Pediatr Infect Dis. 2016;5(3):E17-E20. doi: 10.1093/jpids/piw021. PubMed PMID: WOS:000386138100001.

13. Bandeira AC, Campos GS, Sardi SI, Rocha VFD, Rocha GCM. Neonatal encephalitis due to Chikungunya vertical transmission: First report in Brazil. IDCases. 2016;5:57-9. doi: 10.1016/j.idcr.2016.07.008. PubMed PMID: WOS:000399150800019.

14. Evans-Gilbert T. Case Report: Chikungunya and Neonatal Immunity: Fatal Vertically Transmitted Chikungunya Infection. Am J Trop Med Hyg. 2017;96(4):913-5. doi: 10.4269/ajtmh.16-0491. PubMed PMID: WOS:000401763000027.

15. Karthiga V, Kommu PPK, Krishnan L. Perinatal chikungunya in twins. J Pediatr Neurosci. 2016;11(3):223-4. doi: 10.4103/1817-1745.193369. PubMed PMID: WOS:000390115700012.

16. Khandelwal K, Aara N, Ghiya BC, Bumb RA, Satoskar AR. Centro-Facial Pigmentation in Asymptomatic Congenital Chikungunya Viral Infection. J Paediatr Child H. 2012;48(6):542-3. doi: 10.1111/j.1440-1754.2012.02484.x. PubMed PMID: WOS:000305186200021.

17. Kumar N, Gupta V, Thomas N. Brownie-nose: Hyperpigmentation in Neonatal Chikungunya. Indian Pediatr. 2014;51(5):419-. PubMed PMID: WOS:000336049800023.

18. Laoprasopwattana K, Suntharasaj T, Petmanee P, Suddeaugrai O, Geater A. Chikungunya and dengue virus infections during pregnancy: seroprevalence, seroincidence and maternal-fetal transmission, southern Thailand, 2009-2010. Epidemiol Infect. 2016;144(2):381-8. doi: 10.1017/S0950268815001065. PubMed PMID: WOS:000368638100020.

19. Lyra PPR, Campos GS, Bandeira ID, Sardi SI, Costa LFD, Santos FR, et al. Congenital Chikungunya Virus Infection after an Outbreak in Salvador, Bahia, Brazil. Ajp Rep. 2016;6(3):E299-E300. doi: 10.1055/s-0036-1587323. PubMed PMID: WOS:000382531200008.

20. Passi GR, Khan YZ, Chitnis DS. Chikungunya infection in neonates. Indian Pediatr. 2008;45(3):240-2. PubMed PMID: WOS:000254357300016.

21. Boumahni B, Bintner M. [Five-year outcome of mother-to-child transmission of chikungunya virus]. Med Trop (Mars). 2012;72 Spec No:94-6. PubMed PMID: 22693938.

22. Pinzon-Redondo H, Paternina-Caicedo A, Barrios-Redondo K, Zarate-Vergara A, Tirado-Perez I, Fortich R, et al. RISK FACTORS FOR SEVERITY OF CHIKUNGUNYA IN CHILDREN A Prospective Assessment. Pediatr Infect Dis J. 2016;35(6):702-4. doi: 10.1097/Inf.0000000000001135. PubMed PMID: WOS:000379343700024.

23. Senanayake MP SS, Vidanage KK, Gunassena S, Lamabadusurlya SP. Vertical transmission in Chikungunya infection. Cylon Med J. 2009;54(2):47-50.

24. Shenoy S, Pradeep GCM. Neurodevelopmental Outcome of Neonates with Vertically Transmitted Chikungunya Fever with Encephalopathy. Indian Pediatr. 2012;49(3):238-40. PubMed PMID: WOS:000304110800015.

25. Shrivastava A, Beg MW, Gujrati C, Gopalan N, Rao PVL. Management of a Vertically Transmitted Neonatal Chikungunya Thrombocytopenia. Indian J Pediatr. 2011;78(8):1008-9. doi: 10.1007/s12098-011-0371-7. PubMed PMID: WOS:000293143700015.

26. Sissoko D, Malvy D, Giry C, Delmas G, Paquet C, Gabrie P, et al. Outbreak of Chikungunya fever in Mayotte, Comoros archipelago, 2005-2006. T Roy Soc Trop Med H. 2008;102(8):780-6. doi: 10.1016/j.trstmh.2008.02.018. PubMed PMID: WOS:000258201600008.

27. Torres JR, Falleiros-Arlant LH, Duenas L, Pleitez-Navarrete J, Salgado DM, Brea-Del Castillo J. Congenital and perinatal complications of chikungunya fever: a Latin American experience. Int J Infect Dis. 2016;51:85-8. doi: 10.1016/j.ijid.2016.09.009. PubMed PMID: WOS:000388326700020.

28. Valamparampil JJ, Chirakkarot S, Letha S, Jayakumar C, Gopinathan KM. Clinical profile of Chikungunya in infants. Indian J Pediatr. 2009;76(2):151-5. doi: 10.1007/s12098-009-0045-x. PubMed PMID: WOS:000264631100003.

29. Vasani R, Kanhere S, Chaudhari K, Phadke V, Mukherjee P, Gupta S, et al. Congenital Chikungunya-A Cause of Neonatal Hyperpigmentation. Pediatr Dermatol. 2016;33(2):209-12. doi: 10.1111/pde.12650. PubMed PMID: WOS:000373067800055.

30. Villamil-Gomez W, Alba-Silvera L, Menco-Ramos A, Gonzalez-Vergara A, Molinares-Palacios T, Barrios-Corrales M, et al. Congenital Chikungunya Virus Infection in Sincelejo, Colombia: A Case Series. J Trop Pediatrics. 2015;61(5):386-92. doi: 10.1093/tropej/fmv051. PubMed PMID: WOS:000365384300010.

31. Rodriguez-Nieves M, Garcia-Garcia I, Garcia-Fragoso L. Perinatally Acquired Chikungunya Infection: The Puerto Rico Experience. Pediatr Infect Dis J. 2016;35(10):1163. doi: 10.1097/INF.0000000000001261. PubMed PMID: 27622689.

32. Gopakumar H, Ramachandran S. Congenital chikungunya. J Clin Neonatol. 2012;1(3):155-6. doi: 10.4103/2249-4847.101704. PubMed PMID: 24027715; PubMed Central PMCID: PMCPMC3762016.

1. Lenglet Y, Barau G, Robillard PY, Randrianaivo H, Michault A, Bouveret A, et al. [Chikungunya infection in pregnancy: Evidence for intrauterine infection in pregnant women and vertical transmission in the parturient. Survey of the Reunion Island outbreak]. J Gynecol Obstet Biol Reprod (Paris). 2006;35(6):578-83. PubMed PMID: 17003745.

2. Robillard PY, Boumahni B, Gerardin P, Michault A, Fourmaintraux A, Schuffenecker I, et al. Vertical maternal fetal transmission of the chikungunya virus - Ten cases among 84 pregnant women. Presse Med. 2006;35(5):785-8. doi: Doi 10.1016/S0755-4982(06)74690-5. PubMed PMID: WOS:000237918800011.

3. Ramful D, Carbonnier M, Pasquet M, Bouhmani B, Ghazouani J, Noormahomed T, et al. Mother-to-child transmission of Chikungunya virus infection. Pediatr Infect Dis J. 2007;26(9):811-5. doi: 10.1097/INF.0b013e3180616d4f. PubMed PMID: WOS:000249455800008.

4. Gerardin P, Barau G, Michault A, Bintner M, Randrianaivo H, Choker G, et al. Multidisciplinary prospective study of mother-to-child chikungunya virus infections on the Island of La Reunion. Plos Med. 2008;5(3):413-23. doi: ARTN 060

10.1371/journal.pmed.0050060. PubMed PMID: WOS:000254928900016.

5. Fritel X, Rollot O, Gerardin P, Gauzere BA, Bideault J, Lagarde L, et al. Chikungunya virus infection during pregnancy, Reunion, France, 2006. Emerg Infect Dis. 2010;16(3):418-25. doi: 10.3201/eid1603.091403. PubMed PMID: 20202416; PubMed Central PMCID: PMCPMC3322036.

6. Ramful D, Samperiz S, Fritel X, Michault A, Jaffar-Bandjee MC, Rollot O, et al. Antibody kinetics in infants exposed to Chikungunya virus infection during pregnancy reveals absence of congenital infection. J Infect Dis. 2014;209(11):1726-30. doi: 10.1093/infdis/jit814. PubMed PMID: 24338351.

7. Gerardin P, Samperiz S, Ramful D, Boumahni B, Bintner M, Alessandri JL, et al. Neurocognitive outcome of children exposed to perinatal mother-to-child Chikungunya virus infection: the CHIMERE cohort study on Reunion Island. PLoS Negl Trop Dis. 2014;8(7):e2996. doi: 10.1371/journal.pntd.0002996. PubMed PMID: 25033077; PubMed Central PMCID: PMCPMC4102444.

8. Sissoko D, Malvy D, Giry C, Delmas G, Paquet C, Gabrie P, et al. Outbreak of Chikungunya fever in Mayotte, Comoros archipelago, 2005-2006. T Roy Soc Trop Med H. 2008;102(8):780-6. doi: 10.1016/j.trstmh.2008.02.018. PubMed PMID: WOS:000258201600008.

9. Watanaveeradej V, Endy TP, Simasathien S, Kerdpanich A, Polprasert N, Aree C, et al. Transplacental chikungunya virus antibody kinetics, Thailand. Emerg Infect Dis. 2006;12(11):1770-2. PubMed PMID: WOS:000241573900025.

10. Senanayake MP SS, Vidanage KK, Gunassena S, Lamabadusurlya SP. Vertical transmission in Chikungunya infection. Cylon Med J. 2009;54(2):47-50.

11. Laoprasopwattana K, Suntharasaj T, Petmanee P, Suddeaugrai O, Geater A. Chikungunya and dengue virus infections during pregnancy: seroprevalence, seroincidence and maternal-fetal transmission, southern Thailand, 2009-2010. Epidemiol Infect. 2016;144(2):381-8. doi: 10.1017/S0950268815001065. PubMed PMID: WOS:000368638100020.

12. Torres JR, Falleiros-Arlant LH, Duenas L, Pleitez-Navarrete J, Salgado DM, Brea-Del Castillo J. Congenital and perinatal complications of chikungunya fever: a Latin American experience. Int J Infect Dis. 2016;51:85-8. doi: 10.1016/j.ijid.2016.09.009. PubMed PMID: WOS:000388326700020.

13. Escobar M, Nieto AJ, Loaiza-Osorio S, Barona JS, Rosso F. Pregnant Women Hospitalized with Chikungunya Virus Infection, Colombia, 2015. Emerg Infect Dis. 2017;23(11):1777-83. doi: 10.3201/eid2311.170480. PubMed PMID: WOS:000413109500002.

14. Touret Y, Randrianaivo H, Michault A, Schuffenecker I, Kauffmann E, Lenglet Y, et al. Early maternal-fetal transmission of the Chikungunya virus. Presse Med. 2006;35(11):1656-8. doi: Doi 10.1016/S0755-4982(06)74874-6. PubMed PMID: WOS:000242164400010.

15. Robin S, Rainful D, Le Seach F, Jaffar-Bandjee MC, Rigou G, Alessandri JL. Neurologic manifestations of pediatric chikungunya infection. J Child Neurol. 2008;23(9):1028-35. doi: 10.1177/0883073808314151. PubMed PMID: WOS:000258841800007.

16. Gerardin P, Couderc T, Randrianaivo H, Fritel X, Lecuit M. CHIKUNGUNYA VIRUS-ASSOCIATED ENCEPHALITIS: A COHORT STUDY ON LA REUNION ISLAND, 2005-2009 Response. Neurology. 2016;86(21):2025-6. PubMed PMID: WOS:000376959900023.

17. Boumahni B, Kaplan C, Clabe A, Randrianaivo H, Lanza F. Maternal-fetal chikungunya infection associated with Bernard-Soulier syndrome. Arch Pediatrie. 2011;18(3):272-5. doi: 10.1016/j.arcped.2010.12.002. PubMed PMID: WOS:000288186400006.

18. Alvarado-Socarras JL, Ocampo-Gonzalez M, Vargas-Soler JA, Rodriguez-Morales AJ, Franco-Paredes C. Congenital and Neonatal Chikungunya in Colombia. J Pediatr Infect Dis. 2016;5(3):E17-E20. doi: 10.1093/jpids/piw021. PubMed PMID: WOS:000386138100001.

19. Bandeira AC, Campos GS, Sardi SI, Rocha VFD, Rocha GCM. Neonatal encephalitis due to Chikungunya vertical transmission: First report in Brazil. IDCases. 2016;5:57-9. doi: 10.1016/j.idcr.2016.07.008. PubMed PMID: WOS:000399150800019.

20. Evans-Gilbert T. Case Report: Chikungunya and Neonatal Immunity: Fatal Vertically Transmitted Chikungunya Infection. Am J Trop Med Hyg. 2017;96(4):913-5. doi: 10.4269/ajtmh.16-0491. PubMed PMID: WOS:000401763000027.

21. Karthiga V, Kommu PPK, Krishnan L. Perinatal chikungunya in twins. J Pediatr Neurosci. 2016;11(3):223-4. doi: 10.4103/1817-1745.193369. PubMed PMID: WOS:000390115700012.

22. Khandelwal K, Aara N, Ghiya BC, Bumb RA, Satoskar AR. Centro-Facial Pigmentation in Asymptomatic Congenital Chikungunya Viral Infection. J Paediatr Child H. 2012;48(6):542-3. doi: 10.1111/j.1440-1754.2012.02484.x. PubMed PMID: WOS:000305186200021.

23. Kumar N, Gupta V, Thomas N. Brownie-nose: Hyperpigmentation in Neonatal Chikungunya. Indian Pediatr. 2014;51(5):419-. PubMed PMID: WOS:000336049800023.

24. Lyra PPR, Campos GS, Bandeira ID, Sardi SI, Costa LFD, Santos FR, et al. Congenital Chikungunya Virus Infection after an Outbreak in Salvador, Bahia, Brazil. Ajp Rep. 2016;6(3):E299-E300. doi: 10.1055/s-0036-1587323. PubMed PMID: WOS:000382531200008.

25. Passi GR, Khan YZ, Chitnis DS. Chikungunya infection in neonates. Indian Pediatr. 2008;45(3):240-2. PubMed PMID: WOS:000254357300016.

26. Boumahni B, Bintner M. [Five-year outcome of mother-to-child transmission of chikungunya virus]. Med Trop (Mars). 2012;72 Spec No:94-6. PubMed PMID: 22693938.

27. Pinzon-Redondo H, Paternina-Caicedo A, Barrios-Redondo K, Zarate-Vergara A, Tirado-Perez I, Fortich R, et al. RISK FACTORS FOR SEVERITY OF CHIKUNGUNYA IN CHILDREN A Prospective Assessment. Pediatr Infect Dis J. 2016;35(6):702-4. doi: 10.1097/Inf.0000000000001135. PubMed PMID: WOS:000379343700024.

28. Shenoy S, Pradeep GCM. Neurodevelopmental Outcome of Neonates with Vertically Transmitted Chikungunya Fever with Encephalopathy. Indian Pediatr. 2012;49(3):238-40. PubMed PMID: WOS:000304110800015.

29. Shrivastava A, Beg MW, Gujrati C, Gopalan N, Rao PVL. Management of a Vertically Transmitted Neonatal Chikungunya Thrombocytopenia. Indian J Pediatr. 2011;78(8):1008-9. doi: 10.1007/s12098-011-0371-7. PubMed PMID: WOS:000293143700015.

30. Valamparampil JJ, Chirakkarot S, Letha S, Jayakumar C, Gopinathan KM. Clinical profile of Chikungunya in infants. Indian J Pediatr. 2009;76(2):151-5. doi: 10.1007/s12098-009-0045-x. PubMed PMID: WOS:000264631100003.

31. Vasani R, Kanhere S, Chaudhari K, Phadke V, Mukherjee P, Gupta S, et al. Congenital Chikungunya-A Cause of Neonatal Hyperpigmentation. Pediatr Dermatol. 2016;33(2):209-12. doi: 10.1111/pde.12650. PubMed PMID: WOS:000373067800055.

32. Villamil-Gomez W, Alba-Silvera L, Menco-Ramos A, Gonzalez-Vergara A, Molinares-Palacios T, Barrios-Corrales M, et al. Congenital Chikungunya Virus Infection in Sincelejo, Colombia: A Case Series. J Trop Pediatrics. 2015;61(5):386-92. doi: 10.1093/tropej/fmv051. PubMed PMID: WOS:000365384300010.

33. Rodriguez-Nieves M, Garcia-Garcia I, Garcia-Fragoso L. Perinatally Acquired Chikungunya Infection: The Puerto Rico Experience. Pediatr Infect Dis J. 2016;35(10):1163. doi: 10.1097/INF.0000000000001261. PubMed PMID: 27622689.

34. Gopakumar H, Ramachandran S. Congenital chikungunya. J Clin Neonatol. 2012;1(3):155-6. doi: 10.4103/2249-4847.101704. PubMed PMID: 24027715; PubMed Central PMCID: PMCPMC3762016.

1. Lenglet Y, Barau G, Robillard PY, Randrianaivo H, Michault A, Bouveret A, et al. [Chikungunya infection in pregnancy: Evidence for intrauterine infection in pregnant women and vertical transmission in the parturient. Survey of the Reunion Island outbreak]. J Gynecol Obstet Biol Reprod (Paris). 2006;35(6):578-83. PubMed PMID: 17003745.

2. Robillard PY, Boumahni B, Gerardin P, Michault A, Fourmaintraux A, Schuffenecker I, et al. Vertical maternal fetal transmission of the chikungunya virus - Ten cases among 84 pregnant women. Presse Med. 2006;35(5):785-8. doi: Doi 10.1016/S0755-4982(06)74690-5. PubMed PMID: WOS:000237918800011.

3. Ramful D, Carbonnier M, Pasquet M, Bouhmani B, Ghazouani J, Noormahomed T, et al. Mother-to-child transmission of Chikungunya virus infection. Pediatr Infect Dis J. 2007;26(9):811-5. doi: 10.1097/INF.0b013e3180616d4f. PubMed PMID: WOS:000249455800008.

4. Gerardin P, Barau G, Michault A, Bintner M, Randrianaivo H, Choker G, et al. Multidisciplinary prospective study of mother-to-child chikungunya virus infections on the Island of La Reunion. Plos Med. 2008;5(3):413-23. doi: ARTN 060

10.1371/journal.pmed.0050060. PubMed PMID: WOS:000254928900016.

5. Fritel X, Rollot O, Gerardin P, Gauzere BA, Bideault J, Lagarde L, et al. Chikungunya virus infection during pregnancy, Reunion, France, 2006. Emerg Infect Dis. 2010;16(3):418-25. doi: 10.3201/eid1603.091403. PubMed PMID: 20202416; PubMed Central PMCID: PMCPMC3322036.

6. Ramful D, Samperiz S, Fritel X, Michault A, Jaffar-Bandjee MC, Rollot O, et al. Antibody kinetics in infants exposed to Chikungunya virus infection during pregnancy reveals absence of congenital infection. J Infect Dis. 2014;209(11):1726-30. doi: 10.1093/infdis/jit814. PubMed PMID: 24338351.

7. Gerardin P, Samperiz S, Ramful D, Boumahni B, Bintner M, Alessandri JL, et al. Neurocognitive outcome of children exposed to perinatal mother-to-child Chikungunya virus infection: the CHIMERE cohort study on Reunion Island. PLoS Negl Trop Dis. 2014;8(7):e2996. doi: 10.1371/journal.pntd.0002996. PubMed PMID: 25033077; PubMed Central PMCID: PMCPMC4102444.

8. Sissoko D, Malvy D, Giry C, Delmas G, Paquet C, Gabrie P, et al. Outbreak of Chikungunya fever in Mayotte, Comoros archipelago, 2005-2006. T Roy Soc Trop Med H. 2008;102(8):780-6. doi: 10.1016/j.trstmh.2008.02.018. PubMed PMID: WOS:000258201600008.

9. Watanaveeradej V, Endy TP, Simasathien S, Kerdpanich A, Polprasert N, Aree C, et al. Transplacental chikungunya virus antibody kinetics, Thailand. Emerg Infect Dis. 2006;12(11):1770-2. PubMed PMID: WOS:000241573900025.

10. Senanayake MP SS, Vidanage KK, Gunassena S, Lamabadusurlya SP. Vertical transmission in Chikungunya infection. Cylon Med J. 2009;54(2):47-50.

11. Laoprasopwattana K, Suntharasaj T, Petmanee P, Suddeaugrai O, Geater A. Chikungunya and dengue virus infections during pregnancy: seroprevalence, seroincidence and maternal-fetal transmission, southern Thailand, 2009-2010. Epidemiol Infect. 2016;144(2):381-8. doi: 10.1017/S0950268815001065. PubMed PMID: WOS:000368638100020.

12. Torres JR, Falleiros-Arlant LH, Duenas L, Pleitez-Navarrete J, Salgado DM, Brea-Del Castillo J. Congenital and perinatal complications of chikungunya fever: a Latin American experience. Int J Infect Dis. 2016;51:85-8. doi: 10.1016/j.ijid.2016.09.009. PubMed PMID: WOS:000388326700020.

13. Escobar M, Nieto AJ, Loaiza-Osorio S, Barona JS, Rosso F. Pregnant Women Hospitalized with Chikungunya Virus Infection, Colombia, 2015. Emerg Infect Dis. 2017;23(11):1777-83. doi: 10.3201/eid2311.170480. PubMed PMID: WOS:000413109500002.

14. Touret Y, Randrianaivo H, Michault A, Schuffenecker I, Kauffmann E, Lenglet Y, et al. Early maternal-fetal transmission of the Chikungunya virus. Presse Med. 2006;35(11):1656-8. doi: Doi 10.1016/S0755-4982(06)74874-6. PubMed PMID: WOS:000242164400010.

15. Robin S, Rainful D, Le Seach F, Jaffar-Bandjee MC, Rigou G, Alessandri JL. Neurologic manifestations of pediatric chikungunya infection. J Child Neurol. 2008;23(9):1028-35. doi: 10.1177/0883073808314151. PubMed PMID: WOS:000258841800007.

16. Gerardin P, Couderc T, Randrianaivo H, Fritel X, Lecuit M. CHIKUNGUNYA VIRUS-ASSOCIATED ENCEPHALITIS: A COHORT STUDY ON LA REUNION ISLAND, 2005-2009 Response. Neurology. 2016;86(21):2025-6. PubMed PMID: WOS:000376959900023.

17. Boumahni B, Kaplan C, Clabe A, Randrianaivo H, Lanza F. Maternal-fetal chikungunya infection associated with Bernard-Soulier syndrome. Arch Pediatrie. 2011;18(3):272-5. doi: 10.1016/j.arcped.2010.12.002. PubMed PMID: WOS:000288186400006.

18. Alvarado-Socarras JL, Ocampo-Gonzalez M, Vargas-Soler JA, Rodriguez-Morales AJ, Franco-Paredes C. Congenital and Neonatal Chikungunya in Colombia. J Pediatr Infect Dis. 2016;5(3):E17-E20. doi: 10.1093/jpids/piw021. PubMed PMID: WOS:000386138100001.

19. Bandeira AC, Campos GS, Sardi SI, Rocha VFD, Rocha GCM. Neonatal encephalitis due to Chikungunya vertical transmission: First report in Brazil. IDCases. 2016;5:57-9. doi: 10.1016/j.idcr.2016.07.008. PubMed PMID: WOS:000399150800019.

20. Evans-Gilbert T. Case Report: Chikungunya and Neonatal Immunity: Fatal Vertically Transmitted Chikungunya Infection. Am J Trop Med Hyg. 2017;96(4):913-5. doi: 10.4269/ajtmh.16-0491. PubMed PMID: WOS:000401763000027.

21. Karthiga V, Kommu PPK, Krishnan L. Perinatal chikungunya in twins. J Pediatr Neurosci. 2016;11(3):223-4. doi: 10.4103/1817-1745.193369. PubMed PMID: WOS:000390115700012.

22. Khandelwal K, Aara N, Ghiya BC, Bumb RA, Satoskar AR. Centro-Facial Pigmentation in Asymptomatic Congenital Chikungunya Viral Infection. J Paediatr Child H. 2012;48(6):542-3. doi: 10.1111/j.1440-1754.2012.02484.x. PubMed PMID: WOS:000305186200021.

23. Kumar N, Gupta V, Thomas N. Brownie-nose: Hyperpigmentation in Neonatal Chikungunya. Indian Pediatr. 2014;51(5):419-. PubMed PMID: WOS:000336049800023.

24. Lyra PPR, Campos GS, Bandeira ID, Sardi SI, Costa LFD, Santos FR, et al. Congenital Chikungunya Virus Infection after an Outbreak in Salvador, Bahia, Brazil. Ajp Rep. 2016;6(3):E299-E300. doi: 10.1055/s-0036-1587323. PubMed PMID: WOS:000382531200008.

25. Passi GR, Khan YZ, Chitnis DS. Chikungunya infection in neonates. Indian Pediatr. 2008;45(3):240-2. PubMed PMID: WOS:000254357300016.

26. Boumahni B, Bintner M. [Five-year outcome of mother-to-child transmission of chikungunya virus]. Med Trop (Mars). 2012;72 Spec No:94-6. PubMed PMID: 22693938.

27. Pinzon-Redondo H, Paternina-Caicedo A, Barrios-Redondo K, Zarate-Vergara A, Tirado-Perez I, Fortich R, et al. RISK FACTORS FOR SEVERITY OF CHIKUNGUNYA IN CHILDREN A Prospective Assessment. Pediatr Infect Dis J. 2016;35(6):702-4. doi: 10.1097/Inf.0000000000001135. PubMed PMID: WOS:000379343700024.

28. Shenoy S, Pradeep GCM. Neurodevelopmental Outcome of Neonates with Vertically Transmitted Chikungunya Fever with Encephalopathy. Indian Pediatr. 2012;49(3):238-40. PubMed PMID: WOS:000304110800015.

29. Shrivastava A, Beg MW, Gujrati C, Gopalan N, Rao PVL. Management of a Vertically Transmitted Neonatal Chikungunya Thrombocytopenia. Indian J Pediatr. 2011;78(8):1008-9. doi: 10.1007/s12098-011-0371-7. PubMed PMID: WOS:000293143700015.

30. Valamparampil JJ, Chirakkarot S, Letha S, Jayakumar C, Gopinathan KM. Clinical profile of Chikungunya in infants. Indian J Pediatr. 2009;76(2):151-5. doi: 10.1007/s12098-009-0045-x. PubMed PMID: WOS:000264631100003.

31. Vasani R, Kanhere S, Chaudhari K, Phadke V, Mukherjee P, Gupta S, et al. Congenital Chikungunya-A Cause of Neonatal Hyperpigmentation. Pediatr Dermatol. 2016;33(2):209-12. doi: 10.1111/pde.12650. PubMed PMID: WOS:000373067800055.

32. Villamil-Gomez W, Alba-Silvera L, Menco-Ramos A, Gonzalez-Vergara A, Molinares-Palacios T, Barrios-Corrales M, et al. Congenital Chikungunya Virus Infection in Sincelejo, Colombia: A Case Series. J Trop Pediatrics. 2015;61(5):386-92. doi: 10.1093/tropej/fmv051. PubMed PMID: WOS:000365384300010.

33. Rodriguez-Nieves M, Garcia-Garcia I, Garcia-Fragoso L. Perinatally Acquired Chikungunya Infection: The Puerto Rico Experience. Pediatr Infect Dis J. 2016;35(10):1163. doi: 10.1097/INF.0000000000001261. PubMed PMID: 27622689.

34. Gopakumar H, Ramachandran S. Congenital chikungunya. J Clin Neonatol. 2012;1(3):155-6. doi: 10.4103/2249-4847.101704. PubMed PMID: 24027715; PubMed Central PMCID: PMCPMC3762016.
